# Supplementary material for: Rational development of catalytic Au(I)/Au(III) arylation involving mild oxidative addition of aryl halides
Source: Nat Commun. 2017 Sep 18;8:565. doi: 10.1038/s41467-017-00672-8 (PMC5603523; doi:10.1038/s41467-017-00672-8)
Supplement: Supplementary file 1 — Supplementary Information [file 41467_2017_672_MOESM1_ESM.pdf]

Description of Supplementary Files

File Name: Supplementary Information
Description: Supplementary Figures, Supplementary Tables, Supplementary Methods, Supplementary Discussion and Supplementary References

File Name: Peer Review File

Supplementary Figures

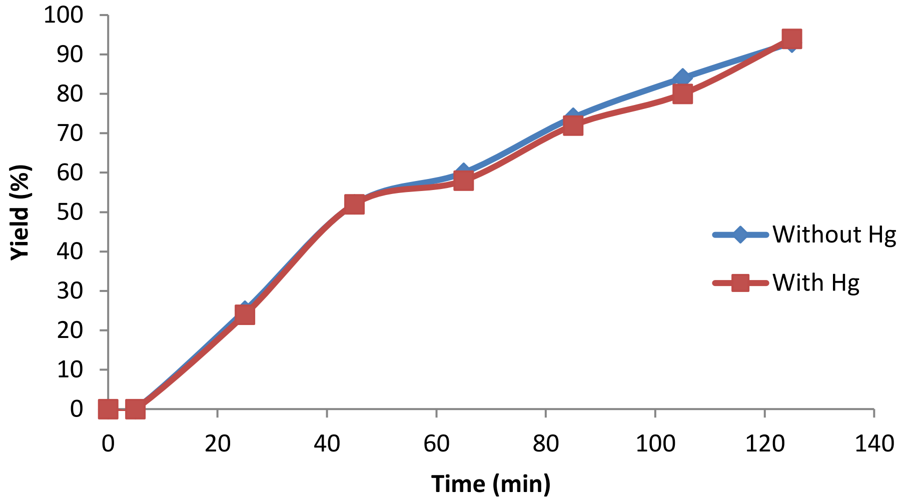

Supplementary Figure 1. Mercury poisoning control. Monitoring of the 1-iodo-4-nitrobenzene / 1,3,5 trimethoxybenzene coupling in the presence or not of Hg.

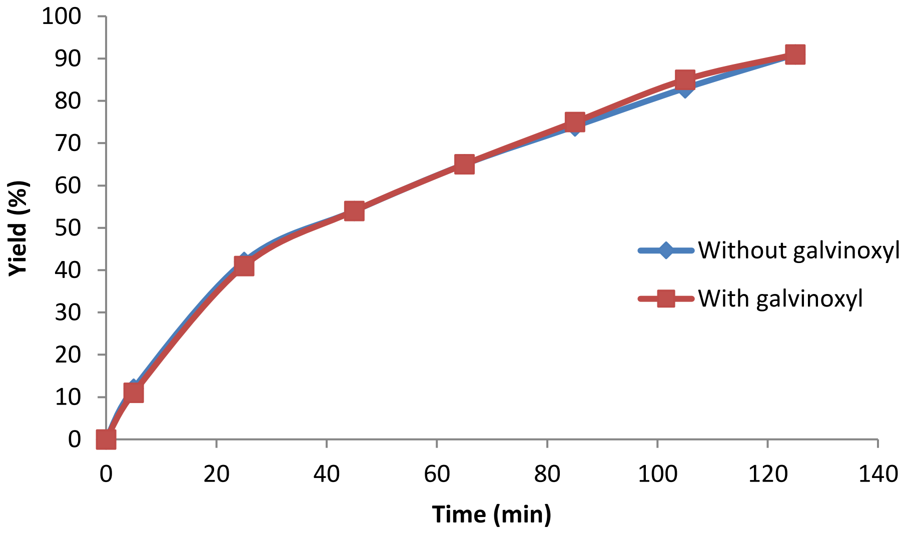

Supplementary Figure 2. Radical scavenger control. Monitoring of the 1-iodo-4-nitrobenzene / 1,3,5 trimethoxybenzene coupling in the presence or not of galvinoxyl.

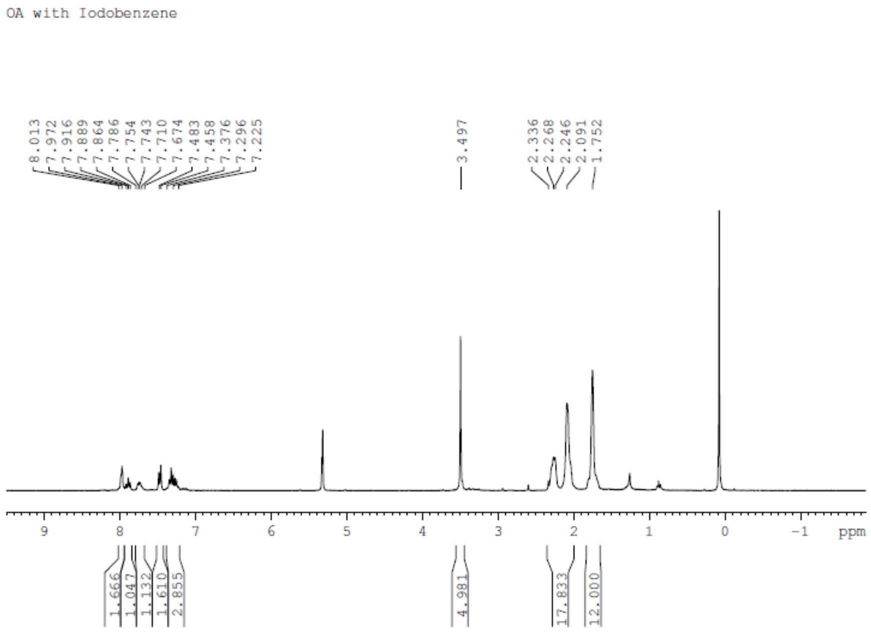

Supplementary Figure 3. ^{1}H NMR spectrum of (2) in CD_2Cl_2

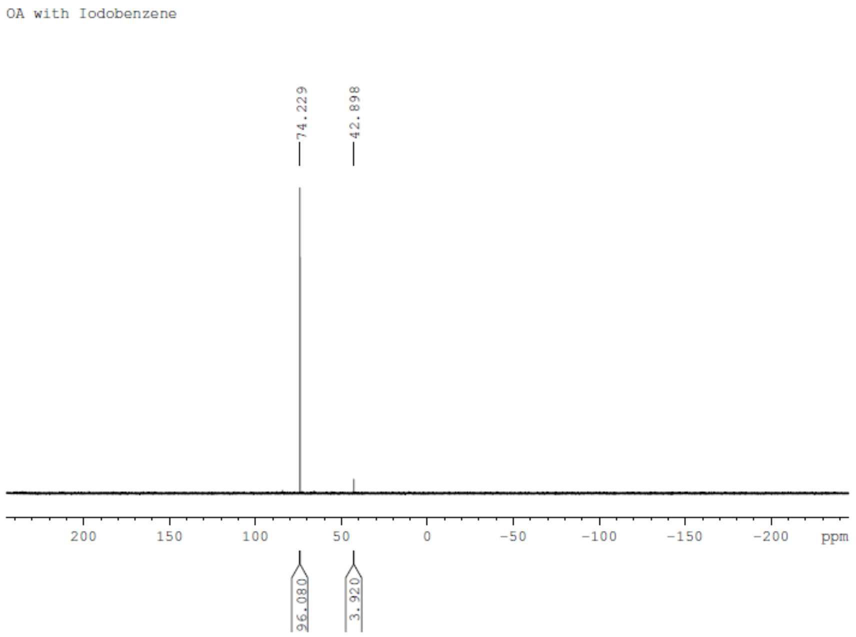

Supplementary Figure 4. $^{31}P\{^{1}H\}$ NMR spectrum of (2) in CD_2Cl_2

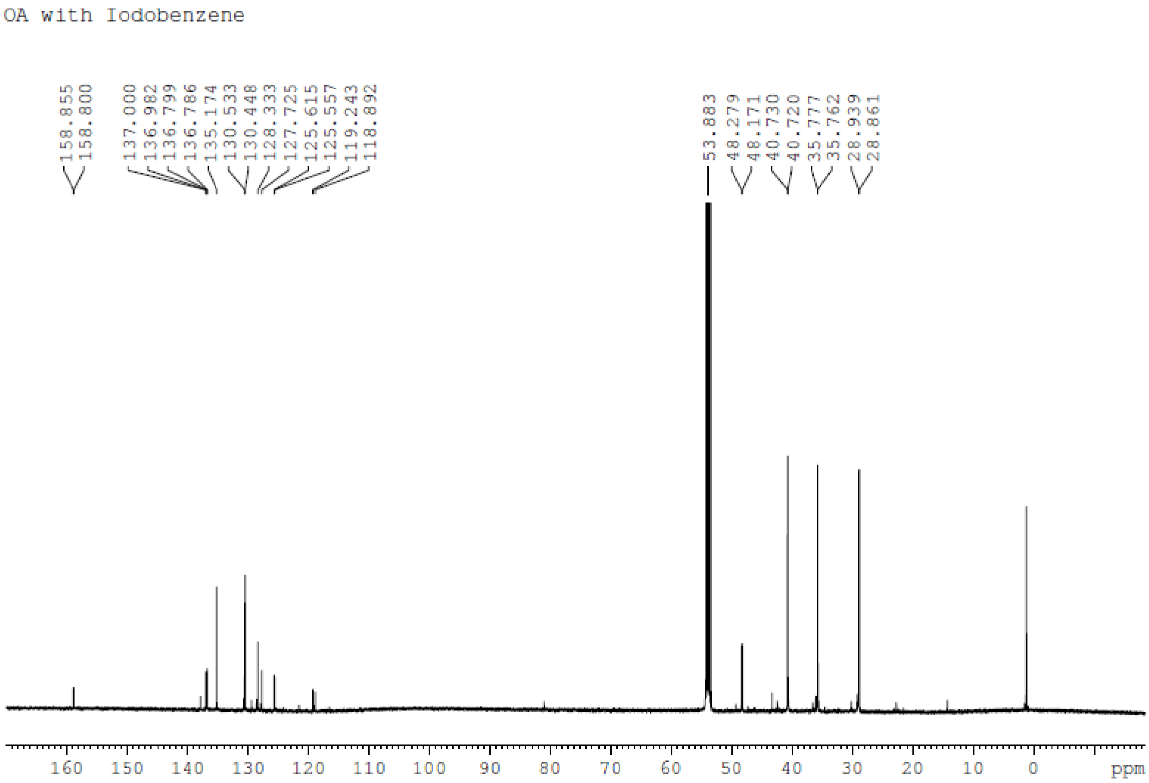

Supplementary Figure 5. $^{13}C\{^{1}H\}$ **NMR spectrum of (2) in** CD_2Cl_2

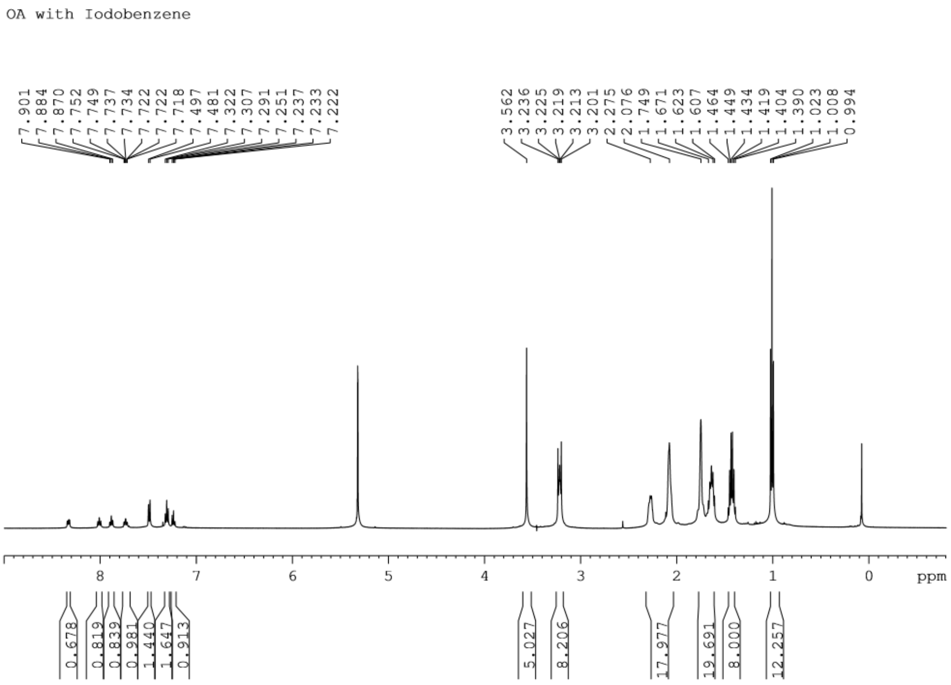

Supplementary Figure 6. ^{1}H **NMR spectrum of (2') in** CD_2Cl_2

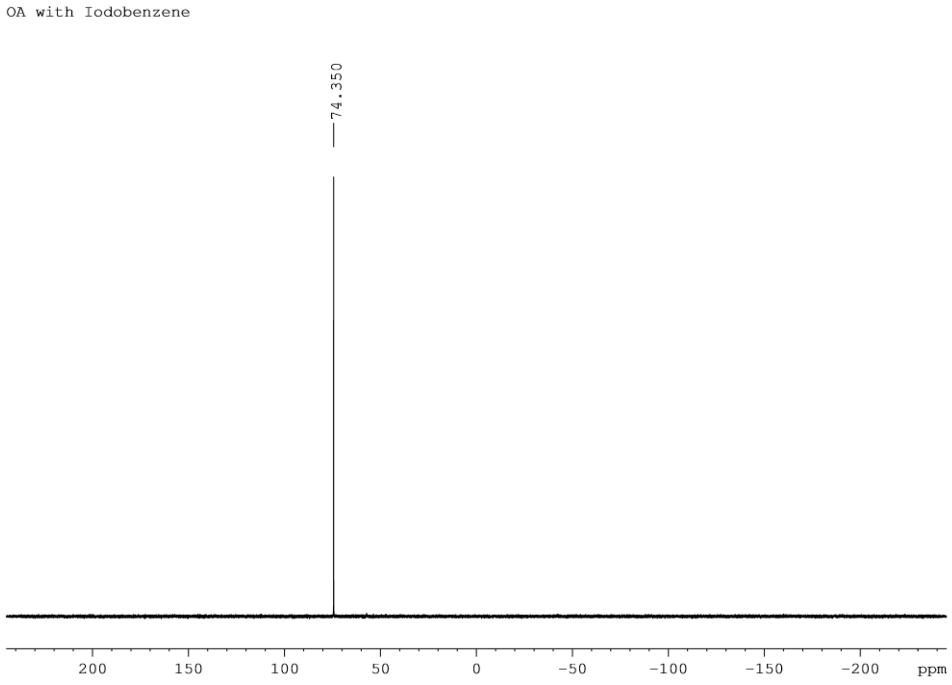

Supplementary Figure 7. $^{31}P\{^{1}H\}$ NMR spectrum of (2’) in CD_2Cl_2

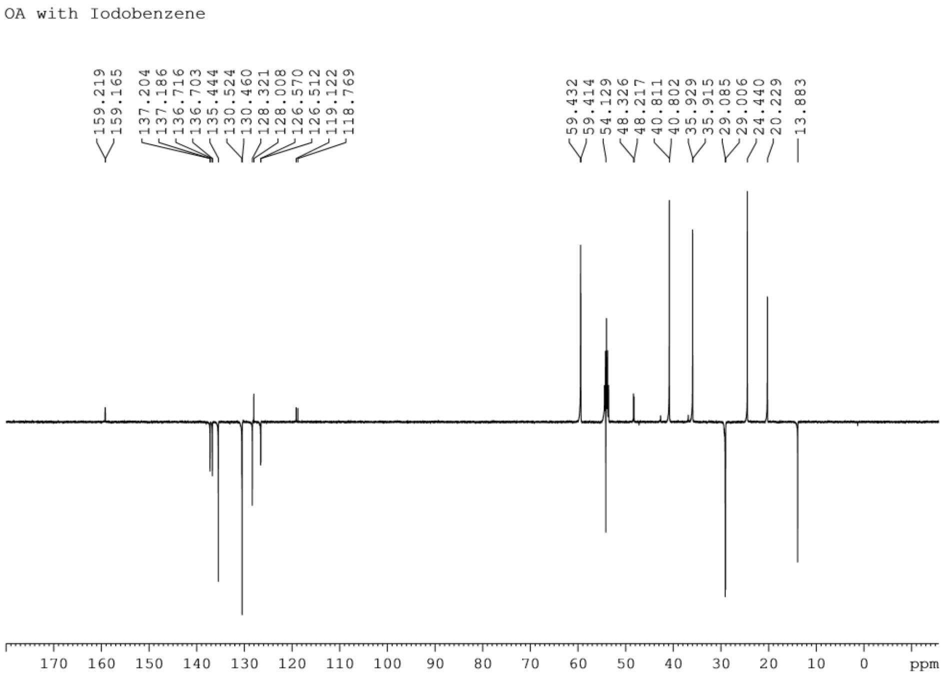

Supplementary Figure 8. $^{13}C\{^{1}H\}$ NMR spectrum of (2’) in CD_2Cl_2

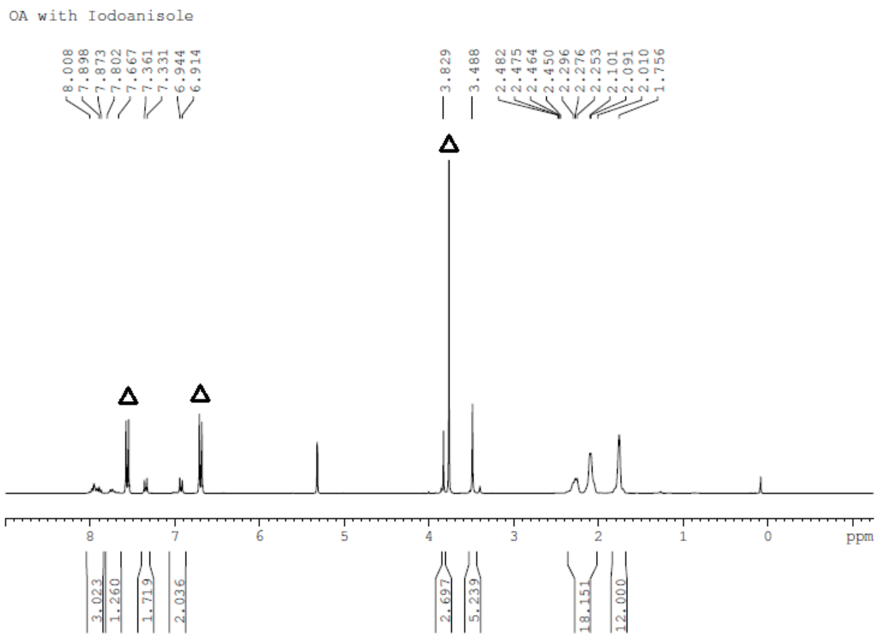

Supplementary Figure 9. ^{1}H NMR spectrum of (3) in CD_2Cl_2. (Black triangles represent the excess of Ar-I).

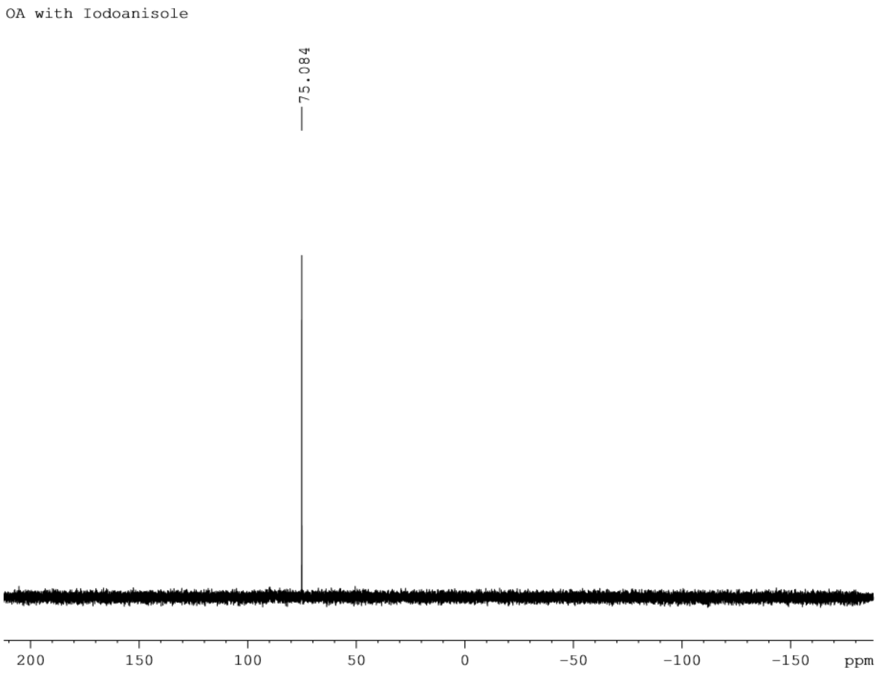

Supplementary Figure 10. **$^{31}P\{^{1}H\}$ NMR spectrum of (3) in CD_2Cl_2**

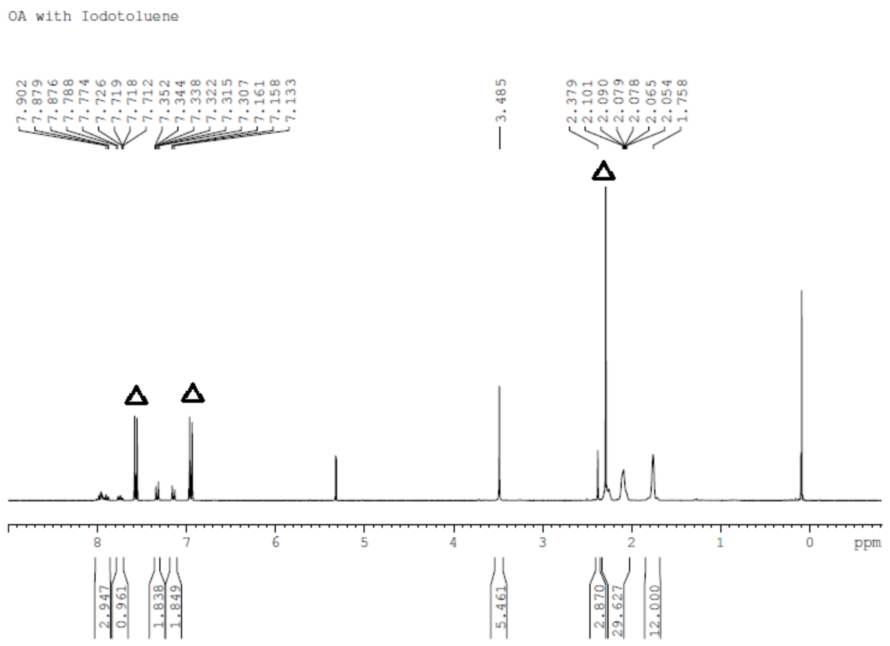

Supplementary Figure 11. ^{1}H NMR spectrum of (4) in CD_2Cl_2 (Black triangles represent the excess of Ar-I)

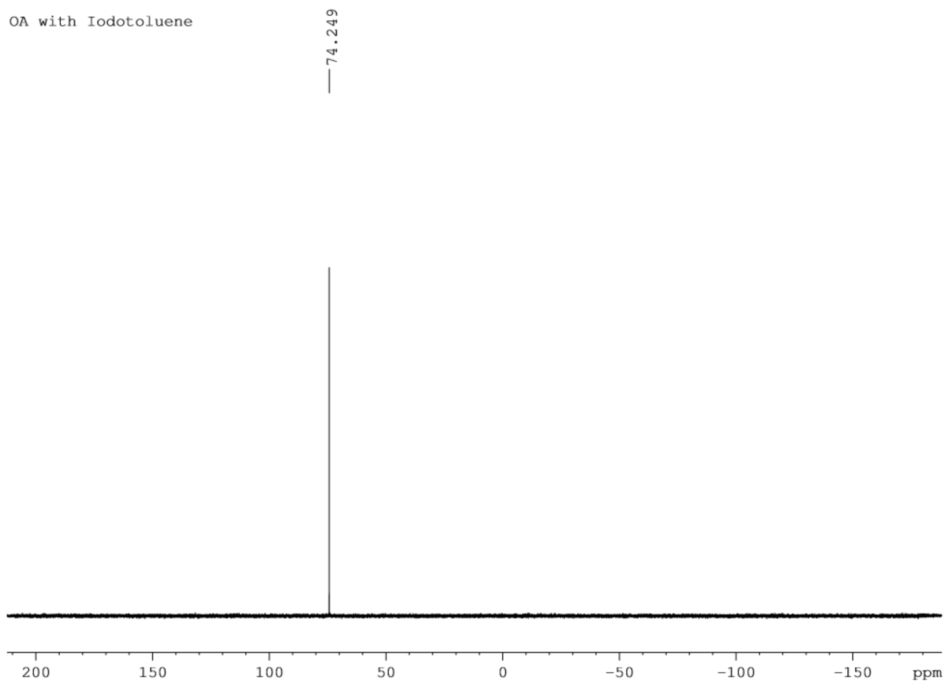

Supplementary Figure 12. ^{31}P{^{1}H} NMR spectrum of (4) in CD_2Cl_2

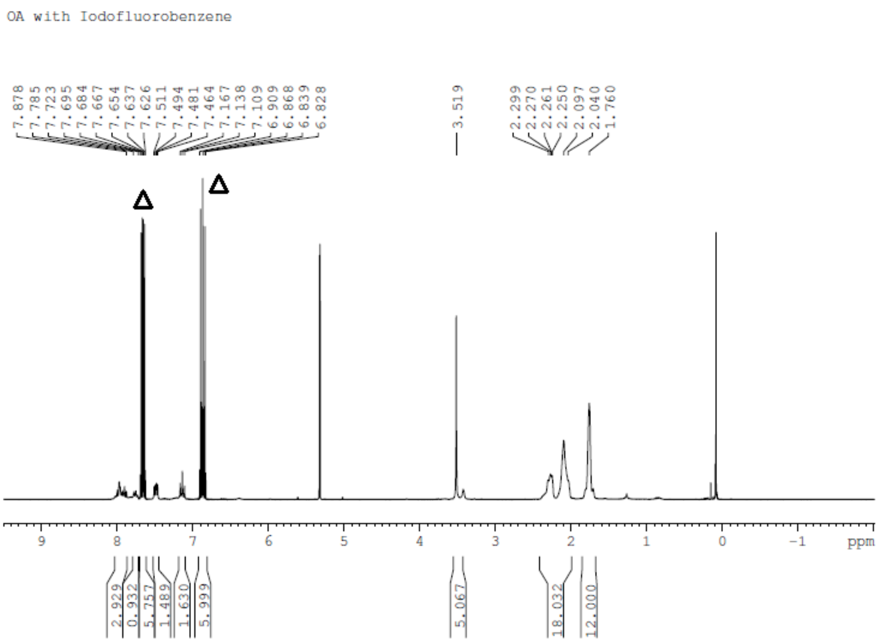

Supplementary Figure 13. ^{1}H NMR spectrum of (5) in CD_2Cl_2 (Δ: excess of Ar-I).

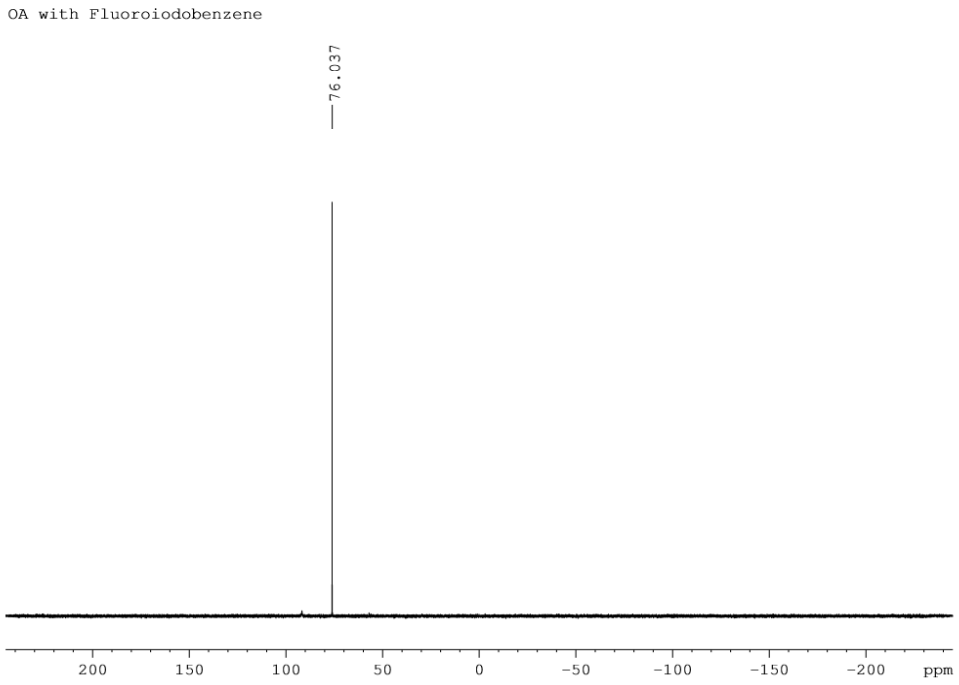

Supplementary Figure 14. ^{31}P{^{1}H} NMR spectrum of (5) in CD_2Cl_2

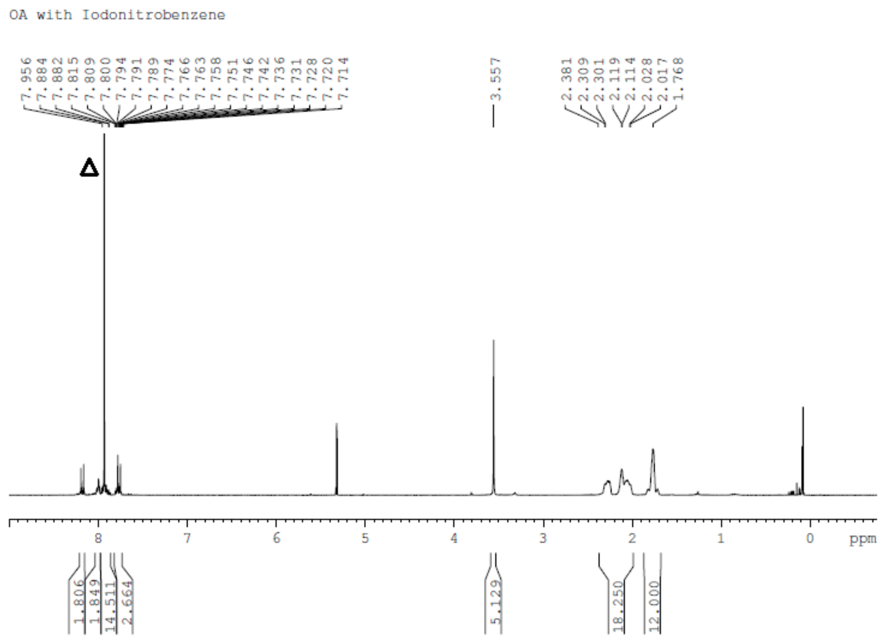

Supplementary Figure 15. ^{1}H NMR spectrum of (6) in CD_2Cl_2 (Δ: excess of Ar-I).

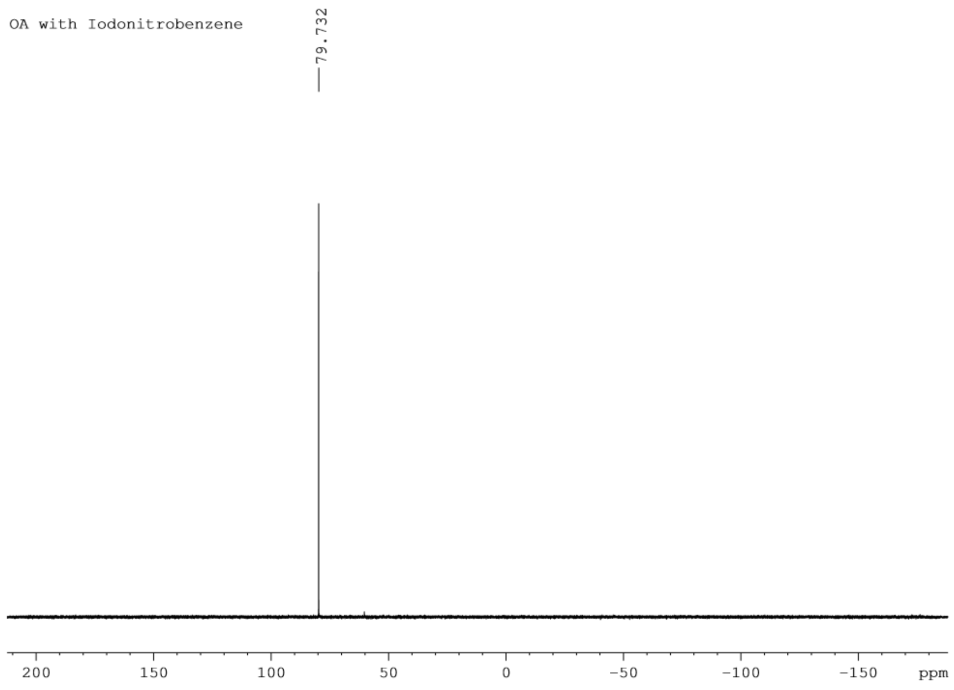

Supplementary Figure 16. $^{31}P\{^{1}H\}$ NMR spectrum of (6) in CD_2Cl_2

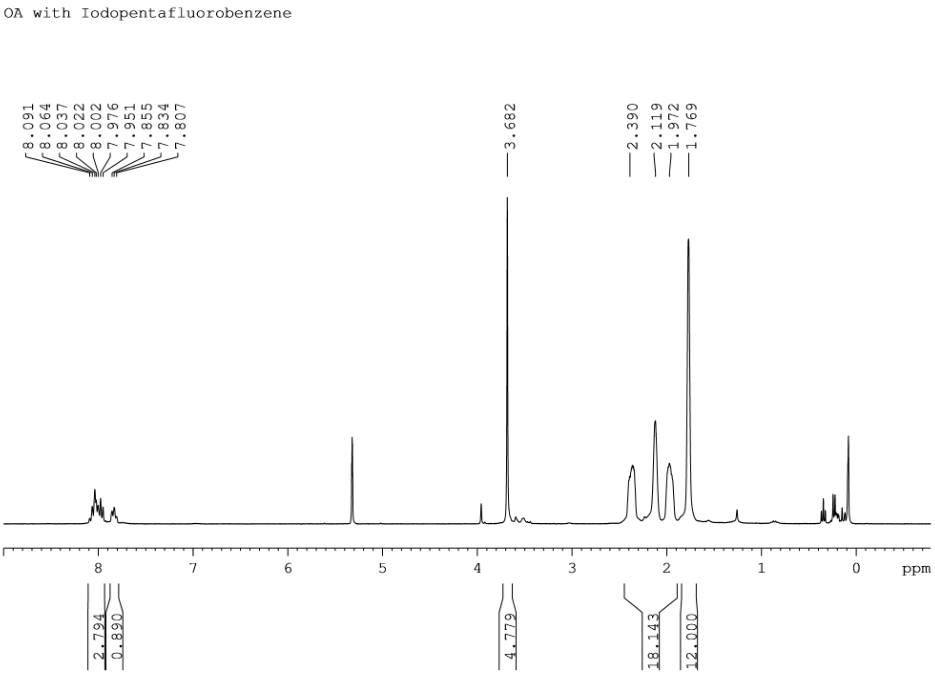

Supplementary Figure 17. ^{1}H NMR spectrum of (7) in CD_2Cl_2

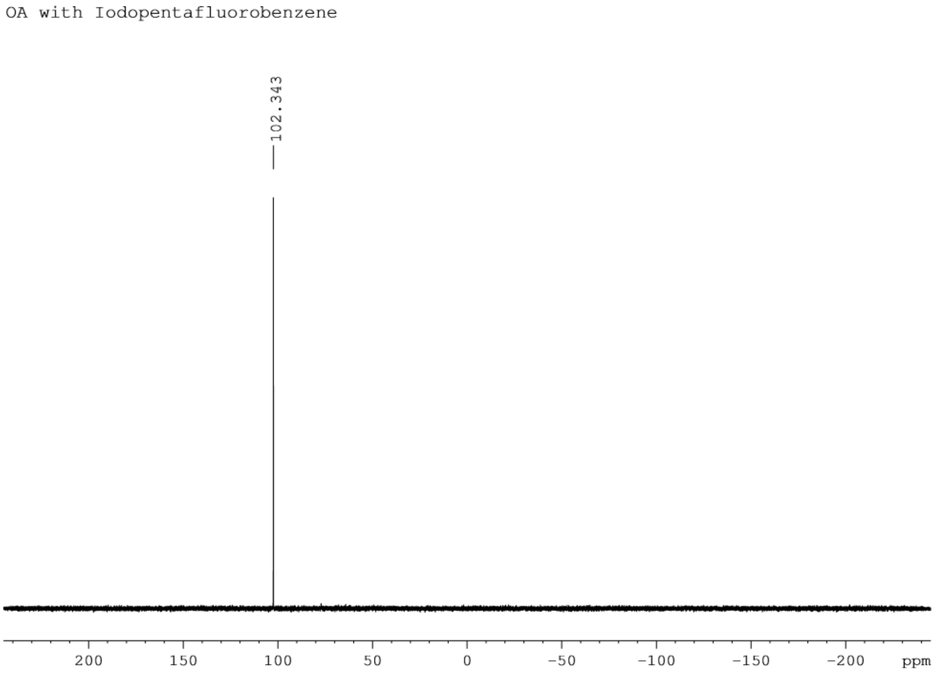

Supplementary Figure 18. $^{31}P\{^1H\}$ NMR spectrum of (7) in CD_2Cl_2

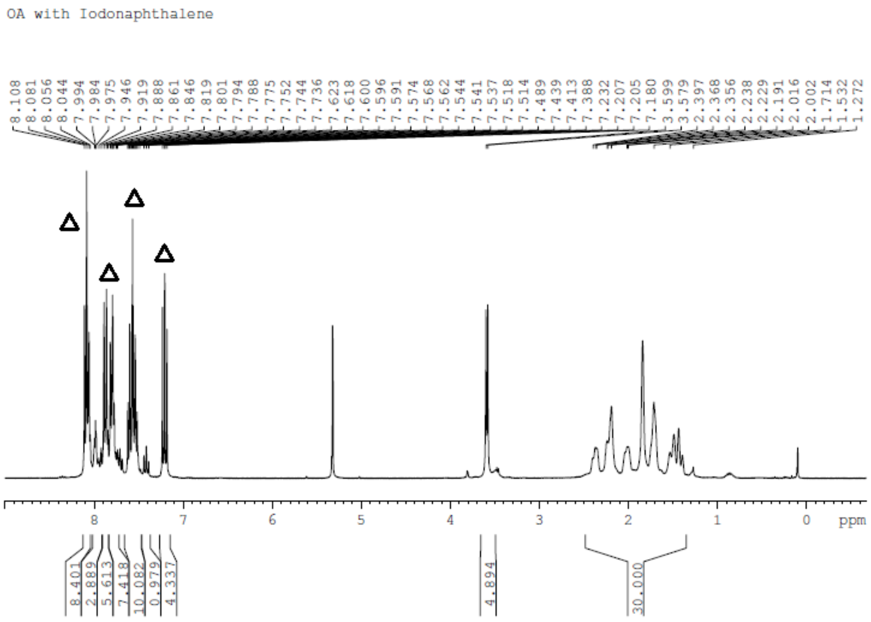

Supplementary Figure 19. ^{1}H NMR spectrum of (8) in CD_2Cl_2 ((Δ: excess of Ar-I).

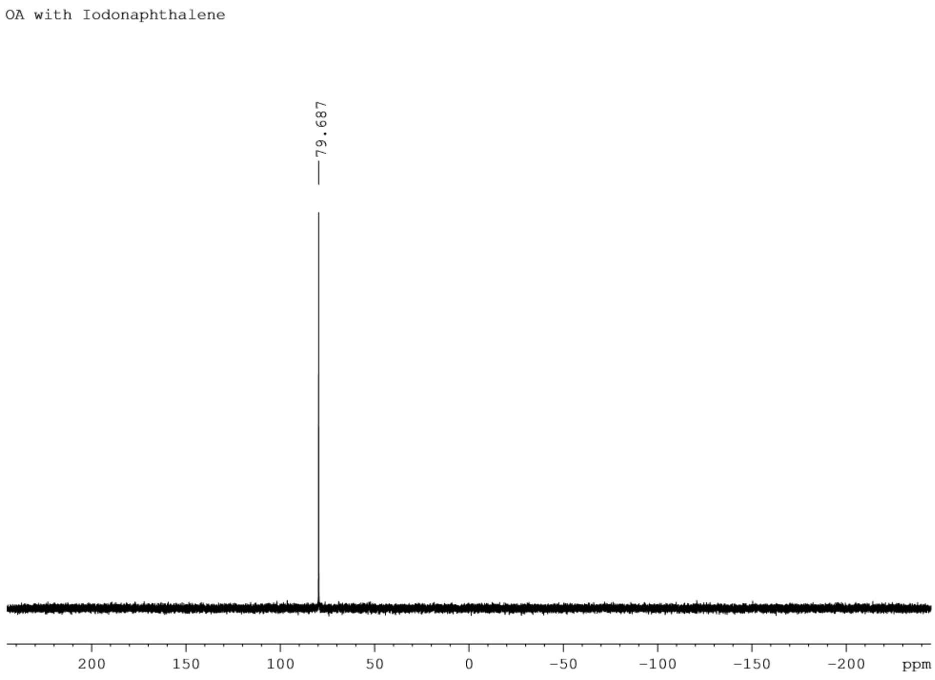

Supplementary Figure 20. $^{31}P\{^{1}H\}$ NMR spectrum of (8) in CD_2Cl_2

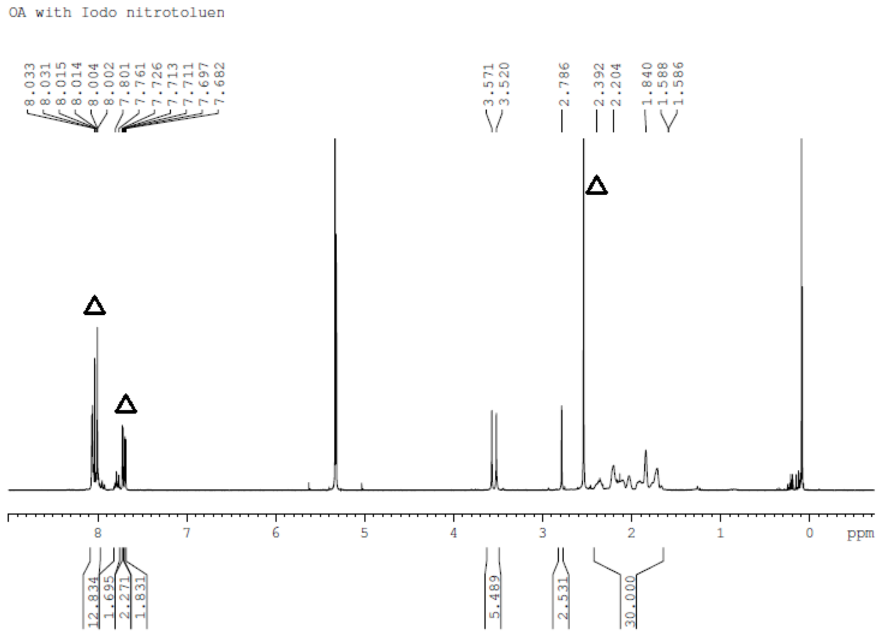

Supplementary Figure 21. ^{1}**H NMR spectrum of (9) in** CD_2Cl_2 **(Δ: excess of Ar-I).**

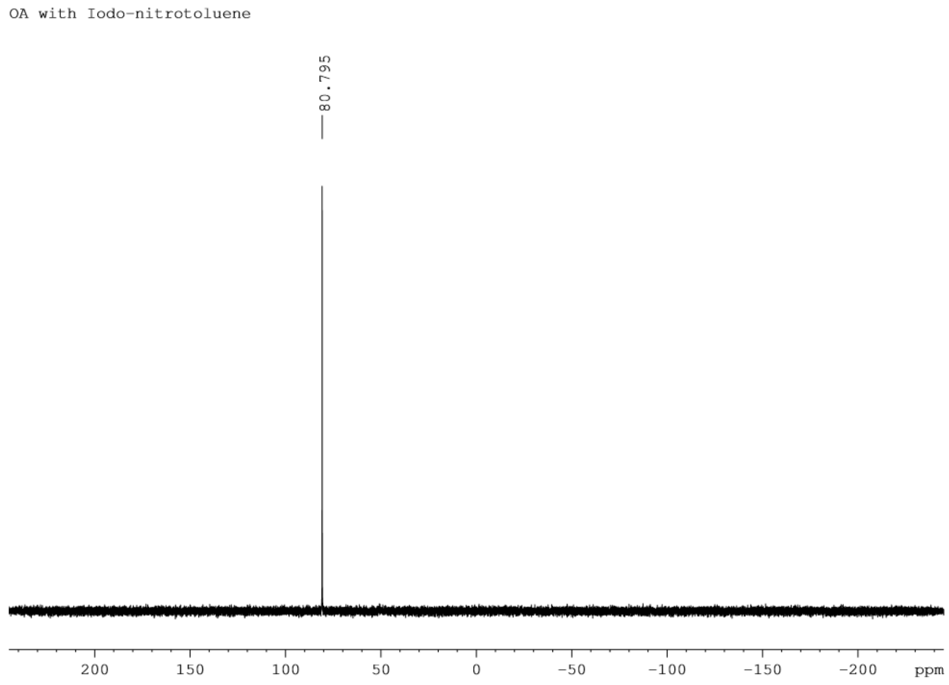

Supplementary Figure 22. $^{31}P\{^{1}H\}$ **NMR spectrum of (9) in** CD_2Cl_2

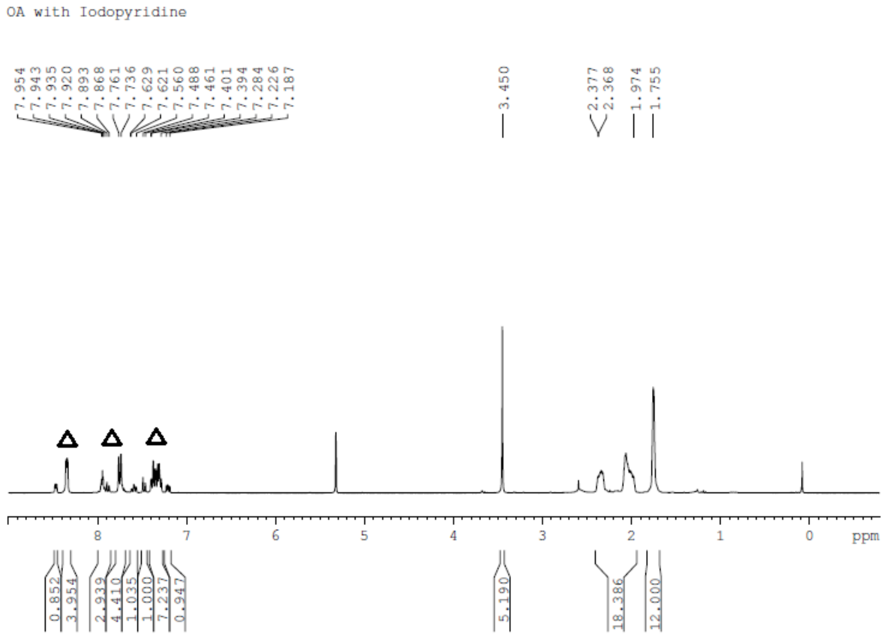

Supplementary Figure 23. ^{1}H NMR spectrum of (10) in CD_2Cl_2 (Δ: excess of Ar-I).

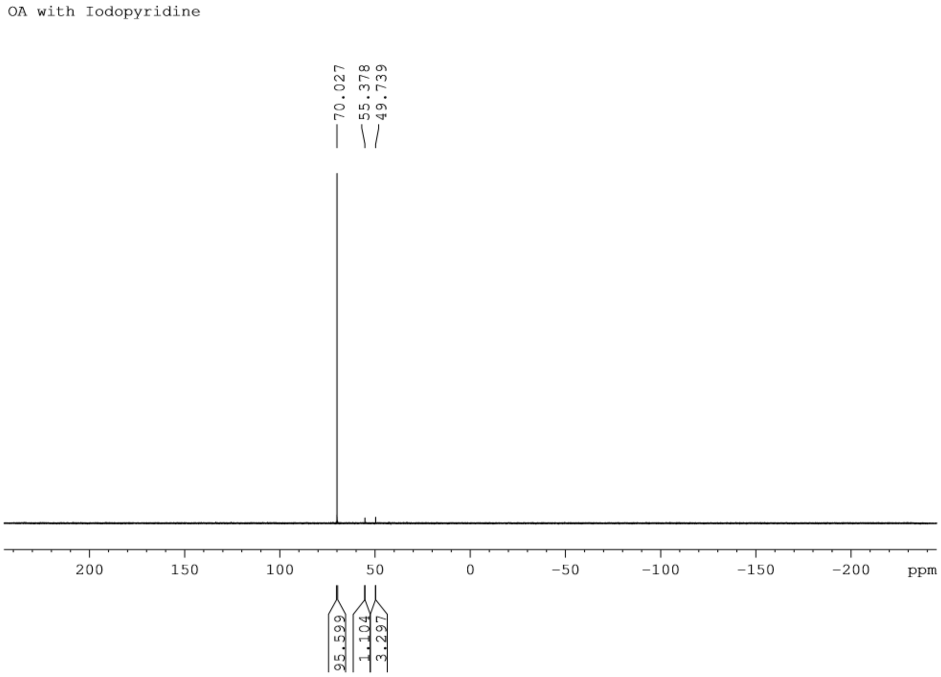

Supplementary Figure 24. $^{31}P\{^{1}H\}$ NMR spectrum of (10) in CD_2Cl_2

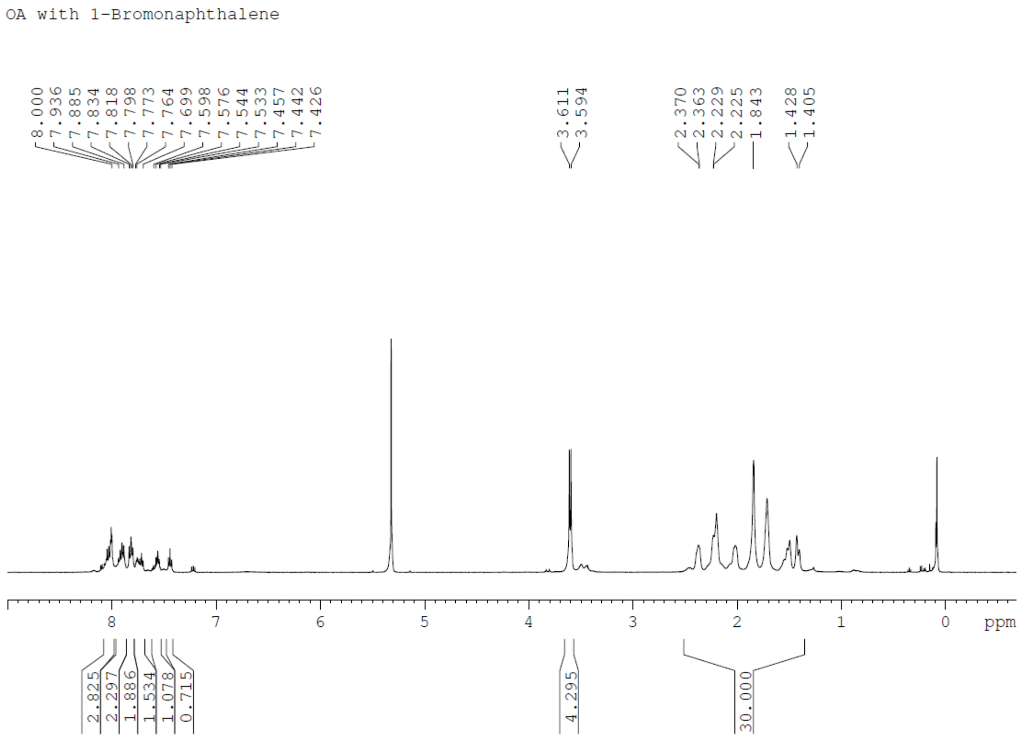

Supplementary Figure 25. ^{1}H NMR spectrum of (11) in CD_2Cl_2

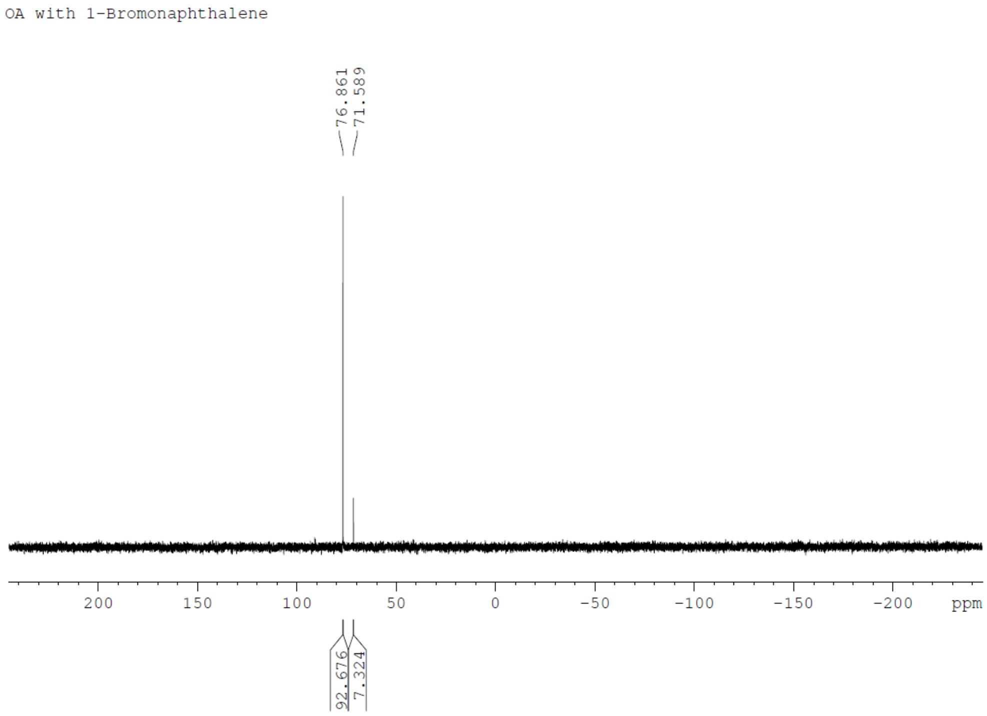

Supplementary Figure 26. $^{31}P\{^{1}H\}$ NMR spectrum of (11) in CD_2Cl_2

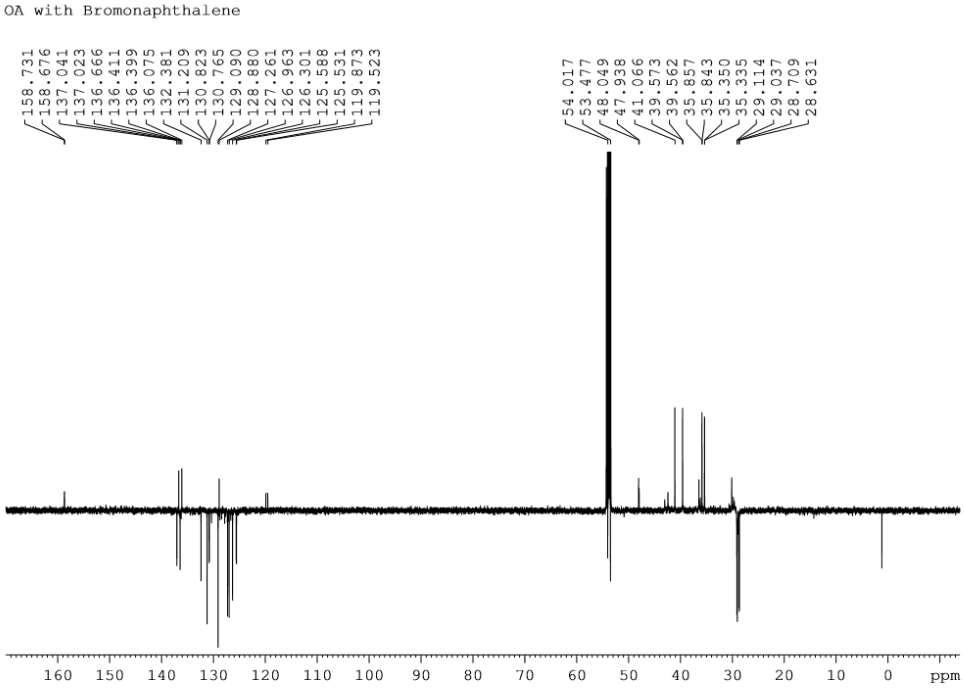

Supplementary Figure 27. $^{13}C\{^{1}H\}$ NMR spectrum of (11) in CD_2Cl_2

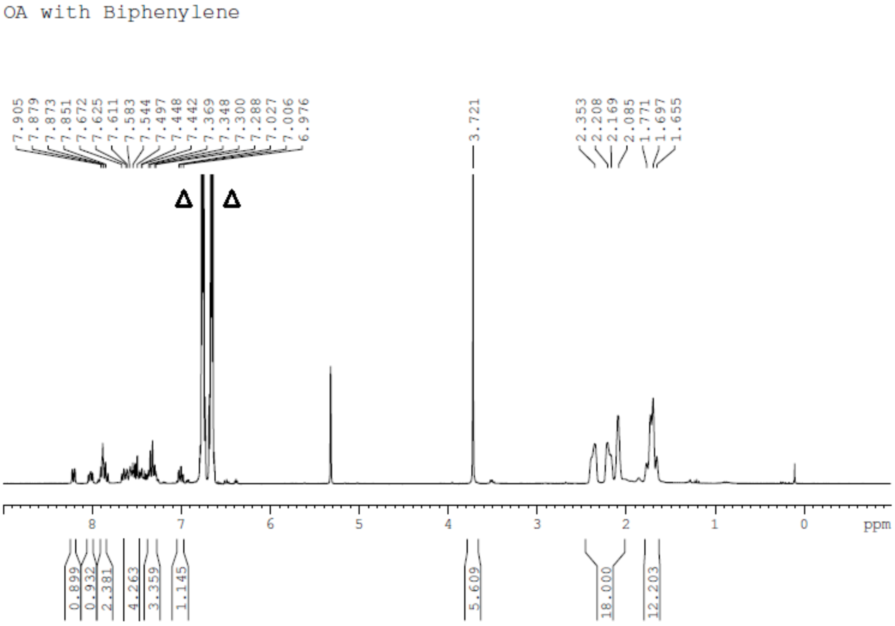

Supplementary Figure 28. ^{1}H NMR spectrum of (12) in CD_2Cl_2 (Δ: excess of biphenylene).

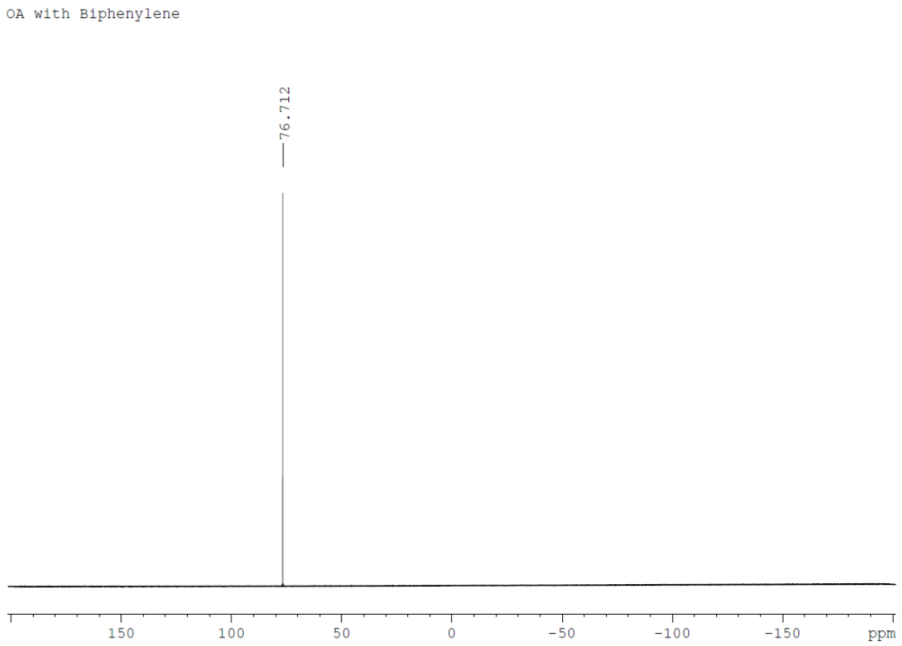

Supplementary Figure 29. $^{31}P\{^1H\}$ NMR spectrum of (12) in CD_2Cl_2

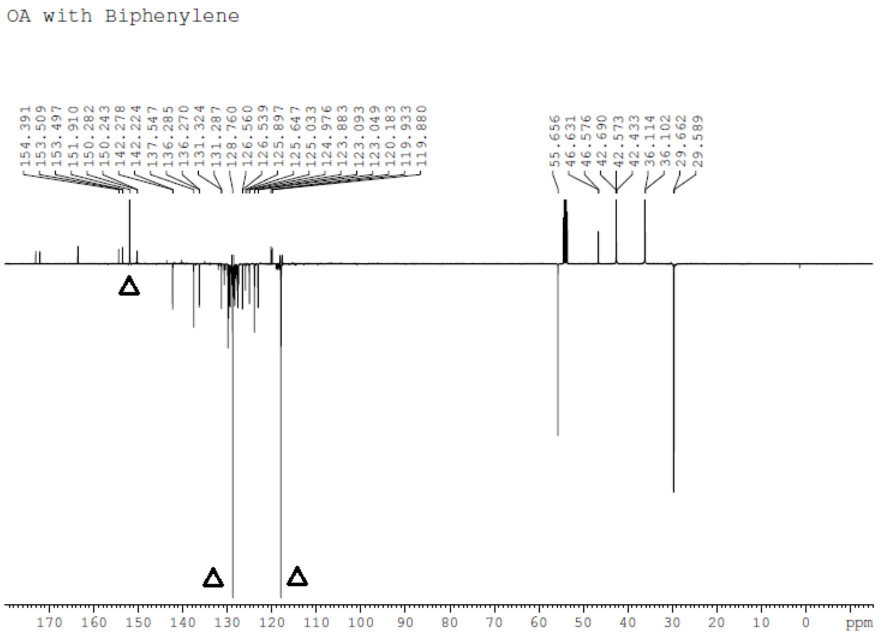

Supplementary Figure 30. $^{13}C\{^1H\}$ NMR spectrum of (12) in CD_2Cl_2 (Δ: excess biphenylene).

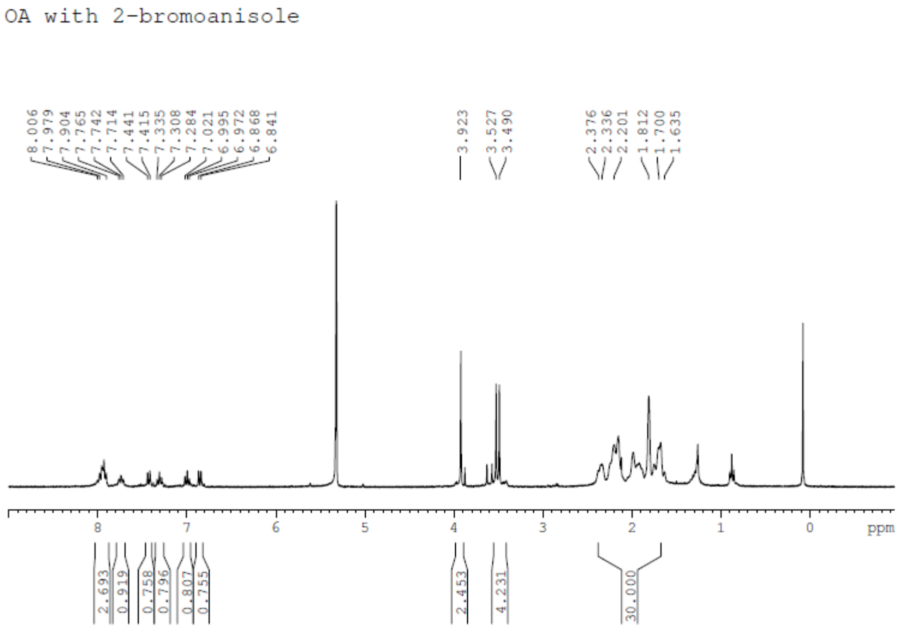

Supplementary Figure 31. **^{1}H NMR spectrum of (13) in CD_2Cl_2**

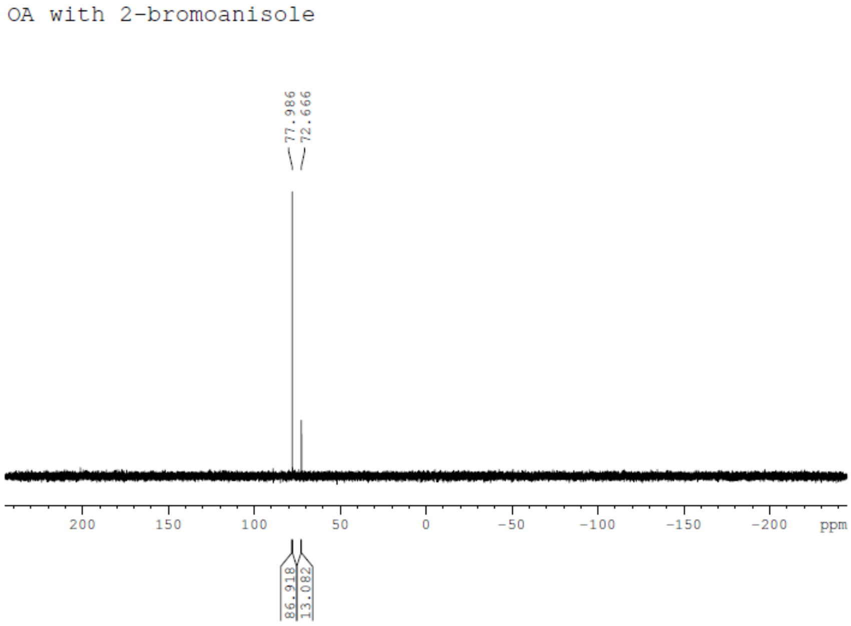

Supplementary Figure 32. **$^{31}P\{^{1}H\}$ NMR spectrum of (13) in CD_2Cl_2**

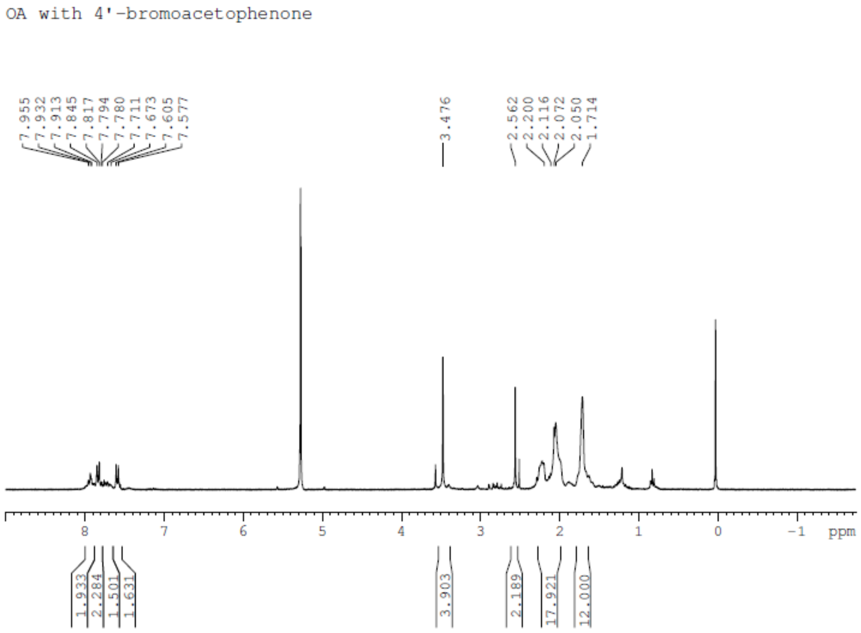

Supplementary Figure 33. ^{1}H NMR spectrum of (14) in CD_2Cl_2

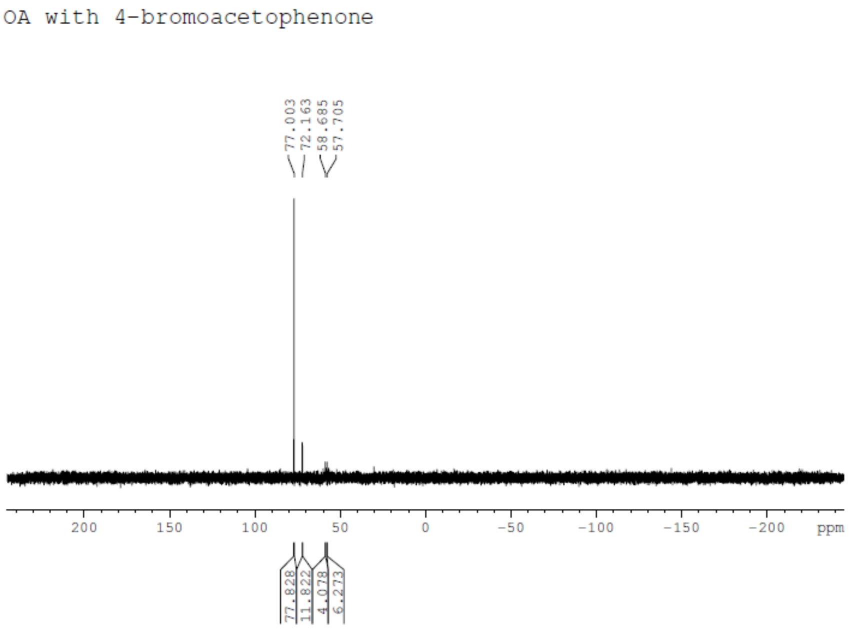

Supplementary Figure 34. $^{31}P\{^{1}H\}$ NMR spectrum of (14) in CD_2Cl_2

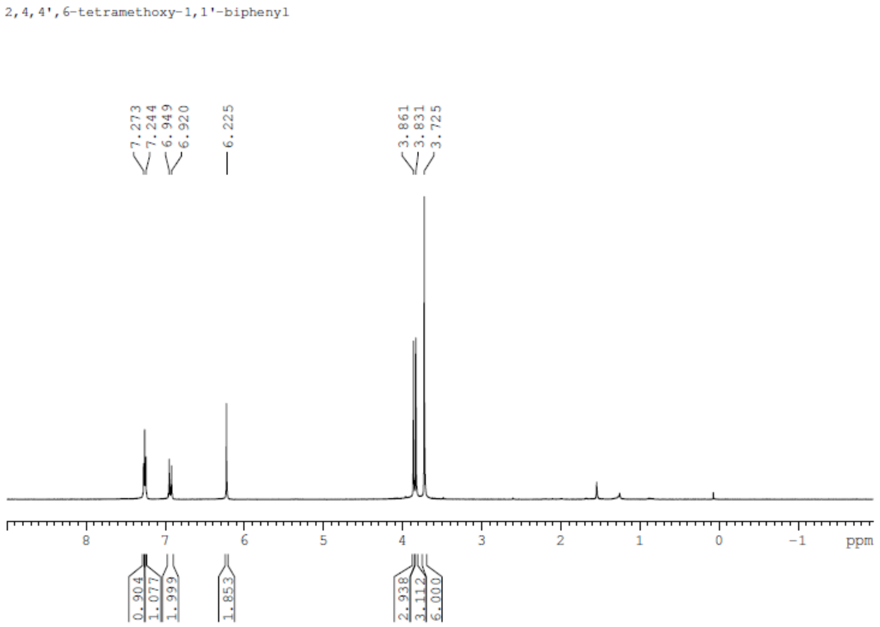

Supplementary Figure 35. ^{1}H NMR spectrum of (15) in $CDCl_3$

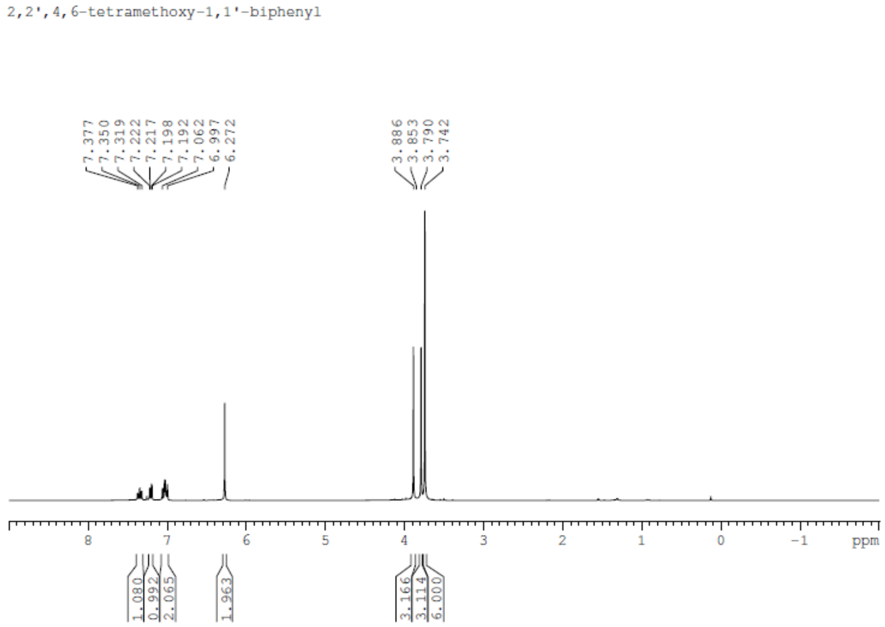

Supplementary Figure 36. ^{1}H NMR spectrum of (16) in $CDCl_3$

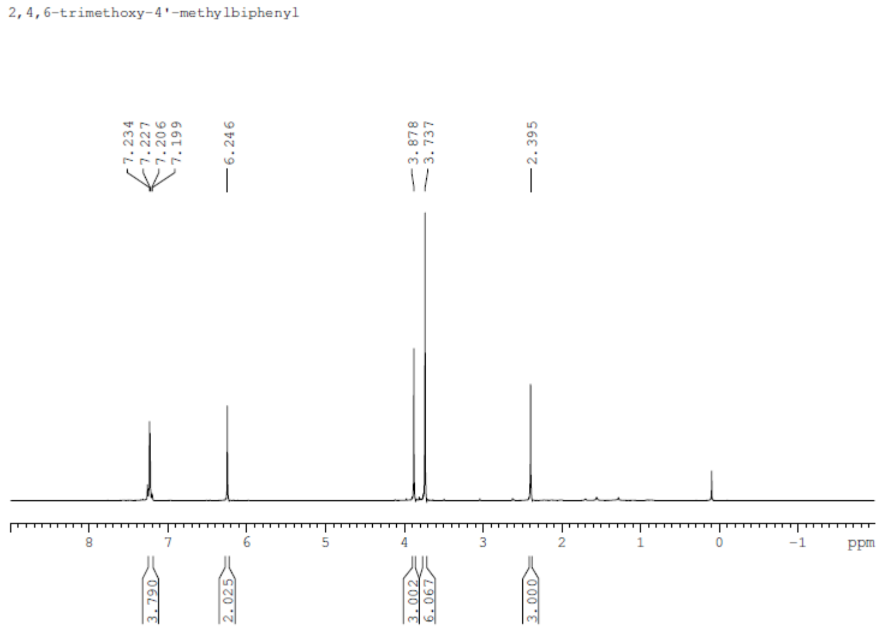

Supplementary Figure 37. ^{1}H NMR spectrum of (17) in $CDCl_3$

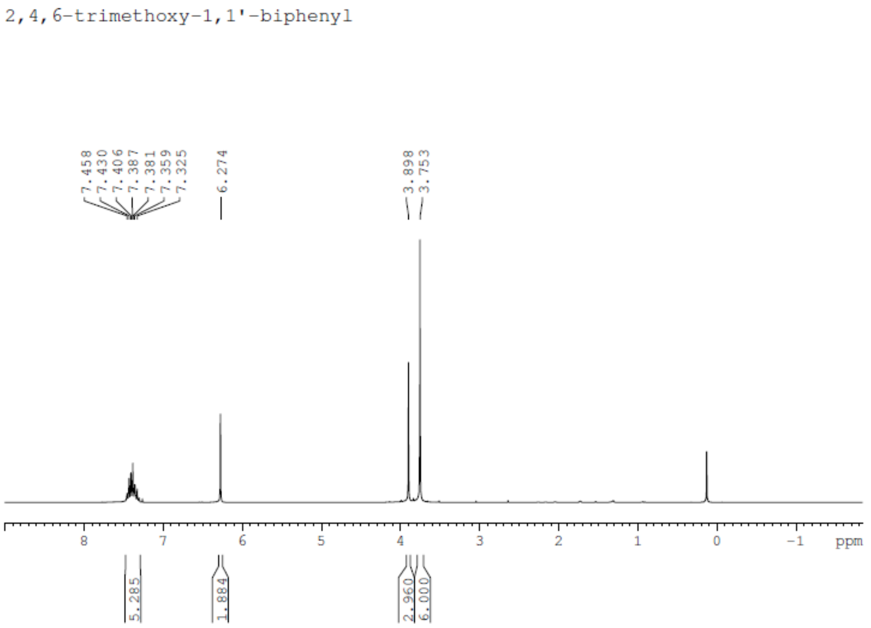

Supplementary Figure 38. ^{1}H NMR spectrum of (18) in $CDCl_3$

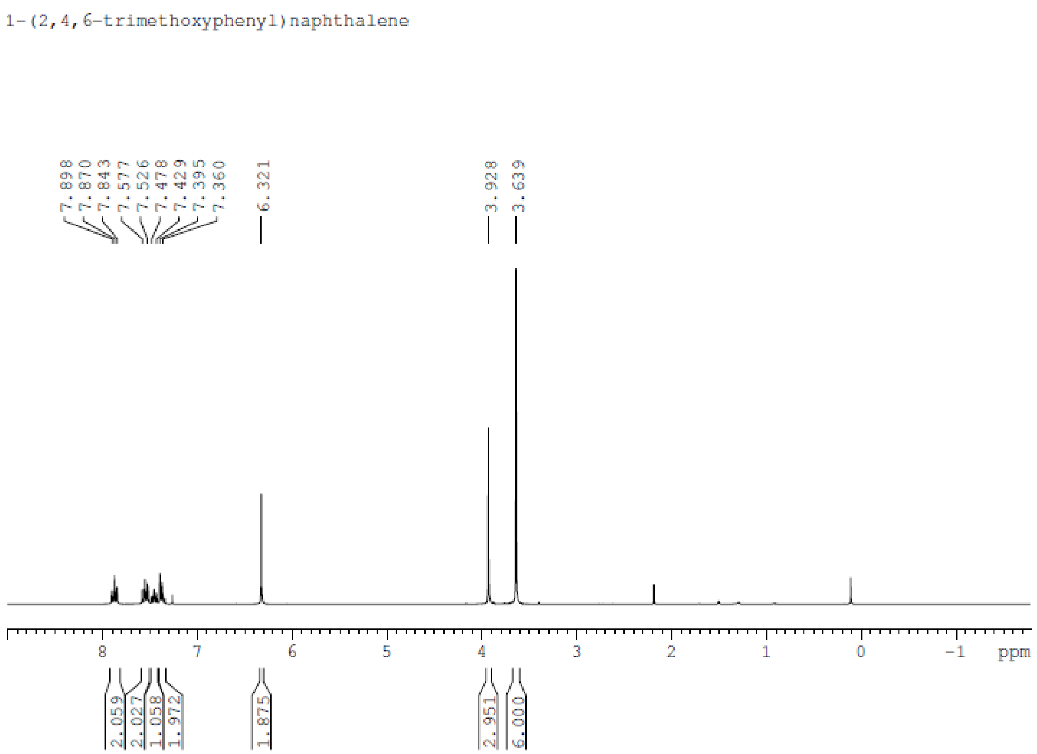

Supplementary Figure 39. ^{1}H NMR spectrum of (19) in $CDCl_3$.

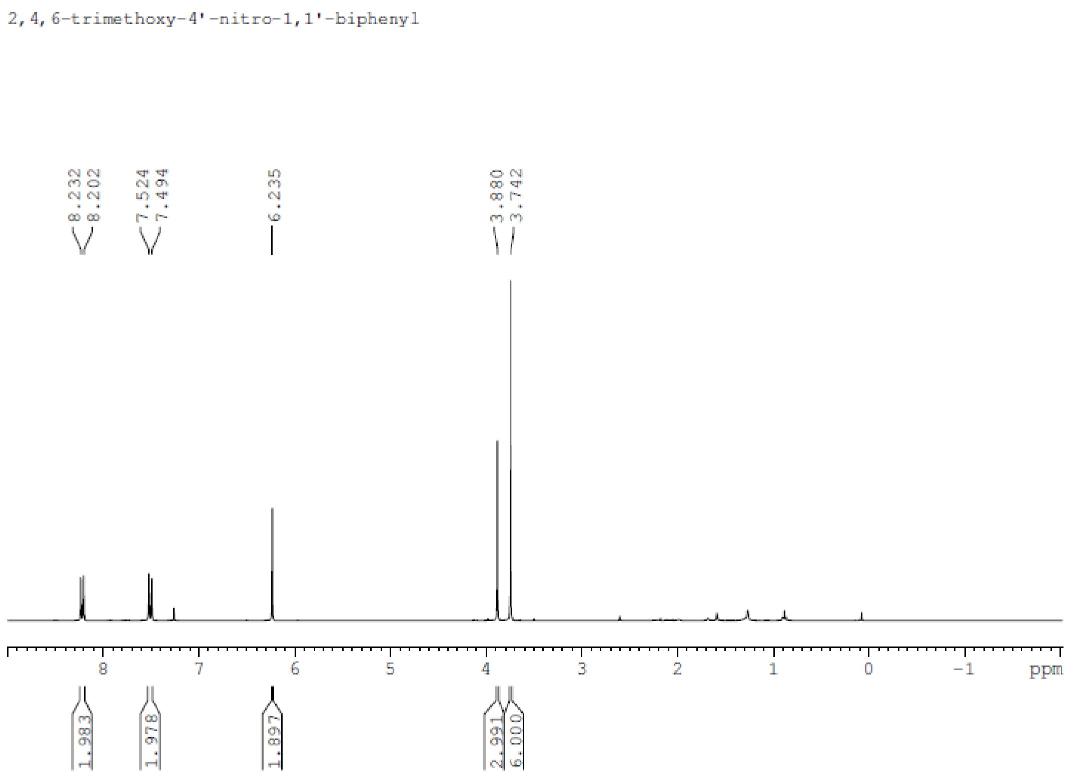

Supplementary Figure 40. ^{1}H NMR spectrum of (20) in $CDCl_3$

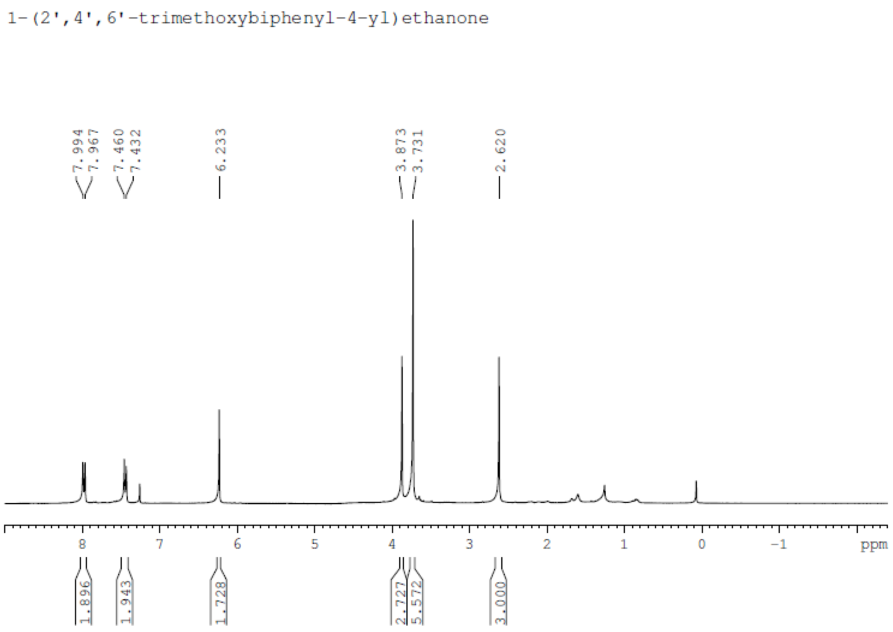

Supplementary Figure 41. ^{1}H NMR spectrum of (21) in $CDCl_3$

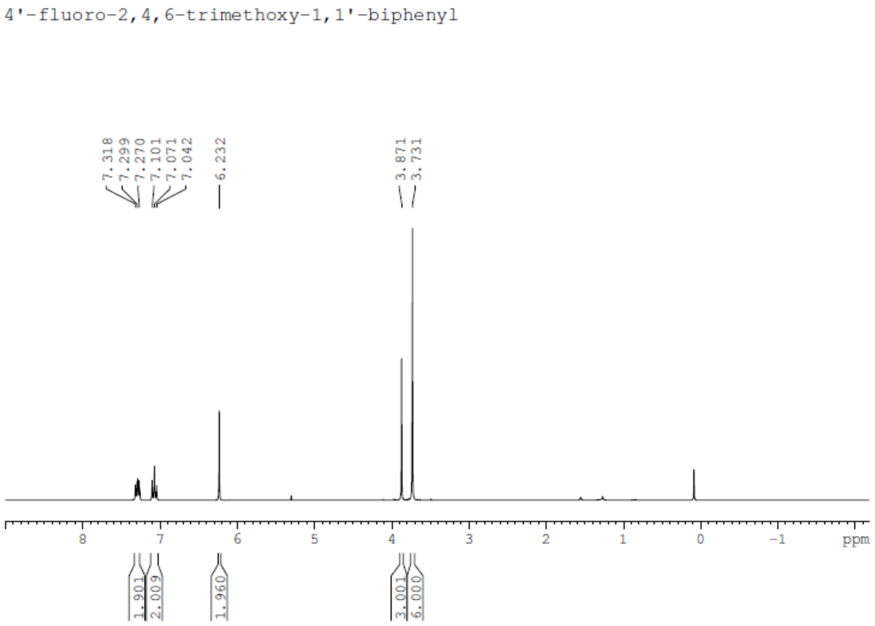

Supplementary Figure 42. ^{1}H NMR spectrum of (22) in $CDCl_3$

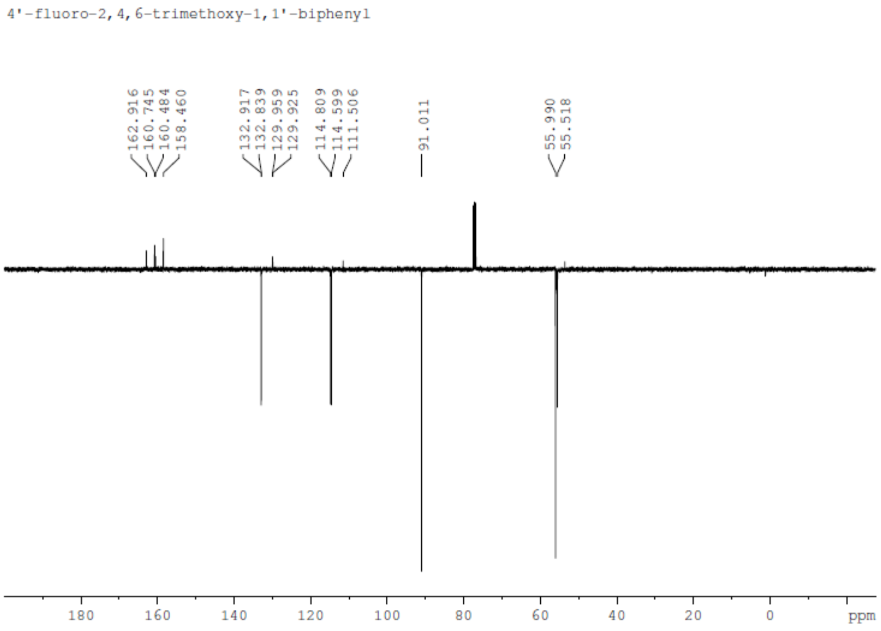

Supplementary Figure 43. $^{13}C\{^{1}H\}$ **NMR spectrum of (22) in** $CDCl_3$

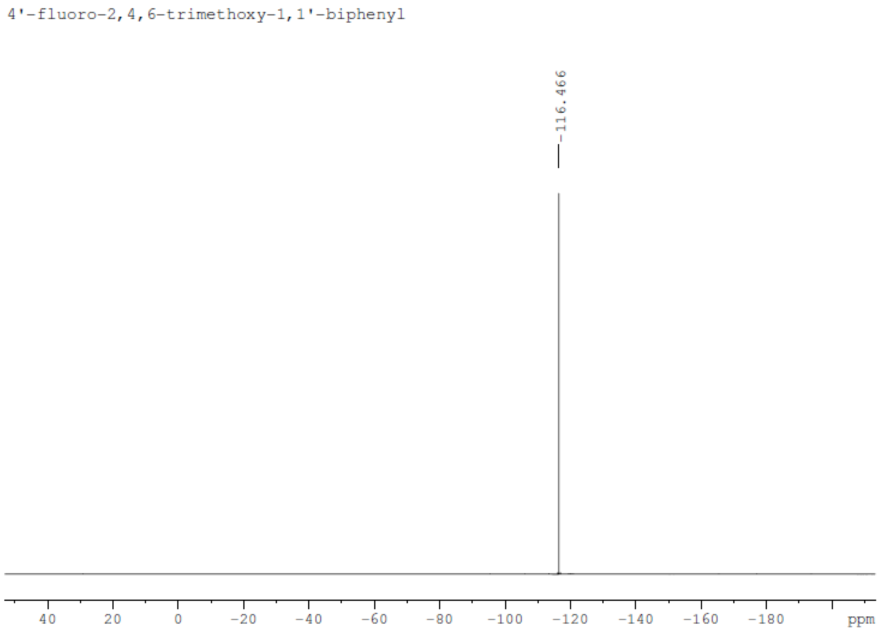

Supplementary Figure 44. $^{19}F\{^{1}H\}$ **NMR spectrum of (22) in** $CDCl_3$**.**

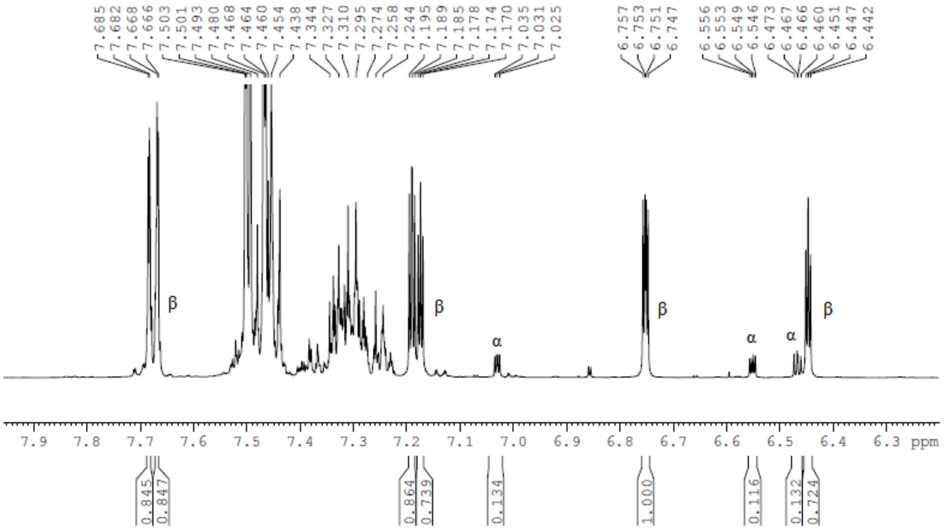

Supplementary Figure 45. ^{1}H NMR spectrum of the coupling product between iodobenzene and 1-phenylpyrrole in $CDCl_3$.

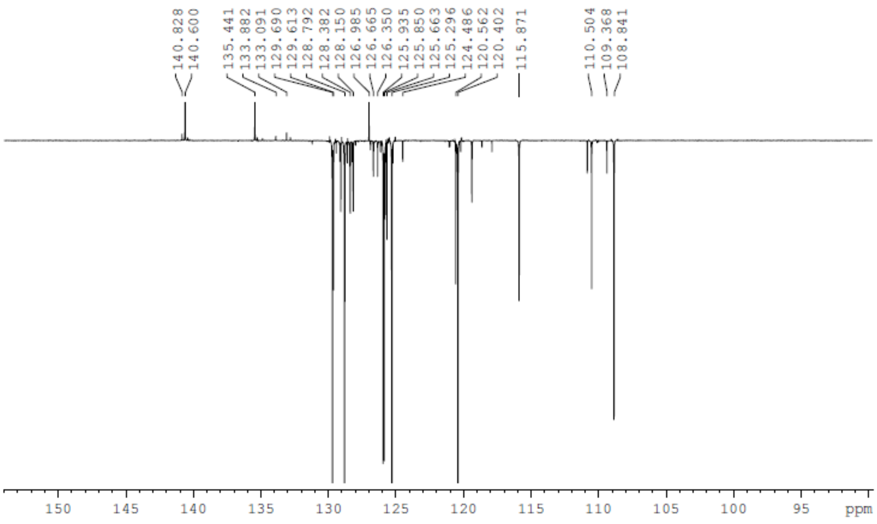

Supplementary Figure 46. $^{13}C\{^{1}H\}$ NMR spectrum of the coupling product between iodobenzene and 1-phenylpyrrole in $CDCl_3$.

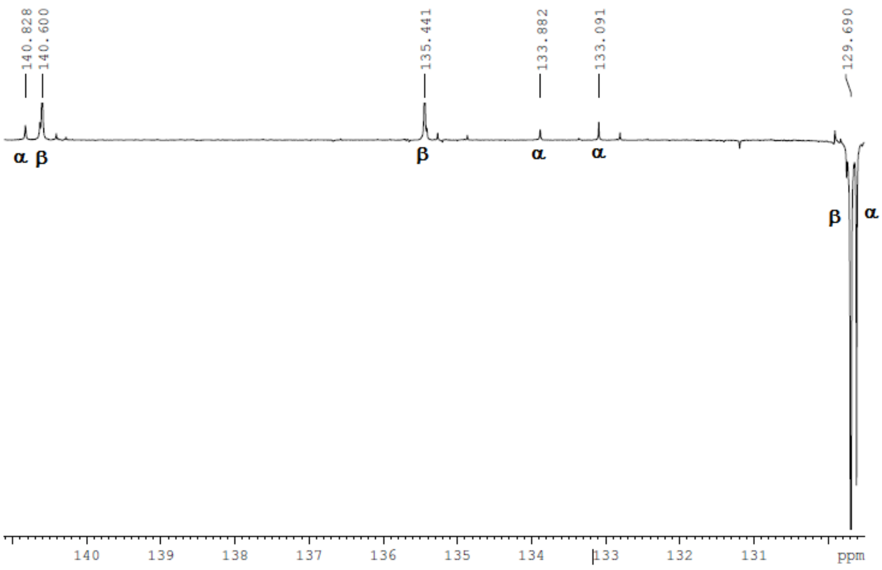

Supplementary Figure 47. $^{13}C\{^{1}H\}$ NMR spectrum of the coupling product between iodobenzene and 1-phenylpyrrole in $CDCl_3$ (zoom, 140-130 ppm).

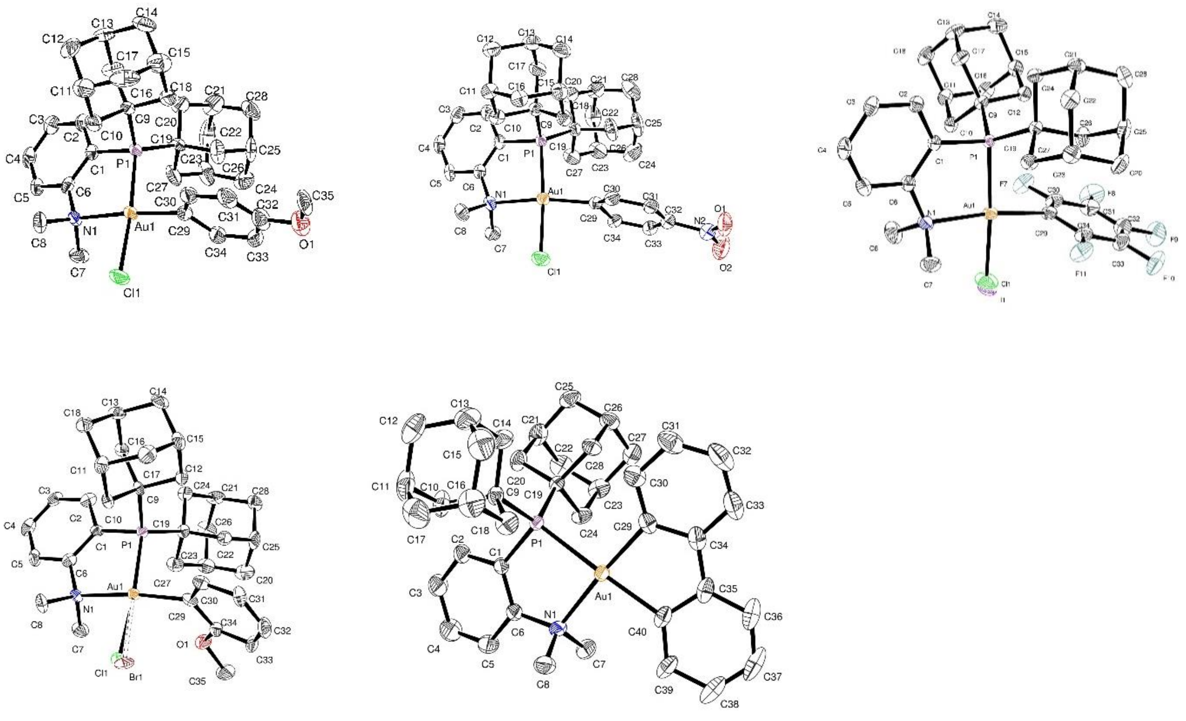

Supplementary Figure 48. Molecular Structures of complexes 3-12 determined by X-ray diffraction. Hydrogen atoms, counteranion and solvent molecules are omitted for clarity.

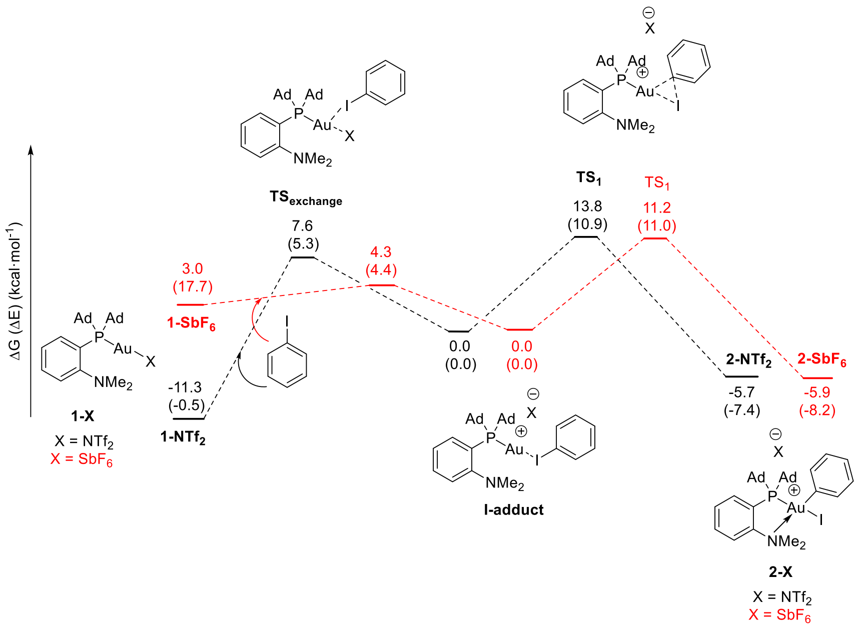

Supplementary Figure 49. Computed pathways for the oxidative addition of PhI to complex 1-NTf2 and 1-SbF6. Energy profiles computed in solvent (SMD model: Dichloromethane) at the B97D (SMD-DCM)/SDD+f(Au),SDD(I,Sb),6-31G** (other atoms) level of theory. Electronic energy (Δ*E*) including ZPE correction into brackets and Gibbs free energy (Δ*G*) in kcal·mol^{-1}. All energies referred to the I-adduct.

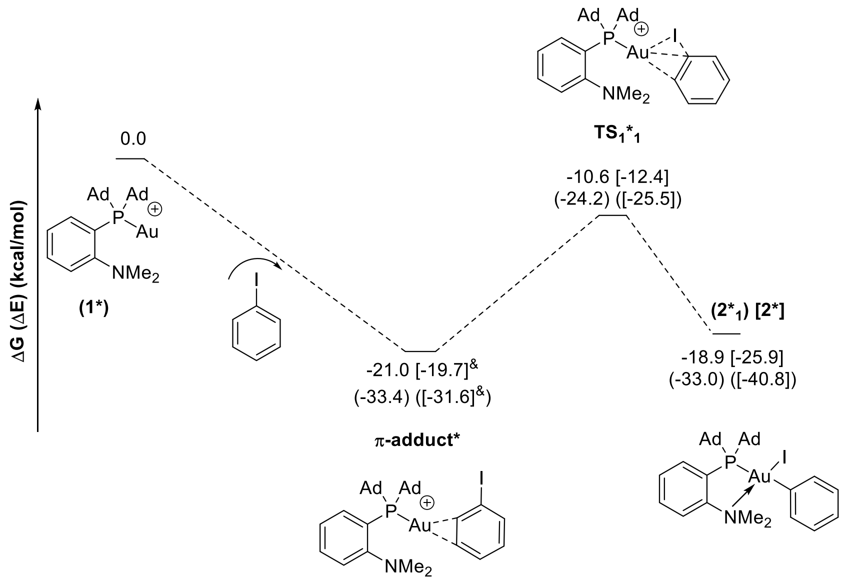

Supplementary Figure 50. Computed pathway for the oxidative addition of PhI to cationic complex 1* leading to the other stereosiomer 2*1. Energy profile computed in the gas phase at the B97D/SDD+f(Au),SDD(I),6-31G** (other atoms) level of theory. Into brackets [] are reported the energy values for the other isomer (I in *trans* to P, **2***) calculated in gas phase. In that case, **TS1*** is connected to I-adduct& rather than π-adduct.

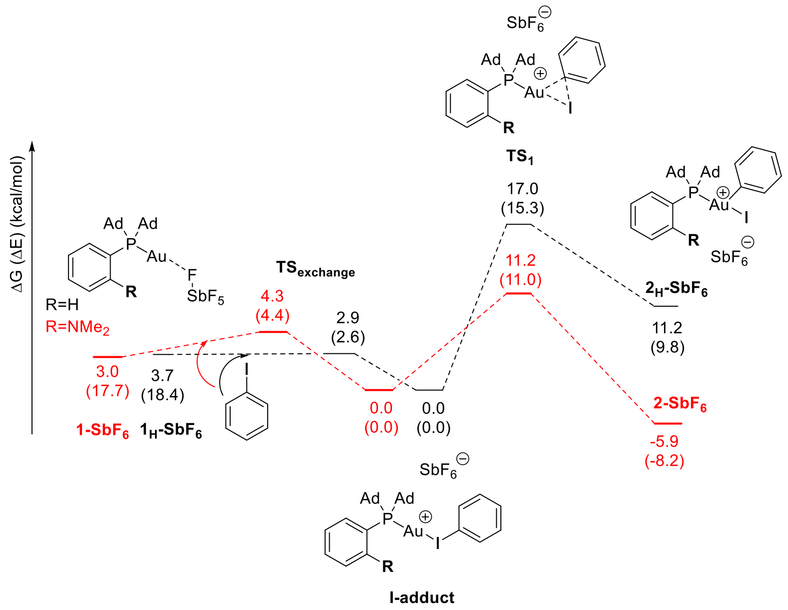

Supplementary Figure 51. Computed pathway for the oxidative addition of PhI to the model complex 1_H-SbF_6 devoid of NMe_2 group. Energy profile computed in solvent (DCM) at the B97D/SDD+f(Au),SDD(I,Sb),6-31G** (other atoms) level of theory. Energy profile computed at the same level of theory for **1-SbF_6** has been superposed for comparison (in red). Electronic energy (Δ*E*) including ZPE correction into brackets and Gibbs free energy (Δ*G*) in kcal·mol^{-1}.

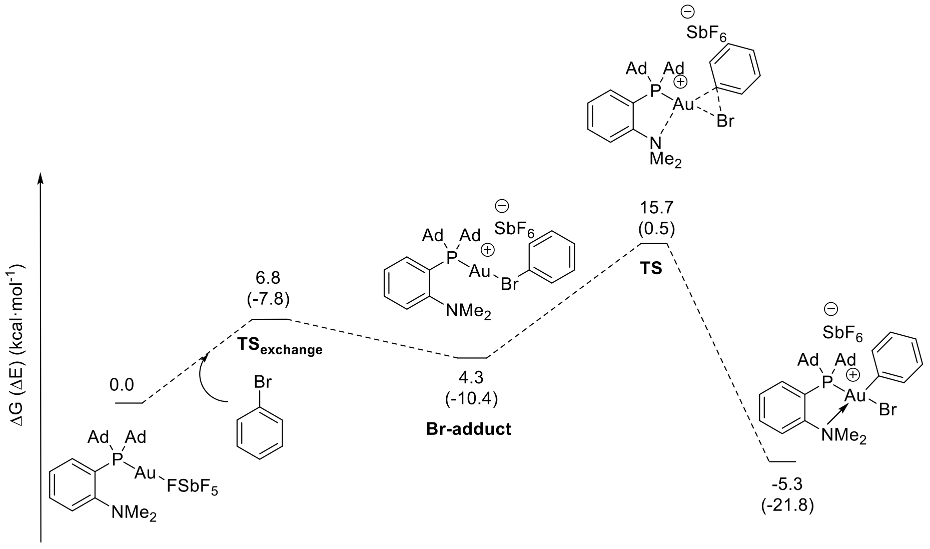

Supplementary Figure 52. Computed pathway for the oxidative addition of PhBr to complex 1-SbF_6. Reaction profile computed in solvent (SMD model: Dichloromethane) at the B97D(SMD-DCM)/SDD+f(Au),SDD(Br,Sb),6-31G** (other atoms) level of theory Electronic energy (Δ*E*) including ZPE correction into brackets and Gibbs free energy (Δ*G*) in kcal·mol^{-1}, in solution.

Supplementary Tables

Supplementary Table 1. Screening of halide scavengers and solvents.*

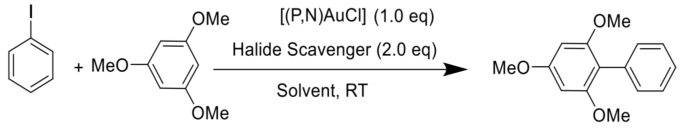

Entry	Halide Scavenger	Solvent	Yield (%)#
1	$AgBF_4$	DCM	10%
2	Ag_2O	DCM	0%
3	AgCSA	DCM	0%
4	AgCSA	DMF	0%
5	AgCSA	DMSO	0%
6	$AgSbF_6$	TFA	0%
7	$AgSbF_6$	DCE	44%
8	$AgSbF_6$	DCM/DMSO (50 mol%)	0%
9	$AgSbF_6$	DCM/TFA (50:1)	8%
10	$AgSbF_6$	DCM/DMSO (50:1)	0%
11	$AgSbF_6$	DCB/MeOH (50:1)	75%
**12**	**$AgSbF_6$**	**DCM/MeOH (50:1)**	**97%**
13	$AgSbF_6$	DCM/MeOH (120:1)	74%
14	$AgSbF_6$	DCM/MeOH (1:1)	40%
15	$AgSbF_6$	DCM/MeOH (1:3)	30%

*Reaction conditions: 1 eq of (P,N)AuCl (0.083 mmol), 2 eq of $AgSbF_6$, 1 eq of idodobenzene, 1 eq of 1,3,5–trimethoxybenzene, reaction time 5h, temperature: 25 °C.
#Yields determined using calibrated GC-MS analysis *vs.* *n*-dodecane as internal standard.

Supplementary Table 2. Optimization of catalytic conditions. Screening of catalyst loading, equivalent of arene, temperature, solvent and reaction time for the catalytic arylation of 1,3,5–trimethoxybenzene with iodobenzene.

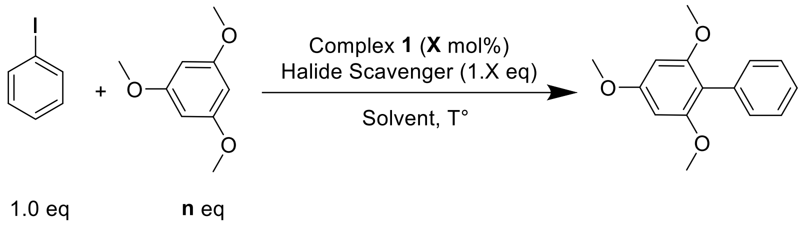

Entry	[Au] loading	ArH (equiv)	T(°C)	Solvent	Time (h)	Yield (%)[#]
1	20	1	25	DCM	21	30
1	20	1	75	DCB	14	82
2	10	1	75	DCB	21	69
3	5	1	75	DCB	21	46
4	5	5	75	DCB	21	58
5	5	10	75	DCB	14	88
6	5	10	75	Chlorobenzene	21	41
7	5	10	25	DCB/MeOH (50:1)	21	59
9	5	5	75	DCB/MeOH (50:1)	2	99
10	5	2	75	DCB/MeOH (50:1)	2	99
11	5	1	75	DCB/MeOH (50:1)	2	73
12	2.5	10	75	DCB/MeOH (50:1)	2	80

[#]Yields determined using calibrated GC-MS analysis *vs.* *n*-dodecane as internal standard.

Supplementary Table 3. Screening of halide scavengers.*

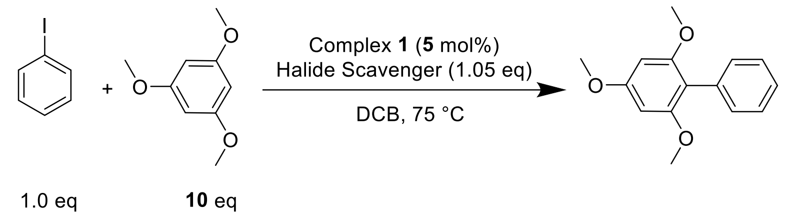

Entry	Halide Scavenger	Time (h)	Yield (%)[a]
1	$AgSbF_6$	14	88
2	$AgNTf_2$	21	41
3	AgOTf	21	62
4	NaBarf	21	0
5	AgOPiv	21	0

[*]Reaction conditions: [(P,N)AuCl] 5 mol %, $AgSbF_6$ (1.05 eq based on idodobenzene), idodobenzene, 1,3,5–trimethoxybenzene (10 eq based on iodobenzene), in Dichlorobenzene at 75 °C.
[#]Yields determined using calibrated GC-MS analysis vs. *n*-dodecane as internal standard.

Supplementary Table 4. Screening of bases.*

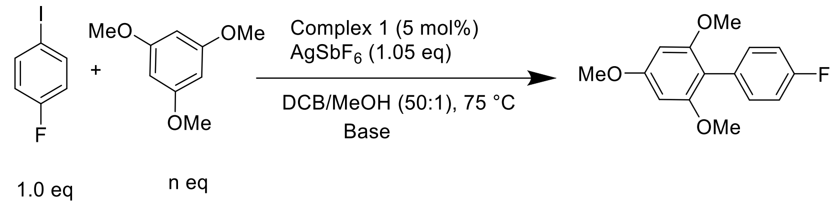

Entry	ArH (equiv)	Base	Yield (%)[#]
1	2	-	59
2	2	CS_2CO_3 (3eq)	58
3	2	CsOPiv (3eq)	0
4	2	NaOAc (3eq)	62
5	2	K_3PO_4 (3eq)	75
6	2	K_3PO_4 (1eq)	90
10	1	K_3PO_4 (1eq)	95
11[§]	1	K_3PO_4 (1eq)	90

[*]Reaction conditions: [(P,N)AuCl] 5 mol %, $AgSbF_6$ (1.05 eq based on iodoarene), 1-iodo-4-fluorobenzene, 1,3,5–trimethoxybenzene (n eq based on iodoarene), in Dichlorobenzene at 75 °C, reaction time 5h.

[#]Yields determined using calibrated GC-MS analysis vs. *n*-dodecane as internal standard.

[§]Reaction conditions: 1 mol % [[(P,N)AuCl] loading, reaction time 12 h.

Supplementary Table 5. Crystal data, data collection, and structure refinement for 2, 3, and 6.

	**2**	**3**	**6**
ID	sadp-1	AZ99	AZ100
formula	$C_{34}H_{45}AuINP,C_2F_6NO_4S_2$	$C_{35}H_{47}AuClNOP, F_6Sb$	$C_{34}H_{44}AuClN_2O_2P,F_6Sb,0.5(CH_2Cl_2)$
M_r	1102.70	996.89	1054.31
crystal system	triclinic	triclinic	monoclinic
space group	$P\bar{1}$	$P\bar{1}$	$C2/c$
a (Å)	10.5352(6)	9.6514(10)	9.8859(3)
b (Å)	13.7465(8)	13.1781(12)	30.1443(10)
c (Å)	15.7351(9)	15.6941(13)	26.6076(10)
α (°)	111.921(2)	110.458(4)	90
β (°)	108.705(2	104.687(4)	100.225(2)
γ (°)	94.489(2)	93.177(5)	90
V (Å^3)	1950.9(2)	1786.1(3)	7803.2(5)
Z	2	2	8
ρ_{calc} (g cm^{-3})	1.877	1.854	1.795
μ (mm^{-1})	4.780	5.039	4.688
F(000)	1080	976	4120
crystal size (mm^3)	0.14 x 0.09 x 0.03	0.08 x 0.08 x 0.04	0.12 x 0.12 x 0.04
T/K	173(2)	193(2)	193(2)
measd reflns	52880	25853	28399
Unique reflns (Rint)	9743 (0.0762)	7188 (0.0681)	7845 (0.0517)
reflns used for refinement	9743	7188	7845
refined parameters	616	503	768
GOF on F^2	1.020	1.040	1.042
R_1[a] [I>2σ(I)]	0.0490	0.0448	0.0421
wR_2[b] [all data]	0.1168	0.0871	0.0942

[a] $R_1 = \Sigma||F_o| - |F_c||/ \Sigma |F_o|$. [b] $wR_2 = [\Sigma[w(F_o^2 - F_c^2)^2] / \Sigma [w(F_o^2)^2]]^{1/2}$.

Supplementary Table 6. Crystal data, data collection, and structure refinement for 7, 9 and 12

	**7**	**9**	**12**
ID	AZ348	AZ370	AZ116
formula	$C_{34}H_{40}AuCl0.75F_5$ $I0.25NPF_6Sb$, 1.25(CH_2Cl_2)	$C_{35}H_{46}AuClN_2O_2P$, F_6Sb, 2(CH_2Cl_2)	$C_{40}H_{48}AuNP$, F_6Sb, CH_2Cl_2
M_r	1185.84	1195.74	1091.42
crystal system	triclinic	triclinic	monoclinic
space group	$P\bar{1}$	$P\bar{1}$	$P2_1/c$
a (Å)	10.0587(3)	10.2280(6)	16.0205(6)
b (Å)	11.4843(4)	11.6142(7)	12.5672(5)
c (Å)	17.2888(6)	18.2296(9)	20.2378(8)
α (°)	91.662(2)	96.603(2)	90
β (°)	92.440(2)	92.475(2)	101.436(2)
γ (°)	98.828(2)	101.478(3)	90
V (Å^3)	1970.43(11)	2103.3(2)	3993.6(3)
Z	2	2	4
ρ_{calc} (g cm^{-3})	1.999	1.888	1.815
μ (mm^{-1})	4.941	4.544	4.579
F(000)	1147.0	1172.0	2144.0
crystal size (mm^3)	0.20 x 0.18 x 0.10	0.16 x 0.12 x 0.02	0.16 x 0.14 x 0.04
T/K	193(2)	193(2)	193(2)
measd reflns	44532	47289	140944
Unique reflns (Rint)	11927 (0.0225)	12742 (0.0382)	14257 (0.0441)
reflns used for refinement	11927	12742	14257
refined parameters	512	499	570
GOF on F^2	1.076	1.165	1.058
R_1[a] [I>2σ(I)]	0.0278	0.0368	0.0278
wR_2[b] [all data]	0.0687	0.1413	0.0607

[a] $R_1 = \Sigma||F_o| - |F_c||/ \Sigma |F_o|$. [b] $wR_2 = [\Sigma[w(F_o^2 - F_c^2)^2] / \Sigma [w(F_o^2)^2]]^{1/2}$.

Supplementary Table 7. Main geometrical features associated with *minima*, TS located for the oxidative addition of PhI to complex 1-NTf$_2$, and 1-SbF$_6$. (distances in Å and bond angles in °).

(P,N)Au$^+$-X$^-$	PAu	NAu	AuI	IC$_{ipso}$	AuC$_{ipso}$	AuC$_{ortho}$	PAuN	C$_{ipso}$AuI
				X$^-$: SbF$_6^-$				
**1-SbF$_6$[a]**	2.238	2.760	/	/	/	/	78.5	/
**PhI**	/	/	/	2.184	/	/	/	/
**TS$_{exchange}$**	2.261	2.860	3.164 (Au-F : 2.819)	2.178	3.413	3.528	76.0	38.4
**I-adduct-SbF$_6$**	2.273	2.863	2.846	2.185	3.414	3.522	76.1	39.6
**TS1-SbF$_6$[b]**	2.307	2.670	2.750	2.438	2.489	3.243	79.8	55.2
**2-SbF$_6$[c]**	2.367	2.350	2.792	3.258	2.085	3.003	82.9	82.5
				X$^-$: NTf$_2^-$				
**1-NTf$_2$[d]**	2.158	3.043	/	/	/	/	73.3	/
**I-adduct-NTf$_2$[e]**	2.282	2.843	2.852	2.181	3.527	3.520	76.5	38.2
**TS$_{exchange}$**	2.286	3.158	2.870 (Au-N$_{NTf2}$: 3.068)	2.170	3.676	3.676	71.90	36.14
**TS1[f]**	2.317	2.658	2.747	2.442	2.471	3.199	78.6	55.5
**2-NTf$_2$[g]**	2.362	2.344	2.790	3.278	2.082	3.025	82.7	83.2

[a]d(Au-F): 2.239 Å; [b]d(Au-F): 3.213 Å; [c]d(Au-F): 3.137 Å. [d]d(Au-N[NTf$_2$]): 2.158 Å; [e]d(Au-N[NTf$_2$]): 3.078 Å; [f]d(Au-N[NTf$_2$]): 3.829 Å; [g]d(Au-N[NTf$_2$]): 4.360 Å.

Supplementary Table 8. Main geometrical features associated with *minima* and TS located along the reaction profile for the oxidative addition of PhI to cationic complex 1* leading to the other stereosiomer $2*_1$. (distances in Å and bond angles in °), into brackets [] are reported geometrical structures for the other isomer (I in *trans* to P, **2***) calculated in gas phase.

.	**PAu**	**NAu**	**AuI**	**IC_{ips}**	**AuC_{ipso}**	**AuC_{ortho}**	**PAuN**	**$C_{ipso}AuI$**
**1***	2.280	2.700	/	/	/	/	80.5	/
**π-adduct***	2.298	2.779	2.779	2.147	3.002	2.400	76.6	29.6
**[I-adduct*]**	[2.289]	[2.746]	[2.782]	[2.204]	[3.347]	[3.606]	[77.5]	[40.9]
**TS_1*_1**	2.326	2.639	2.875	2.432	2.219	2.963	78.6	55.2
**[TS_1*]**	[2.309]	[2.639]	[2.738]	[2.443]	[2.488]	[3.234]	[79.0]	[55.5]
**$2*_1$**	2.401	2.305	2.675	3.192	2.098	3.037	83.3	83.0
[2*]	[2.369]	[2.368]	[2.723]	[3.246]	[2.092]	[3.019]	[82.2]	[83.5]

Supplementary Table 9. Main geometrical features associated with *minima* and TS located along the reaction profile for the oxidative addition of PhI to 1_H-SbF_6. (distances in Å and bond angles in °).

	PAu	AuI	IC_{ipso}	AuC_{ipso}	AuC_{ortho}	PAuH	$C_{ipso}AuI$
**1_H**[a]	2.244	/	/	/	/	/	/
**I-adduct$_H$**	2.282	2.809	2.188	3.328	3.511	68.9	40.7
**$TS_{exchange}$**	2.255	3.165 (Au-F : 3.051)[a]	2.178	3.471	3.396	70.80	37.96
**TS_{1H}**	2.343	2.728	2.647	2.263	3.060	69.5	63.3
**2_H**	2.385	2.736	3.384	2.070	2.962	69.4	88.4

[a] Au-F (**in 1_H**): 2.223 Å

Supplementary Table 10. Main geometrical features associated with *minima* and TS located along the reaction profile for the oxidative addition of PhBr to complex 1-SbF_6. (distances in Å and bond angles in °)

R	PAu	NAu	AuBr	BrC_{ipso}	AuC_{ipso}	AuC_{ortho}	PAuN	$C_{ipso}AuBr$
**1-SbF_6**	2.280	2.700					80.5	
**$TS_{exchange}$**	2.263	2.749	3.107 Au-F:2.534	1.981	3.511	3.521	78.7	34.1
**Br-adduct**	2.277	2.750	2.681	2.017	3.310	3.506	77.9	37.5
**TS-SbF_6**	2.298	2.633	2.594	2.289	2.465	3.225	79.2	53.7
**(P,N)AuBr(Ph)/SbF_6**	2.349	2.357	2.548	3.131	2.088	2.983	82.7	84.4

C_{ipso}-Br (in PhBr) : 1.989 Å.

Supplementary Methods

General

Unless otherwise stated, all reactions and manipulations were carried out under an atmosphere of dry argon using standard Schlenk techniques or in a glovebox under an inert atmosphere. Dry, oxygen-free solvents were employed. Solution ^{1}H, ^{13}C, ^{31}P and ^{19}F NMR spectra were recorded on Bruker Avance 300, 400 or 500 spectrometers at 298K unless otherwise stated. Chemical shifts (*δ*) are expressed with a positive sign, in parts per million. ^{1}H and ^{13}C chemical shifts reported are referenced internally to residual protio- (^{1}H) or deutero – (^{13}C) solvent, while ^{31}P and ^{19}F chemical shifts are relative to 85% H_3PO_4 and $CFCl_3$ respectively. The following abbreviations and their combinations are used: br, broad; s, singlet; d, doublet; t, triplet; q, quartet; m, multiplet. The ^{1}H and ^{13}C resonance signals were attributed by means of 2D HSQC and HMBC. Mass spectra were recorded on a Waters UPLC Xevo G2 Q TOF apparatus. GC-MS analyses were performed on a MS Perkin Elmer Clarus MS560, GC PerkinElmer Clarus 500 and Agilent HP6890. Melting points were determined with a calibrated Stuart SMP40 (PT1000) apparatus. All starting materials were purchased from Aldrich and used as received unless otherwise stated.

Oxidative addition of iodobenzene with complex 1 in the presence of $AgNTf_2$

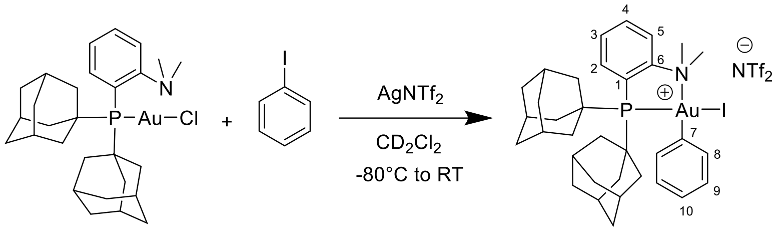

1 **2**

In a glovebox, a screw-cap NMR tube was charged with silver bis(trifluoromethanesulfonyl)imide (12.0 mg, 0.031 mmol) in dichloromethane-d^2 (0.3 mL). Complex **1** (20 mg, 0.031 mmol) was transferred into a small glass vial and dissolved in dichloromethane-d^2 (0.3 mL). The prepared solution was loaded into a plastic syringe equipped with stainless steel needle. Outside the glovebox, the NMR tube was cooled down to –80°C (Ethanol/N_2 cold bath). At this temperature, the solution of complex **1** was added to the $AgNTf_2$ solution. The tube was gently shaken and allowed to warm to RT. After formation of the cationic gold(I) complex **1/AgNTf$_2$**, iodobenzene (3.5 µL, 0.031 mmol) was added to the NMR tube at room temperature. The reaction was monitored by ^{31}P NMR spectroscopy. Complete conversion of **1/AgNTf$_2$** is observed after 48 h to give complex **2** (92 % NMR spectroscopic yield), along with unidentified species. Complex **2** was characterized by ^{1}H and ^{13}C NMR spectroscopy at room temperature and by high-resolution mass spectrometry (HRMS). The ESI mass spectrum showed the presence of two peaks corresponding to the mass of $[(P,N)AuPh(I)]^+$ **(2)** and $[(P,N)AuPh(Cl)]^+$ **(2')**, indicating that some iodide to chloride exchange occurs at gold(III). The chloride source probably comes from dichloromethane and/or the silver salt AgCl. Halide exchange reactions to form more stable aryl-gold(III) chlorides derivatives are consistent with the reported bonding strength of halides to gold(III).[1] Crystals suitable for X–ray diffraction were obtained directly from a concentrated solution of **2** in dichloromethane-d^2.

^{1}H NMR (300 MHz, CD_2Cl_2): δ 8.01-7.94 (m, 2H, H_{Ar}), 7.92-7.86 (m, 1H, H_{Ar}), 7.79-7.67 (m, 1H, H_{Ar}), 7.48-7.46 (m, 2H, H_{Ar}), 7.38-7.23 (m, 3H, H_{Ar}), 3.50 (s, 6H, $N(CH_3)_2$), 2.34-2.09 (m, 18H, H_{Ad}), 1.75 (s, 12H, H_{Ad}). **^{31}P{^{1}H} NMR** (121 MHz, CD_2Cl_2): δ 74.2 (s). **^{13}C{^{1}H} NMR** (126 MHz, CD_2Cl_2): δ 158.8 (d, $J_{P\text{-}C}$ = 6.9 Hz, C_6), 137.0 (d, $J_{P\text{-}C}$ = 2.3 Hz, C_5), 136.8 (d, $J_{P\text{-}C}$ = 1.6 Hz, C_4), 135.2 (s, C_9), 130.5 (s, C_3), 130.4 (s, C_8), 128.3 (s, C_{10}), 127.7 (s, C_7), 125.6 (d, $J_{P\text{-}C}$ = 7.3 Hz, C_2), 119.1 (d, $J_{P\text{-}C}$ = 44.0 Hz, C_1), 53.9 (s, $N(\underline{C}H_3)_2$), 48.2 (d, $J_{P\text{-}C}$ = 13.6 Hz, C_{qtAd}), 40.7 (d, $J_{P\text{-}C}$ = 1.3 Hz, CH_{2Ad}), 35.8 (d, $J_{P\text{-}C}$ = 2.0 Hz, CH_{2Ad}), 28.9 (d, $J_{P\text{-}C}$ = 9.8 Hz, CH_{Ad}). **HRMS (ESI+)**: calculated for $[M^+]$ = $C_{34}H_{45}NPIAu^+$: 822.2000. Found: 822.2006. For $[M'^+]$ = $C_{34}H_{45}NPClAu^+$: 730.2644. Found: 730.2657.

Preparation of gold(III) aryl complex 2'

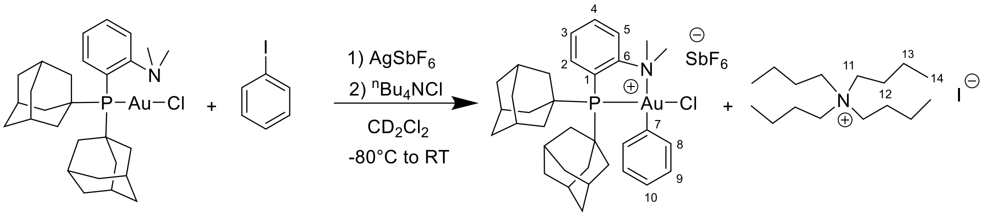

1 **2'**

In a glovebox, a screw-cap NMR tube was charged with silver hexafluoroantimonate (10.7 mg, 0.031 mmol) in dichloromethane-d[2] (0.3 mL). Complex **1** (20 mg, 0.031 mmol) was transferred into a small glass vial and dissolved in dichloromethane-d[2] (0.3 mL). Iodobenzene (3.5 µL, 0.031 mmol) was added to the solution of complex **1**. The prepared solution was loaded into a plastic syringe equipped with stainless steel needle. The syringe was closed by blocking the needle with a septum. Outside the glovebox, the NMR tube was cooled down to –80°C (Ethanol/N_2 cold bath). At this temperature, the solution of complex **1** and iodobenzene was added to the $AgSbF_6$ solution. The tube was gently shaken and allowed to warm to room temperature. After complete conversion (as indicated by ^{31}P NMR monitoring), the tube was reintroduced in the glovebox, and tetrabutylammonium chloride (8.6 mg, 0.031 mmol) was added at room temperature to completely convert complex **2** into the corresponding aryl gold(III)–chloride complex **2'**, which was isolated in 80 % yield.

^{1}H NMR (500 MHz, CD_2Cl_2): δ 8.34 (dd, $^3J_{H\text{-}H}$ = 4.0 Hz, $^3J_{P\text{-}H}$ = 8.23 Hz, 1H, H_2), 8.02-7.99 (m, 1H, H_5), 7.90-7.87 (pseudo t, 1H, H_4), 7.75-7.72 (m, 1H, H_3), 7.50-7.48 (m, 2H, H_9), 7.31 (t, $^3J_{H\text{-}H}$ = 7.47 Hz, 2H, H_8), 7.25-7.22 (m, 1H, H_{10}), 3.56 (s, 6H, N(C*H*$_3$)$_2$), 3.24-3.20 (m, 8H, H_{11}), 2.31-2.02 (m, 18H, H_{Ad}), 1.79-1.60 (m, 20H, H_{Ad} & H_{12}), 1.42 (sextuplet, $^3J_{H\text{-}H}$ = 7.38 Hz, 8H, H_{13}), 1.00 (t, $^3J_{H\text{-}H}$ = 7.32 Hz, 12H, H_{14}). **^{31}P{^{1}H} NMR** (121 MHz, CD_2Cl_2): δ 74.4 (s). **^{13}C{^{1}H}** (126 MHz, CD_2Cl_2): δ 159.2 (d, $J_{P\text{-}C}$ = 6.8 Hz, C_6), 137.2 (d, $J_{P\text{-}C}$ = 2.2 Hz, C_5), 136.7 (d, $J_{P\text{-}C}$ = 1.8 Hz, C_4), 135.4 (s, C_9), 130.5 (s, C_3), 130.4 (s, C_8), 128.3 (s, C_{10}), 128.0 (s, C_7), 126.5 (d, $J_{P\text{-}C}$ = 7.3 Hz, C_2), 118.9 (d, $J_{P\text{-}C}$ = 44.5 Hz, C_1), 58.9 (s, C_{11}), 54.1 (s, N($\underline{C}H_3$)$_2$), 48.3 (d, $J_{P\text{-}C}$ = 13.8 Hz, C_{qtAd}), 40.8 (d, $J_{P\text{-}C}$ = 1.1 Hz, CH_{2Ad}), 35.9 (d, $J_{P\text{-}C}$ = 1.7 Hz, CH_{2Ad}), 29.1 (d, $J_{P\text{-}C}$ = 9.8 Hz, CH_{Ad}), 24.4 (s, C_{12}), 20.2 (s, C_{13}), 13.9 (s, C_{14}). **HRMS (ESI+)**: calculated for [M^+] = $C_{34}H_{45}NPClAu^+$: 730.2644. Found: 730.2633.

Oxidative addition of biphenylene to complex 1 in the presence of $AgSbF_6$

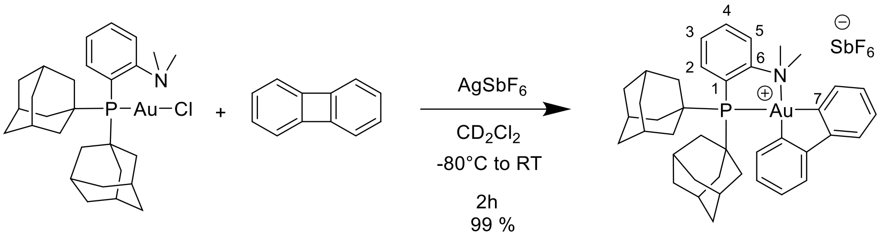

1 **12**

In a glovebox, a screw-cap NMR tube was charged with silver hexafluoroantimonate (8.0 mg, 0.023mmol) in dichloromethane-d^2 (0.3 mL). Complex **1** (15 mg, 0.023 mmol) was transferred into a small glass vial and dissolved in dichloromethane-d^2 (0.3 mL). Biphenylene (35.0 mg, 0.230 mmol) was added to the solution of complex **1**. The prepared solution was loaded into a plastic syringe equipped with stainless steel needle. The syringe was closed by blocking the needle with a septum. Outside the glovebox, the NMR tube was cooled down to -80°C (Ethanol/N_2 cold bath). At this temperature, the solution of complex **1** and biphenylene was added to the $AgSbF_6$ solution. The tube was gently shaken and allowed to warm to RT. Crystals suitable for X-ray diffraction were obtained from a concentrated solution of **12**.

^{1}H NMR (300 MHz, CD_2Cl_2): δ 8.21 (d, J_{HH} = 7.65 Hz, 1H, H_{Ar}), 8.01 (t, J_{HH} = 7.02 Hz, 1H, H_{Ar}), 7.91-7.85 (m, 2H, H_{Ar}), 7.67-7.44 (m, 4H, H_{Ar}), 7.37-7.29 (m, 3H, H_{Ar}), 7.03-6.98 (m, 1H, H_{Ar}), 3.72 (s, 6H, $N(CH_3)_2$), 2.35-2.09 (m, 18H, H_{Ad}), 1.77-1.65 (m, 12H, H_{Ad}). **^{31}P{^{1}H} NMR** (121 MHz, CD_2Cl_2): δ 76.7 (s). **^{13}C{^{1}H}** (126 MHz, CD_2Cl_2): δ 172.6 (d, J_{CP} = 116.3 Hz, C7), 163.5 (d, J_{CP} = 11.6 Hz, C6), 154.4 (d, J_{CP} = 3.6 Hz, $C_{biphenyl}$), 153.5 (d, J_{CP} = 1.6 Hz, $C_{biphenyl}$), 150.3 (d, J_{CP} = 5.0 Hz, $C_{biphenyl}$), 142.3 (d, J_{CP} = 6.8 Hz, $CH_{biphenyl}$), 137.5 (s, C4 or C5), 136.3 (s, C4 or C5), 131.3 (d, J_{CP} = 4.7 Hz, C3), 126.6 (d, J_{CP} = 2.5 Hz, $CH_{biphenyl}$), 125.9 (s, $CH_{biphenyl}$), 125.1 (d, J_{CP} = 7.2 Hz, C2), 123.9 (s, $CH_{biphenyl}$), 123.1 (d, J_{CP} = 5.5 Hz, $CH_{biphenyl}$), 120.0 (d, J_{CP} = 38.1 Hz, C1), 55.70 (s, $N(\underline{C}H_3)_2$), 46.6 (d, J_{CP} = 6.9 Hz, C_{qtAd}), 42.60 (s, CH_{2Ad}), 36.10 (s, CH_{2Ad}), 29.6 (d, J_{CP} = 9.3 Hz, CH_{Ad}). **HRMS (ESI+):** calculated for [M+] = $C_{40}H_{48}NPAu^+$: 770.3190. Found: 770.3198.

General procedure for the reaction of complex 1 with aryl Iodides.

In a glovebox, a screw-cap NMR tube was charged with silver hexafluoroantimonate (8.0 mg, 0.023 mmol) in dichloromethane-d^2 (0.3 mL). Complex **1** (15 mg, 0.023 mmol) was transferred into a small glass vial and dissolved in dichloromethane-d^2 (0.3 mL). The aryl iodide (0.115 mmol) was added to the solution of **1**. The prepared solution was loaded into a plastic syringe equipped with stainless steel needle. The syringe was closed by blocking the needle with a septum. Outside the glovebox, the NMR tube was cooled down to -80°C (Ethanol/N_2 cold bath). At this temperature, the solution of complex **1** and aryl iodide was added. The tube was gently shaken and allowed to warm to RT. The reaction was left to proceed until completion as monitored by $^{31}P\{^1H\}$ NMR. The formation of the gold(III) complex **3-10** was confirmed by ^1H and ^{31}P NMR spectroscopy and high-resolution mass spectrometry (Electrospray ionization, positive mode). Halide exchange reaction from aryl gold(III) complexes was systematically observed with all substrates. In the following examples, only the mass peak corresponding to the aryl gold(III) chloride derivatives is indicated. Complexes **3**, **6**, **7** and **9** were also characterized in the solid state by X-ray diffraction analysis.

Reaction of Complex 1 with 4-Methoxyiodobenzene (3)

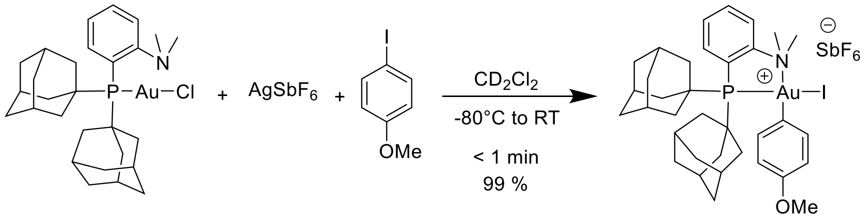

^{1}H NMR (300 MHz, CD_2Cl_2): δ 8.00-7.87 (m, 3H, H_{Ar}), 7.80-7.71 (m, 1H, H_{Ar}), 7.36-7.33 (d, J_{HH} = 8.89 Hz, 2H, H_{Ar}), 6.94-6.91 (d, J_{HH} = 8.95 Hz, 2H, H_{Ar}), 3.83 (s, 3H, OMe), 3.49 (s, 6H, $N(CH_3)_2$), 2.30-2.01 (m, 18H, H_{Ad}), 1.76 (bs, 12H, H_{Ad}). **^{31}P{^{1}H} NMR** (121 MHz, CD_2Cl_2): δ 75.1 (s). **HRMS (ESI+)**: calculated for $[M^+]$ = $C_{35}H_{47}NOPClAu^+$: 760.2749. Found: 760.2756.

Reaction of Complex 1 with 4-Methyliodobenzene (4)

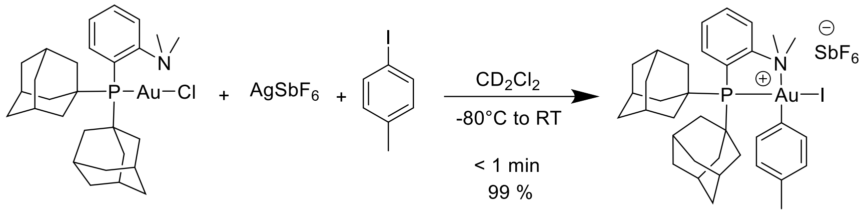

^{1}H NMR (300 MHz, CD_2Cl_2): δ 8.02-7.88 (m, 3H, H_{Ar}), 7.79-7.71 (m, 1H, H_{Ar}), 7.35-7.31 (m, 2H, H_{Ar}), 7.16-7.13 (m, 2H, H_{Ar}), 3.49 (s, 6H, $N(CH_3)_2$), 2.38 (s, 3H, CH_3), 2.30-2.05 (m, 30H, H_{Ad} and $CH_{3excess}$), 1.76 (bs, 12H, H_{Ad}). **^{31}P{^{1}H} NMR** (121 MHz, CD_2Cl_2) : δ 74.2 (s). **HRMS (ESI+)**: calculated for [M^+] = $C_{35}H_{47}NPClAu^+$: 744.2800. Found: 744.2799.

Reaction of Complex 1 with 4-Fluoroiodobenzene (5)

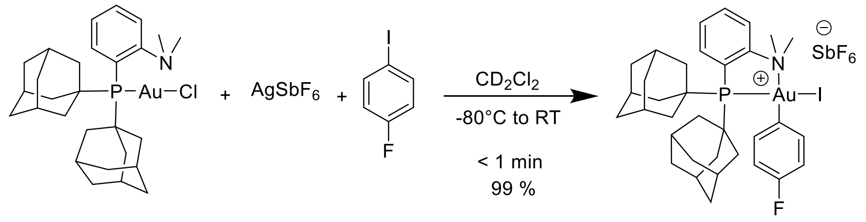

^{1}H NMR (300 MHz, CD_2Cl_2): δ 8.02-7.88 (m, 3H, H_{Ar}), 7.78-7.72 (m, 1H, H_{Ar}), 7.51-7.46 (m, 2H, H_{Ar}), 7.14 (t, J_{HH} = 8.67 Hz, 2H, H_{Ar}), 3.52 (s, 6H, $N(CH_3)_2$), 2.42-2.04 (m, 18H, H_{Ad}), 1.76 (s, 12H, H_{Ad}). **^{31}P{^{1}H} NMR** (121 MHz, CD_2Cl_2): δ 76.03 (s). **HRMS (ESI+)**: calculated for [M^+] = $C_{34}H_{44}NPClFAu^+$: 748.2549. Found: 748.2562.

Reaction of Complex 1 with 4-Nitroiodobenzene (6)

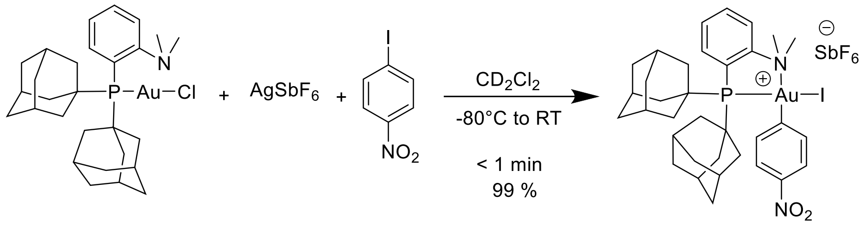

^{1}H NMR (300 MHz, CD_2Cl_2): δ 8.21-8.16 (m, H_{Ar}), 8.03-7.98 (m, H_{Ar}), 7.97-7.88 (m, H_{Ar}), 7.81-7.74 (m, H_{Ar}), 3.56 (s, 6H, $N(CH_3)_2$), 2.38-2.02 (m, 18H, H_{Ad}), 1.77 (s, 12H, H_{Ad}). **^{31}P{^{1}H} NMR** (121 MHz, CD_2Cl_2): δ 79.7 (s). **HRMS (ESI+)**: calculated for [M^+] = $C_{34}H_{44}N_2O_2PClAu^+$: 775.2494. Found: 775.2482.

Reaction of Complex 1 with Iodopentafluorobenzene (7)

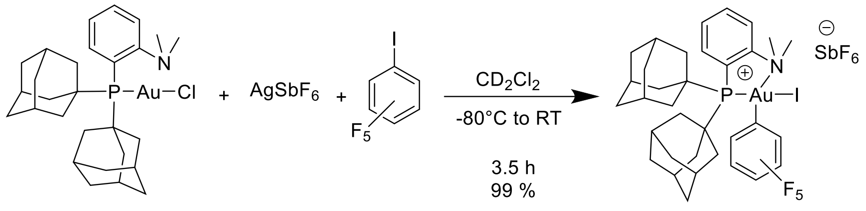

1 **7**

^{1}H NMR (300 MHz, CD_2Cl_2): δ 8.09-7.95 (m, 3H, H_{Ar}), 7.85-7.81 (m, 1H, H_{Ar}), 7.89-7.85 (m, 6H, H_{Ar}), 3.68 (s, 6H, $N(CH_3)_2$), 2.39-1.97 (m, 18H, H_{Ad}), 1.77 (s, 12H, H_{Ad}). **^{31}P{^{1}H} NMR** (121 MHz, CD_2Cl_2): δ 102.3 (s). **HRMS (ESI+)**: calculated for [M^+] = $C_{34}H_{40}NF_5PClAu^+$: 820.2174. Found: 820.2182.

Reaction of Complex 1 with 1-Iodonaphthalene (8)

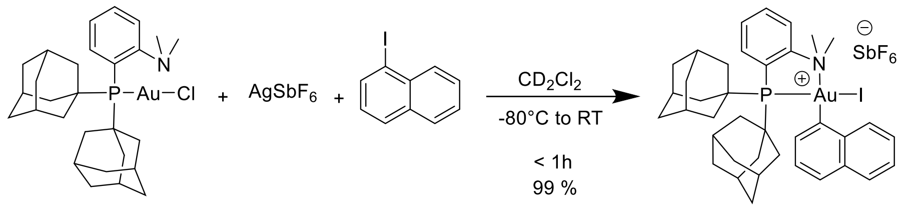

1 **8**

^{1}H NMR (300 MHz, CD_2Cl_2): δ 8.11-8.04 (m, 8H, H_{Ar}), 7.99-7.92 (m, 3H, H_{Ar}), 7.89-7.85 (m, 6H, H_{Ar}), 7.82-7.74 (m, 7H, H_{Ar}), 7.62-7.48 (m, 10H, H_{Ar}), 7.44-7.39 (t, $J_{H\text{-}H}$ = 7.79 Hz, 1H, H_{Ar}), 7.23-7.18 (m, 4H, H_{Ar}), 3.59 (d, J_{HH} = 6.17 Hz, 6H, $N(CH_3)_2$), 2.45-1.27 (m, 30H, H_{Ad}). **^{31}P{^{1}H} NMR** (121 MHz, CD_2Cl_2) : δ 79.7 (s). **HRMS (ESI+)**: calculated for [M^+] = $C_{38}H_{47}NPClAu^+$: 780.2820. Found: 780.2810.

Reaction of Complex 1 with 2-Iodo-5-nitrotoluene (9)

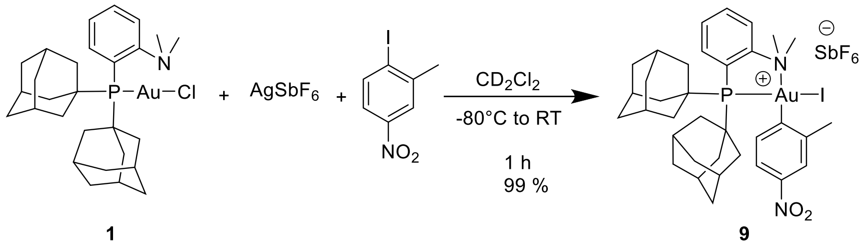

1 **9**

^{1}H NMR (300 MHz, CD_2Cl_2): δ 8.07-8.00 (m, 13H, H_{Ar}), 7.80-7.76 (m, 2H, H_{Ar}), 7.72-7.71 (m, 2H, H_{Ar}), 7.70-7.68 (m, 2H, H_{Ar}), 3.55 (d, J_{HH} = 15.0 Hz, 6H, $N(CH_3)_2$), 2.39 (s, 3H, CH_3), 2.39-1.59 (m, 30H, H_{Ad}). **^{31}P{^{1}H} NMR** (121 MHz, CD_2Cl_2): δ 80.8 (s). **HRMS (ESI+)**: calculated for [M^+] = $C_{35}H_{46}N_2O_2PClAu^+$: 789.2651. Found: 789.2650

Reaction of Complex 1 with 2-Iodopyridine (10)

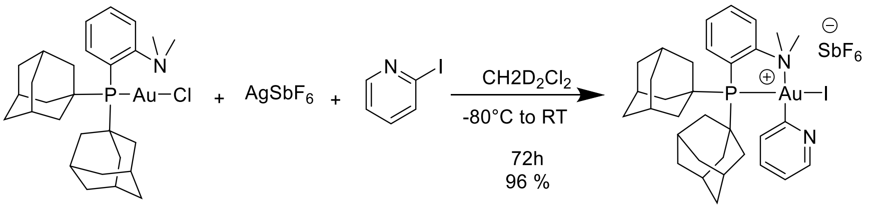

1 10

^{1}H NMR (300 MHz, CD_2Cl_2): δ 8.48-8.46 (m, 1H, H_{Ar}), 8.37-8.32 (m, 4H, H_{Ar}), 7.98-7.87 (m, 3H, H_{Ar}), 7.76-7.74 (m, 5H, H_{Ar}), 7.63-7.62 (m, 1H, H_{Ar}), 7.48-7.46 (m, 1H, H_{Ar}), 7.40 – 7.28 (m, 8H, H_{Ar}), 7.22-7.19 (m, 1H, H_{Ar}), 3.45 (s, 6H, $N(CH_3)_2$), 2.38-1.97 (m, 18H, H_{Ad}), 1.75 (s, 12H, H_{Ad}). **$^{31}P\{^1H\}$ NMR** (121 MHz, CD_2Cl_2): δ 70.0 (s). **HRMS (ESI+)**: calculated for $[M^+]$ = $C_{33}H_{44}N_2PClAu^+$: 731.2596. Found: 731.2590.

Reaction of Complex 1 with 1-Bromonaphthalene (11)

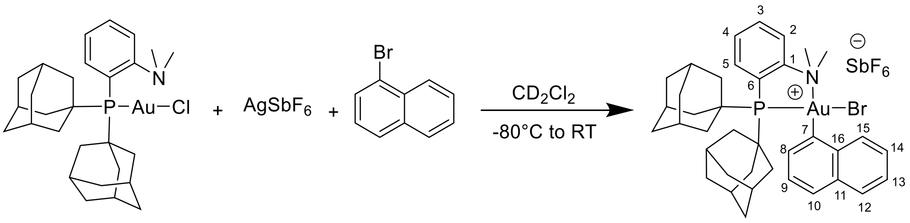

1 11

In a glovebox, a screw-cap NMR tube was charged with silver hexafluoroantimonate (10.7 mg, 0.031 mmol) in dichloromethane-d^2 (0.3 mL). Complex **1** (20 mg, 0.031 mmol) was transferred into a small glass vial and dissolved in dichloromethane-d^2 (0.3 mL). 1-Bromonaphthalene (4.3 µL, 0.031 mmol) was added to the solution of complex **1**. The prepared solution was loaded into a plastic syringe equipped with stainless steel needle. The syringe was closed by blocking the needle with a septum. Outside the glovebox, the NMR tube was cooled down to -80°C (Ethanol/N_2 cold bath). At this temperature, the solution of complex **1** and bromonaphthalene was added to the $AgSbF_6$ solution. The tube was gently shaken and allowed to warm to room temperature. Complete conversion to complex **11** (93 % spectroscopic yield) and a minor unidentified species was observed after 4 h. Complex **11** was spectroscopically characterized at room temperature from the reaction mixture.

^{1}H NMR (500 MHz, CD_2Cl_2): 8.07-8.02 (m, 3H, H_3, H_4 & H_{15}), 7.93-7.88 (m, 2H, H_2 & H_{12}), 7.83-7.80 (m, 2H, H_5 & H_8), 7.78-7.70 (m, 2H, H_{10} & H_{14}), 7.60-7.53 (m, 1H, H_{13}), 7.44 (t, J_{H-H} = 7.75 Hz, 1H, H_9), 3.60 (d, J_{CP} = 8.50 Hz, 6H, $N(CH_3)_2$), 2.37-1.41 (m, 30H, H_{Ad}). **$^{31}P\{^1H\}$**

NMR (121 MHz, CD_2Cl_2): δ 76.8 (s). **^{13}C{^{1}H}** (126 MHz, CD_2Cl_2): δ 158.2 (d, J_{P-C} = 7.1 Hz, C_1), 137.0 (d, J_{P-C} = 2.3 Hz, C_3), 136.6 (s, C_{16}), 136.4 (d, J_{P-C} = 1.7 Hz, C_2), 136.1 (s, C_{11}), 132.4 (s, C_8), 131.2 (s, C_{15}), 130.8 (d, J_{P-C} = 7.3 Hz, C_5), 129.1 (s, C_{10} & C_{12}), 128.9 (s, C_7), 127.3 (s, C_{14}), 127.0 (s, C_{13}), 126.3 (s, C_9), 125.6 (d, J_{P-C} = 7.2 Hz, C_4), 119.7 (d, J_{P-C} = 44.1 Hz, C_6), 54.0 (s, $N(\underline{C}H_3)_2$) , 53.5 (s, $N(\underline{C}H_3)_2$), 47.9 (d, J_{P-C} = 13.8 Hz, C_{qtAd}), 41.1 (d, J_{P-C} = 1.4 Hz, CH_{2Ad}), 39.5 (d, J_{P-C} = 1.4 Hz, CH_{2Ad}), 35.9 (d, J_{P-C} = 1.7 Hz, CH_{2Ad}), 35.3 (d, J_{P-C} = 1.8 Hz, CH_{2Ad}), 29.1 (d, J_{P-C} = 9.8 Hz, CH_{Ad}), 28.7 (d, J_{P-C} = 9.8 Hz, CH_{Ad}). **HRMS (ESI +)**: calculated for [M^+] = $C_{38}H_{47}NPBrAu^+$: 824.2295. Found: 824.2297. For [M'^+] = $C_{38}H_{47}NPClAu^+$: 780.2800. Found: 780.2802.

General procedure for the reaction of complex 1 with aryl bromides

In a glovebox, a flame dried Schlenk equipped with a magnetic stirrer bar was charged with silver hexafluoroantimonate (32.0 mg, 0.092 mmol) in dichloromethane (3 mL). Complex **1** (60 mg, 0.092 mmol) was transferred into a small glass vial and dissolved in dichloromethane (2 mL). Aryl bromide (0.46 mmol, 5eq.) was added to the solution of complex **1**. The prepared solution was loaded into a plastic syringe equipped with stainless steel needle. The syringe was closed by blocking the needle with a septum. Outside the glovebox, the NMR tube was cooled down to – 80°C (Ethanol/N_2 cold bath). At this temperature, the solution of complex **1** and aryl bromide was added to the $AgSbF_6$ solution. The reaction mixture was then stirred at room temperature overnight (12h). The reaction mixture was then filtered to give a clear solution. Volatiles were removed *in vacuo*. The residue was washed with pentane (3 x 3 mL) and dried under vacuum to give a solid powder. The formation of the gold(III) complex was confirmed by ^{1}H and ^{31}P NMR analysis and high-resolution mass spectrometry (Electrospray ionization, positive mode). Complex **13** was also characterized in the solid state by X-ray diffraction analysis.

Reaction of Complex 1 with 2-bromoanisole (13)

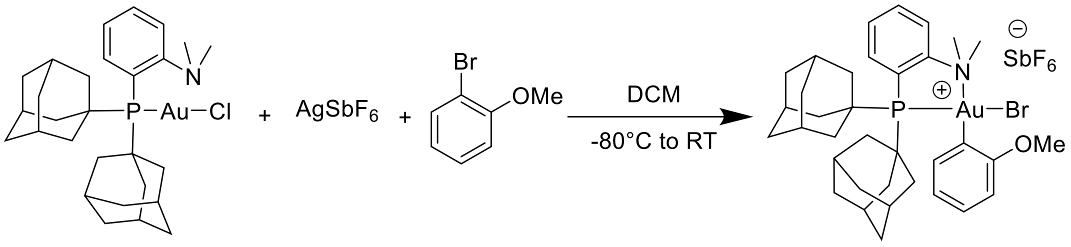

Yield 75 %. Mp: 211°C (decomposition). **^{1}H NMR** (300 MHz, CD_2Cl_2): δ 8.00-7.90 (m, 3H, H_{Ar}), 7.74 (t, J_{HH} = 7.81 Hz, 1H, H_{Ar}), 7.42 (d, J_{HH} = 7.98 Hz, 1H, H_{Ar}), 7.33-7.28 (m, 1H, H_{Ar}), 6.99 (t, J_{HH} = 7.81 Hz, 1H, $H_{Ar,}$), 6.85 (d, J_{HH} = 7.98 Hz, 1H, $H_{Ar,}$), 3.92 (s, 3H, O(C*H*$_3$)), 3.51 (d, J_{HH} = 10.91 Hz, N(C*H*$_3$)$_2$), 2.37-1.63 (m, 30H, H_{Ad}). **^{31}P{^{1}H} NMR** (121 MHz, CD_2Cl_2): δ 78.0 (s). **HRMS (ESI+)**: calculated for [M^+] = $C_{35}H_{47}NOPClAu^+$: 760.2749. Found: 760.2748. For [M'$^+$] = $C_{35}H_{47}NOPBrAu^+$: 804.2244. Found: 804.2238.

Reaction of Complex 1 with 4'-bromoacetophenone (14)

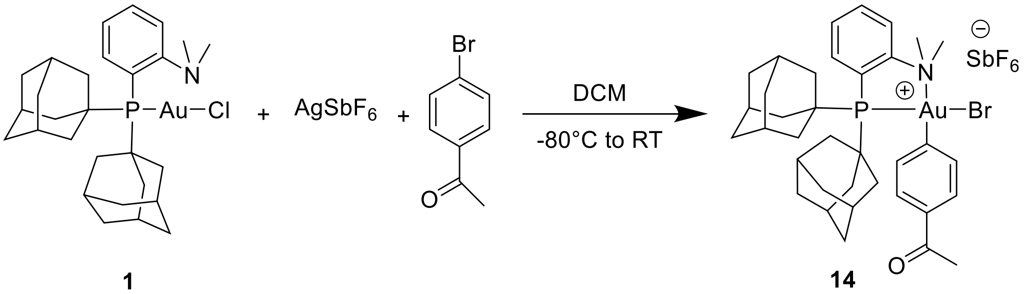

Yield 60 %. Mp: 201°C (decomposition). **^{1}H NMR** (300 MHz, CD_2Cl_2): δ 7.96-7.91 (m, 2H, H_{Ar}), 7.83 (d, J_{HH} = 8.70 Hz, 2H, H_{Ar}), 7.78-7.67 (m, 2H, H_{Ar}), 7.59 (d, J_{HH} = 8.70 Hz, 2H, $H_{Ar,}$), 3.47 (s, N(C*H*$_3$)$_2$), 2.56 (s, OC(C*H*$_3$)), 2.28-1.99 (m, 18H, H_{Ad}), 1.71 (br.s, 12H, H_{Ad}). **^{31}P{^{1}H} NMR** (121 MHz, CD_2Cl_2): δ 77.0 (s). **HRMS (ESI+)**: calculated for [M^+] = $C_{36}H_{47}NOPClAu^+$: 772.2749. Found: 772.2755. For [M'$^+$] = $C_{36}H_{47}NOPBrAu^+$: 816.2244. Found: 816.2233

The following complexes were detected by HRMS and $^{31}P\{^1H\}$ NMR spectroscopy:

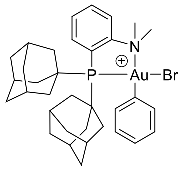

$^{31}P\{^1H\}$ NMR (121 MHz, CH_2Cl_2): δ 74.2 (s). **HRMS (ESI+)**: calculated for $[M^+] = C_{34}H_{45}NPBrAu^+$: 774.2139. Found: 774.2134.

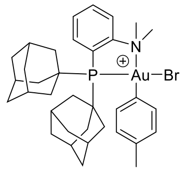

$^{31}P\{^1H\}$ NMR (121 MHz, CD_2Cl_2): δ 78.3 (s). **HRMS (ESI+)**: calculated for $[M^+] = C_{35}H_{47}NPBrAu^+$: 788.2295. Found: 788.2290.

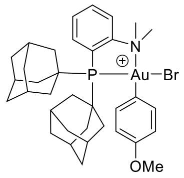

$^{31}P\{^1H\}$ NMR (121 MHz, CD_2Cl_2): δ 74.6 (s). **HRMS (ESI+)**: calculated for $[M^+] = C_{35}H_{47}NOPBrAu^+$: 804.2249. Found: 804.2255.

Direct arylation of 1,3,5–trimethoxybenzene with iodobenzene (stoichiometric conditions)

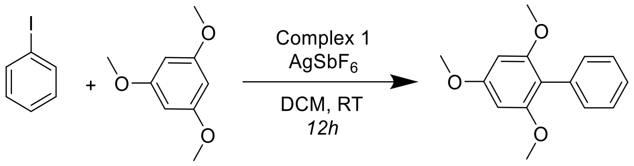

In a glovebox, a flame dried Schlenk equipped with a magnetic stirrer bar was charged with silver hexafluoroantimonate (57 mg, 0.166 mmol) in dichloromethane (1.0 mL). Complex **1** (54 mg, 0.083 mmol) was transferred into a small glass vial and dissolved in dichloromethane (1.0 mL). Iodobenzene (9.3 µL, 0.083 mmol) and 1,3,5-trimethoxybenzene (14.0 mg, 0.083 mmol) were added to the gold complex solution. This solution was loaded into a plastic syringe equipped with stainless steel needle. The syringe was closed by blocking the needle with a septum. Outside the glovebox, the Schlenk was cooled down to –80°C (Ethanol/N_2 cold bath). At this temperature, the solution of complex **1**, iodobenzene and 1,3,5-trimethoxybenzene was added. The reaction mixture was then stirred at room temperature until complete conversion (12h). After filtration of the silver salts, the sample was purified by column chromatography (pentane/ethyl acetate 100:0 to 95:5). The fractions containing the biaryl product were then concentrated *in vaccuo* to yield the pure product as a white crystalline solid (18 mg, 0.074 mmol, 90% yield). Analytical data are consistent with those previously reported.[2]

^{1}H NMR (300 MHz, $CDCl_3$): δ 7.41-7.29 (m, 5H, H_{Ar}), 6.23 (s, 2H, H_{Ar}), 3.87 (s, 3H, OCH_3), 3.72 (s, 6H, OCH_3).

Direct arylation of 1,3,5–trimethoxybenzene with iodobenzene under catalytic conditions

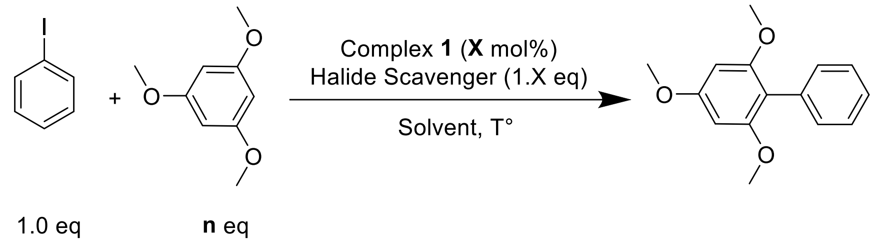

In a glovebox, a flame dried Schlenk equipped with a magnetic stirrer bar was charged with silver hexafluoroantimonate in 1.0 mL of solvent. Complex **1** was transferred into a small glass vial and dissolved in 1.0 mL of solvent. Aryl iodide and 1,3,5–trimethoxybenzene were added to the gold complex solution. This solution was loaded into a plastic syringe equipped with stainless steel needle. The syringe was closed by blocking the needle with a septum. Outside the glovebox, the Schlenk was cooled down to –10°C (Ethanol/N_2 cold bath). At this temperature, the solution of gold complex, aryl iodide and 1,3,5–trimethoxybenzene was added. The reaction mixture was then stirred at the appointed temperature. The yields were determined by GC-MS using *n*-dodecane as an internal standard.

General procedure for gold catalyzed arylation of 1,3,5-trimethoxybenzene with aryl iodides

In a glovebox, a flame dried Schlenk equipped with a magnetic stirrer bar was charged with silver hexafluoroantimonate (144 mg, 0.42 mmol) and potassium phosphate tribasic (85 mg, 0.40 mmol) in dichlorobenzene (2.0 mL). Complex **1** (13 mg, 0.02 mmol) was transferred into a small glass vial and dissolved in dichlorobenzene (2.0 mL). Aryl iodide (0.4 mmol, 1eq), 1,3,5-trimethoxybenzene (67.0 mg, 0.4 mmol) and methanol (80µL) were added to the gold complex solution. This solution was loaded into a plastic syringe equipped with stainless steel needle. The syringe was closed by blocking the needle with a septum. Outside the glovebox, the Schlenk was cooled down to -10°C (Ethanol/N_2 cold bath). At this temperature, the solution of complex **1**, aryl iodide and 1,3,5-trimethoxybenzene was added. The reaction mixture was then stirred at 75°C. After complete conversion, silver salts were filtrated, and the solvent evaporated. Isolated yields were determined after column chromatography (pentane/ethyl acetate). The fractions containing the biaryl product were then concentrated *in vacuo* to yield the pure product.

Procedure with aryl bromides:

The same procedure as for aryl iodides was followed, except that five equivalents of aryl bromides were used and 10 mol% of complex **1.**

Experimental procedure and characterization of biaryl products.

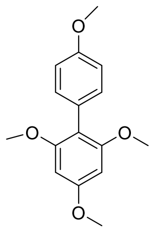

2,4,4’,6-tetramethoxy-1,1’-biphenyl: Compound **15** was prepared according to the general procedure described above using 4-iodoanisole with 1,3,5-trimethoxybenzene, and purified by column chromatography (pentane/ethyl acetate 100:0 to 95:5) to afford a white solid (70% isolated yield). Analytical data are consistent with that previously reported in the literature.[2] **^{1}H NMR** (300 MHz, $CDCl_3$): δ 7.29 (d, J_{HH} = 8.8 Hz, 2H), 6.93 (d, J_{HH} = 8.8 Hz, 2H), 6.23 (s, 2H), 3.86 (s, 3H), 3.83 (s, 3H), 3.72 (s, 6H).

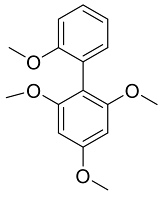

2,2’,4,6-tetramethoxy-1,1’-biphenyl: Compound **16** was prepared according to the general procedure described above using 2-iodoanisole with 1,3,5-trimethoxybenzene, and purified by column chromatography (pentane/ethyl acetate 100:0 to 95:5) to afford a white solid (82% isolated yield). Analytical data are consistent with that previously reported in the literature.[3] **^{1}H NMR** (300 MHz, $CDCl_3$): δ 7.37-7.32 (m, 1H), 7.21 (dd, J_{HH} = 7.46 Hz, J_{HH} = 1.66 Hz, 1H), 7.06-7.00 (m, 2H), 6.27 (s, 2H), 3.89 (s, 3H), 3.79 (s, 3H), 3.74 (s, 6H).

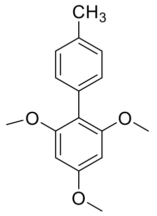

2,4,6-trimethoxy-4'-methyl-1,1'-biphenyl: Compound **17** was prepared according to the general procedure described above using 4-iodotoluene with 1,3,5-trimethoxybenzene, and purified by column chromatography (pentane/ethyl acetate 100:0 to 95:5) to afford a white solid (77% isolated yield). Analytical data are consistent with that previously reported in the literature.[2] **^{1}H NMR** (300 MHz, $CDCl_3$): δ 7.23-7.19 (m, 4H), 6.25 (s, 2H), 3.88 (s, 3H), 3.74 (s, 6H), 2.40 (s, 3H).

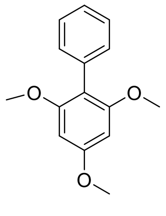

2,4,6-trimethoxy-1,1'-biphenyl: Compound **18** was prepared according to the general procedure described above using iodobenzene with 1,3,5-trimethoxybenzene, and purified by column chromatography (pentane/ethyl acetate 100:0 to 95:5) to yield a afford solid (75% isolated yield). Analytical data are consistent with that previously reported in the literature.[2] **^{1}H NMR** (300 MHz, $CDCl_3$): δ 7.46-7.33 (m, 5H), 6.27 (s, 2H), 3.90 (s, 3H), 3.75 (s, 6H).

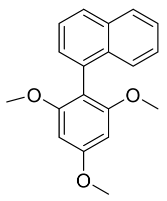

1-(2,4,6-trimethoxyphenyl)naphthalene: Compound **19** was prepared according to the general procedure described above using 1-iodonapthalene with 1,3,5-trimethoxybenzene, and purified by column chromatography (pentane/ethyl acetate 100:0 to 90:10) to afford a white solid (74% isolated yield). Analytical data are consistent with that previously reported in the literature.[2] **^{1}H NMR** (300 MHz, $CDCl_3$): δ 7.87 (t, J_{HH} = 8.34 Hz, 2H), 7.58-7.53 (m, 2H), 7.48-7.43 (m, 1H), 7.40-7.36 (m, 2H), 6.32 (s, 2H), 3.93 (s, 3H), 3.64 (s, 6H).

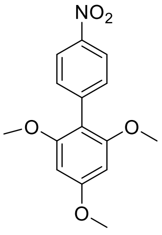

2,4,6-trimethoxy-4'-nitro-1,1'-biphenyl: Compound **20** was prepared according to the general procedure described above using 1-iodo-4-nitrobenzene with 1,3,5-trimethoxybenzene, and purified by column chromatography (pentane/ethyl acetate 100:0 to 90:10) to afford a yellow solid (78% isolated yield). Analytical data are consistent with that previously reported in the literature.[4] **^{1}H NMR** (300 MHz, $CDCl_3$): δ 8.22 (d, J_{HH} = 9.10 Hz, 2H), 7.51 (d, J_{HH} = 9.10 Hz, 2H), 6.23 (s, 2H), 3.88 (s, 3H), 3.74 (s, 6H).

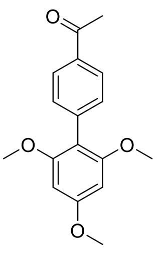

1-(2',4',6'-trimethoxybiphenyl-4-yl)ethanone: Compound **21** was prepared according to the general procedure described above using 4'-bromoacetophenone (5eq), 1,3,5-trimethoxybenzene (1eq) and 10 mol% of complex **1** (reaction time 12 h), and purified by column chromatography (pentane/ethyl acetate 100:0 to 90:10) to afford a yellow solid (40% isolated yield). Analytical data are consistent with that previously reported in the literature.[2] **^{1}H NMR** (300 MHz, $CDCl_3$): δ 7.98 (d, J_{HH} = 8.10 Hz, 2H), 7.45 (d, J_{HH} = 8.10 Hz, 2H), 6.23 (s, 2H), 3.87 (s, 3H), 3.73 (s, 6H), 2.62 (s, 3H).

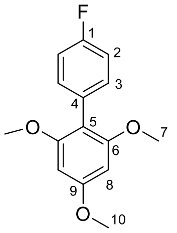

4'-fluoro-2,4,6-trimethoxy-1,1'-biphenyl: Compound **22** was prepared according to the general procedure described above using 1-fluoro-4-iodobenzene with 1,3,5-trimethoxybenzene, and purified by column chromatography (pentane/ethyl acetate 100:0 to 90:10) to afford a white solid (65% isolated yield). Analytical data are consistent with that previously reported in the literature.[5,6] **^{1}H NMR** (300 MHz, $CDCl_3$): δ 7.32-7.27 (m, 2H), 7.07 (t, J = 8.86 Hz, 2H), 6.23 (s, 2H), 3.87 (s, 3H), 3.73 (s, 6H). **^{13}C{^{1}H} NMR** (100 MHz, $CDCl_3$): δ 160.5 (d, J_{C-F} = 245.4 Hz, C_1), 160.7 (s, C_9), 158.5 (s, C_6), 132.8 (d, J_{C-F} = 7.8 Hz, C_3), 129.9 (d, J_{C-F} = 3.4 Hz, C_4), 114.7 (d, J_{C-F} = 21.5 Hz, C_2), 111.5 (s, C_5), 91.0 (s, C_8), 55.9 (s, C_7), 55.5 (s, C_{10}). **^{19}F{^{1}H} NMR** (282 MHz, $CDCl_3$): δ -116.5 (s).

Procedure for gold catalyzed direct arylation of 1-phenylpyrrole with iodobenzene

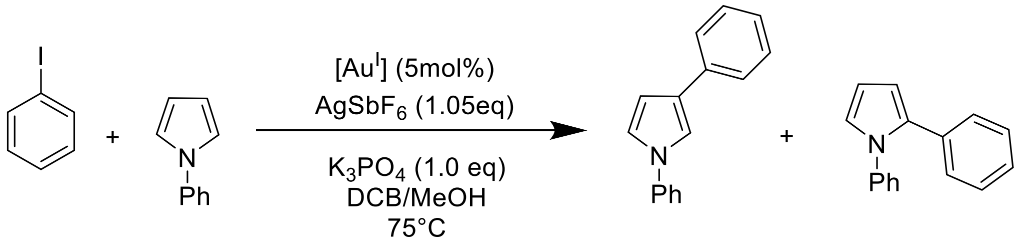

In a glovebox, a flame dried Schlenk equipped with a magnetic stirrer bar was charged with silver hexafluoroantimonate (289.0 mg, 0.84 mmol) and potassium phosphate tribasic (170.0 mg, 0.8 mmol) in dichlorobenzene (4.0 mL). Complex **1** (26.0 mg, 0.04 mmol) was transferred into a small glass vial and dissolved in dichlorobenzene (4.0 mL). Iodobenzene (90.0 µL, 0.8 mmol, 1eq), 1-phenyl-pyrrole (230.0 mg, 1.6 mmol) and methanol (160.0µL) were added to the gold complex solution. This solution was loaded into a plastic syringe equipped with stainless steel needle. The syringe was closed by blocking the needle with a septum. Outside the glovebox, the Schlenk was cooled down to –10°C (Ethanol/N_2 cold bath). At this temperature, the solution of complex **1**, iodobenzene and 1-phenyl-pyrrole was added. The reaction mixture was then stirred at 75°C (12h). After filtration of the silver salts, and a trap to trap to evaporate the dichlorobenzene, the sample was purified by column chromatography (pentane/ethyl acetate). The fractions containing the biaryl product were then concentrated *in vacuum* to give a mixture of α/β products in 61% yield. The two isomers were unambiguously identified by ^{13}C{^{1}H} NMR. Their relative proportion (1/9) was determined by ^{1}H NMR.

Experimental procedure and characterization of products.

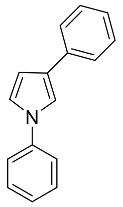

1,3-diphenyl-1*H*-pyrrole (β isomer): Analytical data are consistent with those previously reported in the literature.[7] **^{13}C{^{1}H} NMR** (125 MHz, $CDCl_3$): 140.6, 135.4, 129.7, 128.8, 127.0, 125.9, 125.8, 125.3, 120.6, 120.4, 115.9, 108.8.

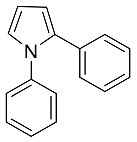

1,2-diphenyl-1H-pyrrole (α isomer): Analytical data are consistent with those previously reported in the literature.[8] **^{13}C{^{1}H} NMR** (125 MHz, $CDCl_3$): 140.8, 133.9, 133.1, 129.6, 128.4, 128.2, 126.7, 126.4,125.7, 124.5, 110.5, 109.4.

Mercury poisoning test

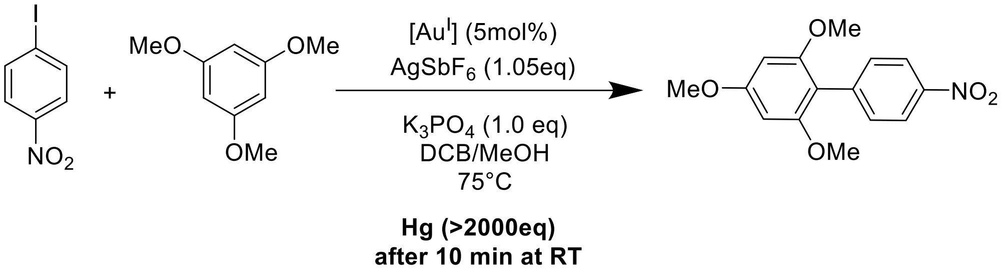

Two experiments with and without added Hg were carried out in parallel. In a glovebox, a flame dried Schlenk equipped with a magnetic stirrer bar was charged with silver hexafluoroantimonate (65.0 mg, 0.189 mmol) and potassium phosphate tribasic (38.0 mg, 0.180 mmol) in dichlorobenzene (1.0 mL). Complex **1** (6.0 mg, 0.009 mmol) was transferred into a small glass vial and dissolved in dichlorobenzene (1.0 mL). 1-Iodo-4-nitrobenzene (45.0 mg, 0.180 mmol), 1,3,5-trimethoxybenzene (30.0 mg, 0.18 mmol) and methanol (40 µL) were added to the gold complex solution. This solution was loaded into a plastic syringe equipped with stainless steel needle. The syringe was closed by blocking the needle with a septum. Outside the glovebox, the Schlenk was cooled down to – 10°C (Ethanol/N_2 cold bath). At this temperature, the solution of complex **1**, 1-iodo-4-nitrobenzene and 1,3,5-trimethoxybenzene was added. The reaction mixture was stirred at room temperature and after 10 minutes, one drop of Hg (>2000 equiv. relative to the catalyst) was added with a plastic Pasteur pipette. The reaction mixture was then stirred at 75°C. After the indicated times, aliquots were taken and analyzed by GC-MS. Yields were determined using *n*-dodecane as an internal standard.

Radical scavenger test

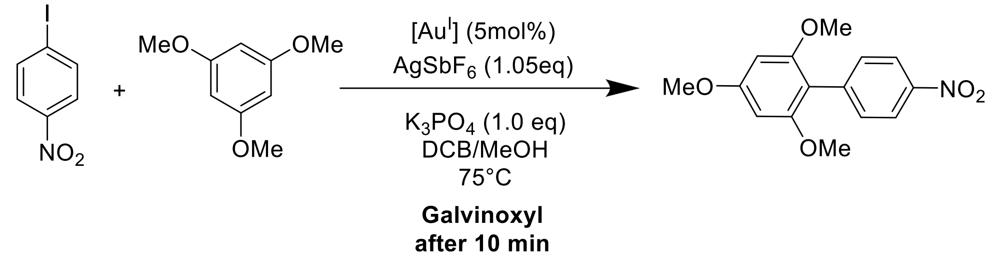

Two experiments with and without added galvinoxyl were carried out in parallel. In a glovebox, a flame dried Schlenk equipped with a magnetic stirrer bar was charged with silver hexafluoroantimonate (65.0 mg, 0.189 mmol) and potassium phosphate tribasic (38.0 mg, 0.180 mmol) in dichlorobenzene (1.0 mL). Complex **1** (6.0 mg, 0.009 mmol) was transferred into a small glass vial and dissolved in dichlorobenzene (1.0 mL). 1-Iodo-4-nitrobenzene (45.0 mg, 0.180 mmol), 1,3,5-trimethoxybenzene (30.0 mg, 0.180 mmol) and methanol (40 µL) were added to the gold complex solution. This solution was loaded into a plastic syringe equipped with stainless steel needle. The syringe was closed by blocking the needle with a septum. Outside the glovebox, the Schlenk was cooled down to – 10°C (Ethanol/N_2 cold bath). At this temperature, the solution of complex **1**, 1-iodo-4-nitrobenzene and 1,3,5-trimethoxybenzene was added. The reaction mixture was then stirred at 75°C. After 10 min, galvinoxyl (99.0 mg, 0.230 mmol) was added. After the indicated times, aliquots were taken and analyzed by GC-MS. Yields were determined using *n*-dodecane as an internal standard.

Influence of the day light

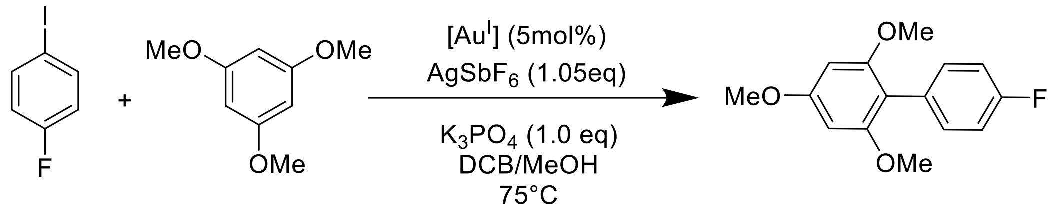

Two coupling experiments were carried out in parallel under exposure to day light and in the dark. In a glovebox, a flame dried Schlenk equipped with a magnetic stirrer bar was charged with silver hexafluoroantimonate (65.0 mg, 0.189 mmol) and potassium phosphate tribasic (38.0 mg, 0.180 mmol) in dichlorobenzene (1.0 mL). Complex **1** (6.0 mg, 0.009 mmol) was transferred into a small glass vial and dissolved in dichlorobenzene (1.0 mL). 1-Fluoro-4-iodobenzene (21.0 µL, 0.180 mmol), 1,3,5-trimethoxybenzene (30.0 mg, 0.180 mmol) and methanol (40 µL) were added to the gold complex solution. This solution was loaded into a plastic syringe equipped with stainless steel needle. The syringe was closed by blocking the needle with a septum. Outside the glovebox, the Schlenk was protected from light by wrapping it with aluminum foil and was cooled down to -10°C (Ethanol/N_2 cold bath). At this temperature, the solution of complex **1**, 1-fluoro-4-iodobenzene and 1,3,5-trimethoxybenzene was added. The reaction mixture was then stirred at 75°C for 2h. The yields was determined by GC-MS using *n*-dodecane as an internal standard. In both cases, the coupling product was formed in 95% yield.

Crystallographic data

Crystallographic data were collected at low temperature (193(2) K or 173(2) K) on a Bruker-AXS APEX II Quazar diffractometer equipped with a 30W air-cooled microfocus (**3**, **6**, **7** and **9**) or on a Bruker-AXS PHOTON100 D8 VENTURE diffractometer (**2** and **12**), using MoKα radiation (λ = 0.71073Å). Phi- and omega-scans were used. An empirical absorption correction was employed.[9] The structures were solved by direct methods (SHELXS-97),[10] and refined using the least-squares method on F^2. All non-H atoms were refined with anisotropic displacement parameters. Hydrogen atoms were refined isotropically at calculated positions using a riding model

Computational methods

DFT studies reported have been performed with B97D Grimme's functional including dispersion,[11] as implemented in Gaussian 09.[12] Gold, iodine or bromide and antimony atoms were described with the relativistic electron core potential SDD and the associated basis set.[13] The gold atom has been augmented by a set of f polarization functions.[14] 6-31G** basis set was employed for all other atoms. Full optimizations were carried out for all stationary points, *minima* and transition state structures, involved in the reaction process: oxidative addition of PhI or PhBr to real complex **1** at the B97D(SMD-DCM)/SDD+f(Au), SDD(I),6-31+G**(other atoms) level of theory by taking into account the counter-anion [SbF_6], **1-SbF_6**, and [NTf_2], **1-NTf_2** and solvent effects by means of the solvation model SMD[15] for dichloromethane (DCM, results reported in Figure 2, Supplementary Figure 49, 51 and 52); to the model compound **1H-SbF_6** at the B97D(SMD-DCM)/SDD+f(Au), SDD(I),6-31+G**(other atoms) level of theory taking into account solvent effect (Supplementary Figure 51); or to cationic species **1*** in gas phase at the B97D/SDD+f(Au), SDD(I), 6-31G**(other atoms) level of theory (Supplementary Figure 50). In each case, frequency calculations were undertaken at the same level of theory to confirm the nature of the stationary points, yielding one imaginary frequency for transition states (TS), corresponding to the expected process, and zero for *minima*. The connectivity of the transition states and their adjacent *minima* was confirmed by intrinsic reaction coordinate (IRC)[16,17] calculations. All reported electronic energies (ΔE) were zero-point energy (ZPE) corrected and Gibbs free energies (ΔG) temperature corrected using unscaled density functional frequencies. When counter-anion is taken into account upon optimization, all energies presented correspond to free energies in solution and are given in kcal·mol^{-1}. Without counter-anion, all energies presented correspond to free energies in gas phase.

Z-Matrices

1-NTf$_2$

C	-2.30984900	-0.69953800	1.82454100
C	-3.71218000	-0.86162100	1.90388400
H	-4.34532700	-0.45887600	1.12046400
C	-4.32403100	-1.55606800	2.95505500
H	-5.40850300	-1.66618200	2.97387000
C	-3.52561700	-2.11636800	3.96049300
H	-3.97873300	-2.67177300	4.78312000
C	-2.13785900	-1.93920000	3.92500900
H	-1.52891300	-2.34567600	4.73097600
C	-1.50363500	-1.22444700	2.88350400
C	0.70493500	-2.09037700	3.52975100
H	1.75592200	-1.92728700	3.25739300
H	0.38533000	-3.06996200	3.15106300
H	0.63631200	-2.09478500	4.63685400
C	0.30446500	0.29291700	3.41382900
H	0.12508900	0.37919100	4.50566100
H	-0.25882300	1.07650500	2.89667700
H	1.37278200	0.44522500	3.20859100
C	-2.27408500	1.72642500	0.00479800
C	-1.58385000	2.60253700	1.09485600
H	-1.91028000	2.26440700	2.09083200
H	-0.49135900	2.49030200	1.03914800
C	-1.94868900	4.09304600	0.89438300
H	-1.44274200	4.68334500	1.67434500
C	-3.47998400	4.27401500	1.00326800
H	-3.74435700	5.33560900	0.86759500
H	-3.82552100	3.96441700	2.00352800
C	-4.17202300	3.41596900	-0.08060100
H	-5.26515000	3.52127300	0.00129000
C	-3.70388800	3.87694700	-1.48029900
H	-4.20472200	3.28085400	-2.26127300
H	-3.97762200	4.93354500	-1.63473700
C	-2.17083700	3.70363000	-1.59187000
H	-1.82881600	4.01902300	-2.59015500
C	-1.46663900	4.54728700	-0.50367100
H	-0.37473000	4.42388900	-0.58150400
H	-1.69880900	5.61457200	-0.65034400
C	-3.81042400	1.92215300	0.14334900
H	-4.13134500	1.63551600	1.15410900
H	-4.34836800	1.29674900	-0.58208400
C	-1.82574300	2.20721400	-1.40338700
H	-2.35258900	1.62348100	-2.17127500
H	-0.74418100	2.04608600	-1.52892100
C	-2.14887100	-1.28353700	-1.10536600
C	-3.64741700	-1.24408200	-1.50232600
H	-4.28685100	-1.40456500	-0.62580600
H	-3.88945000	-0.26228800	-1.93493300
C	-3.95073500	-2.35865100	-2.53899500
H	-5.02316600	-2.31813900	-2.78665300
C	-3.60611700	-3.73836300	-1.92856100
H	-3.84345600	-4.53573600	-2.65178500
H	-4.21384200	-3.91115700	-1.02461500
C	-2.10157700	-3.78625700	-1.56897400
H	-1.85444600	-4.76470500	-1.12802300
C	-1.25525400	-3.55210400	-2.84082300
H	-0.18256900	-3.58601400	-2.58932800
H	-1.45774400	-4.34559700	-3.57872200
C	-1.60763300	-2.17072700	-3.43890000
H	-0.99165900	-1.98082000	-4.33180800
C	-1.29386300	-1.06984200	-2.39494100
H	-1.49268200	-0.08317700	-2.83040400
H	-0.22404500	-1.11877200	-2.14927400
C	-1.79290300	-2.68589900	-0.52484200
H	-2.37655000	-2.87685500	0.38672300
H	-0.72376700	-2.70548700	-0.25709400
C	-3.10637500	-2.12715900	-3.81140700
H	-3.35573800	-1.14845600	-4.25422000
H	-3.33216000	-2.90449600	-4.55974500
N	-0.09348900	-1.03181900	2.90631100
P	-1.62477300	-0.03532600	0.24186100
Au	0.64273000	0.04361500	0.15610500
N	2.79133600	0.09896200	-0.03448300
S	3.51175300	-1.15964400	-0.89280800
S	3.59386600	1.49975200	0.41781500
O	2.62061700	-1.56505800	-1.99815600
O	4.95541500	-0.99407600	-1.12801800
C	3.33616100	-2.53555900	0.42634200
O	2.87075100	2.06592900	1.57262600
O	5.06113500	1.39442200	0.43724000
C	3.15623500	2.62445200	-1.06367000
F	2.04904800	-2.91444300	0.54793200
F	4.07126000	-3.59104600	0.04028900
F	3.78122700	-2.08878700	1.61470800
F	1.84208300	2.92201300	-1.04956800
F	3.86811700	3.75980800	-0.98953700
F	3.44837300	1.98233500	-2.21035400

Sum of electronic and zero-point Energies= -3448.553169
Sum of electronic and thermal Free Energies= -3448.629916

TSexchange (1-NTf$_2$$\rightarrow$I-adduct[NTf$_2$])

C	-2.04693400	-1.45998700	2.07287900
C	-3.19699800	-2.24469300	2.32557000
H	-3.96024100	-2.33523600	1.56064900
C	-3.38522300	-2.93641200	3.52715100
H	-4.28495600	-3.53393800	3.67547400
C	-2.39985100	-2.85469600	4.51928700
H	-2.51846100	-3.38605700	5.46486900
C	-1.26192200	-2.07072400	4.30445600
H	-0.51697400	-1.99744100	5.09455300
C	-1.05635000	-1.35229500	3.10202300
C	1.30717900	-1.01858600	3.67458800
H	2.17475900	-0.48354600	3.27009900
H	1.44620400	-2.09586000	3.52663600
H	1.25295500	-0.80931200	4.76256100
C	-0.09424500	0.87706700	3.18416200
H	-0.28246100	1.08597200	4.25861500
H	-0.94397600	1.23725100	2.60006100
H	0.80869800	1.41734100	2.86855000
C	-3.27983800	0.48548300	0.12496100
C	-2.97239300	1.69295900	1.05471200
H	-3.00864900	1.36243500	2.10432900
H	-1.96662900	2.07875400	0.84563900
C	-4.00013500	2.82708500	0.83000200
H	-3.73938500	3.66615100	1.49293800
C	-5.42012800	2.31070200	1.15287700
H	-6.15770000	3.11516100	0.99679100
H	-5.47598100	1.99826400	2.20882000
C	-5.74191200	1.11092700	0.23323300
H	-6.74323900	0.71714400	0.46778400
C	-5.68807200	1.56135500	-1.24539400
H	-5.92689900	0.71227700	-1.90741300
H	-6.43918400	2.34854100	-1.42156500
C	-4.27399900	2.09779500	-1.56985100
H	-4.22641300	2.41687100	-2.62304000
C	-3.93301200	3.28749400	-0.64355800
H	-2.92369800	3.66292300	-0.86899300
H	-4.64528400	4.11208600	-0.81097000

C	-4.70634000	-0.01952600	0.48332400
H	-4.73726400	-0.29543600	1.54645100
H	-4.97722000	-0.90225500	-0.10597500
C	-3.24313200	0.96465900	-1.35305400
H	-3.48103900	0.12856400	-2.02424900
H	-2.23084900	1.32245500	-1.59904800
C	-2.02036300	-2.26116800	-0.85676300
C	-3.47178300	-2.71576800	-1.17291900
H	-4.00641100	-2.99781100	-0.25776500
H	-4.02214300	-1.89627000	-1.65414500
C	-3.45315800	-3.93474400	-2.13253400
H	-4.49458100	-4.24551200	-2.31212200
C	-2.66236100	-5.09773400	-1.48806000
H	-2.66718800	-5.96911500	-2.16291700
H	-3.14401300	-5.39944200	-0.54291100
C	-1.20816000	-4.64452900	-1.21921300
H	-0.63778100	-5.46348900	-0.75347900
C	-0.54082200	-4.23121200	-2.55163800
H	0.49506900	-3.91244700	-2.37055000
H	-0.52038600	-5.09220000	-3.24009700
C	-1.33892400	-3.06898700	-3.18359800
H	-0.85553800	-2.74879600	-4.11997400
C	-1.33197800	-1.86879600	-2.20369300
H	-1.83954800	-1.00709600	-2.65746900
H	-0.28603700	-1.58942200	-2.01768900
C	-1.22519500	-3.44386900	-0.23891100
H	-1.68579200	-3.75489500	0.70905300
H	-0.19965400	-3.11905000	-0.02745700
C	-2.78894700	-3.52097100	-3.46368800
H	-3.35951000	-2.69895000	-3.92755400
H	-2.78941100	-4.37059200	-4.16630900
N	0.11742800	-0.56090200	2.94538100
P	-1.88200100	-0.78435800	0.36361200
Au	0.07047600	0.31464700	-0.08844500
N	3.08002000	-0.25306100	-0.27112600
S	3.30355200	-1.65825800	-1.08691700
S	4.02327700	0.20836200	0.98426500
O	2.26128400	-1.67376600	-2.13975700
O	4.69780700	-2.00051600	-1.44704200
C	2.77111800	-3.03935600	0.14737200
O	3.26933400	1.22861600	1.74950200
O	4.70469800	-0.88370900	1.71892400
C	5.42860100	1.20728800	0.16604400
F	1.86212500	-2.56052800	1.02402300
F	2.21406400	-4.05814400	-0.54220500
F	3.82657100	-3.50852900	0.82736900
F	6.20965700	1.72921300	1.13424500
F	6.17450100	0.42053000	-0.62662200
F	4.91715600	2.21493900	-0.57116100
I	1.43194200	2.45842300	-1.42492000
C	0.08659300	3.95377100	-0.60928300
C	-0.21879800	3.88123200	0.75487100
C	-0.42237600	4.95657100	-1.44227100
C	-1.07245400	4.85537200	1.30008900
H	0.18193800	3.08103000	1.37427100
C	-1.27100400	5.92437100	-0.87486000
H	-0.17402500	4.98797100	-2.50207800
C	-1.59526600	5.87565100	0.48997800
H	-1.32651300	4.80573100	2.35933200
H	-1.67902200	6.71099500	-1.51053800
H	-2.25760800	6.62744000	0.91961100

Sum of electronic and zero-point Energies= -3691.385889
Sum of electronic and thermal Free Energies= -3691.472001

I-adduct[NTf_2]

C	0.45946800	3.68611800	-0.43875100
C	0.45098500	2.98974900	-1.65232700
H	0.92760100	2.01796400	-1.74361400
I	1.42914000	2.75120900	1.27657400
Au	0.00931600	0.35963400	0.64467100
C	-0.13394400	4.94270500	-0.28103300
H	-0.11906200	5.45834200	0.67788400
C	-0.75703600	5.52613900	-1.39985100
H	-1.22671000	6.50477300	-1.29505300
C	-0.78205200	4.85403500	-2.63175100
H	-1.27238400	5.31083100	-3.49164500
C	-0.18202000	3.59077600	-2.75424800
H	-0.20304900	3.05886200	-3.70614600
P	-1.86919700	-0.86717000	0.23025800
C	-2.53781500	-1.58052800	1.79558900
C	-3.18784100	0.32838800	-0.41190600
C	-1.49922500	-2.31323500	-0.94934300
C	-3.63778100	-2.46801700	1.71793800
C	-1.96844600	-1.31885900	3.07235000
C	-3.28064300	1.50089200	0.61172400
C	-4.61121900	-0.27784600	-0.55926400
C	-2.70182700	0.88372200	-1.77761400
C	-2.71820000	-2.83834900	-1.75584200
C	-0.41085300	-1.84362900	-1.96112000
C	-0.89284200	-3.46260300	-0.09377800
H	-4.08933300	-2.68661600	0.75478800
C	-4.16330800	-3.09076800	2.85565900
C	-2.50980800	-1.95527000	4.20921300
N	-0.87398500	-0.39976500	3.23752500
H	-3.62730000	1.10856600	1.58028700
H	-2.29025300	1.95563800	0.76048100
C	-4.26088600	2.58271900	0.09852500
C	-5.58867800	0.80386500	-1.09226700
H	-4.96337500	-0.62668300	0.42163100
H	-4.60525400	-1.13456300	-1.24211400
C	-3.68471000	1.96703000	-2.28250500
H	-2.64463900	0.06958300	-2.51436900
H	-1.69462800	1.31116000	-1.65934600
H	-3.52076500	-3.16678300	-1.08386700
H	-3.11461600	-2.03498100	-2.39261700
C	-2.28806000	-4.02697900	-2.65414500
C	0.00300700	-3.01309600	-2.88785900
H	-0.78870600	-1.00117400	-2.55582200
H	0.47703900	-1.50016600	-1.41558700
C	-0.46279200	-4.63351700	-1.01264300
H	-1.63437300	-3.82600900	0.63170400
H	-0.02346700	-3.08069000	0.46110500
H	-5.00834300	-3.77275600	2.75699400
C	-3.59166200	-2.83640300	4.11106400
H	-2.06949600	-1.74256400	5.18331000
C	0.25744700	-0.93625500	4.00390300
C	-1.31594100	0.90167200	3.75713800
H	-4.29725600	3.39946600	0.83692600
C	-5.66729300	1.96760400	-0.07829500
C	-3.74997500	3.12270200	-1.25723700
H	-6.58194200	0.34381100	-1.21279900
C	-5.08873000	1.33919100	-2.45567600
H	-3.31999300	2.34763800	-3.24937200
H	-3.17656600	-4.39373400	-3.19206900
C	-1.69985700	-5.15963400	-1.77917800
C	-1.22459300	-3.53898000	-3.66253400
C	0.60092700	-4.14748000	-2.02353100
H	0.76047200	-2.64184800	-3.59100800
H	-0.04460100	-5.43417500	-0.38269000
H	-3.99027100	-3.31357400	5.00754000
H	1.08185200	-0.21456700	3.93813100
H	0.58577200	-1.88131500	3.55531700
H	0.02371300	-1.09889200	5.07554300
H	-1.72098600	0.82902000	4.78770900
H	-2.08614500	1.31735600	3.09658700
H	-0.45558000	1.58614400	3.76075000
H	-6.37004000	2.73545500	-0.44121100
H	-6.04009100	1.59640200	0.89067900
H	-2.75075200	3.56536700	-1.13243400
H	-4.42633400	3.91179600	-1.62529500

H	-5.04462500	0.51767400	-3.19013900
H	-5.79359200	2.09538800	-2.83850300
H	-1.41148800	-6.01079800	-2.41778800
H	-2.46099300	-5.51553300	-1.06482300
H	-1.64515500	-2.73785900	-4.29348800
H	-0.92416400	-4.36778900	-4.32459600
H	1.49031200	-3.78015300	-1.48917400
H	0.91507400	-4.98539000	-2.66745700
N	2.83229400	-0.40981900	-0.30978500
S	3.80518100	0.24172800	-1.45985000
S	3.30972100	-1.61312700	0.68537500
O	3.48328400	1.68160400	-1.57737300
O	5.22354000	-0.17952300	-1.46691400
C	3.05262000	-0.51850700	-3.03217800
O	2.09358700	-2.16365700	1.32627500
O	4.33409400	-2.54967200	0.17333200
C	4.17349200	-0.65584900	2.09266200
F	1.77614700	-0.09768100	-3.19214400
F	3.76797700	-0.13282400	-4.10656500
F	3.06295100	-1.86373000	-2.95610500
F	4.68921900	-1.52475700	2.98320200
F	5.16237200	0.11602500	1.60292300
F	3.28688500	0.14298800	2.73578300

Sum of electronic and zero-point Energies= -3691.392624
Sum of electronic and thermal Free Energies= -3691.484103

TS$_1$[NTf$_2$]

C	-0.13459700	-1.63019300	-2.22746000
C	-0.91813700	-0.60221300	-2.76194500
H	-1.76539700	-0.21147000	-2.21152500
I	-1.22003300	-3.13066700	-0.63573400
Au	0.41313100	-1.05265500	0.11212500
C	0.94170400	-2.21142100	-2.91099500
H	1.51224300	-3.02866800	-2.47587800
C	1.27801800	-1.68105600	-4.16898300
H	2.12374600	-2.10820800	-4.70878800
C	0.54518100	-0.61579000	-4.71633100
H	0.81491200	-0.21458100	-5.69351600
C	-0.54729200	-0.08045600	-4.01190100
H	-1.13087200	0.74147000	-4.42853700
P	1.99128600	0.57371400	0.59314800
C	2.52053800	0.32916900	2.33856600
C	3.51552400	0.24302400	-0.46662600
C	1.29488400	2.33500200	0.42747200
C	3.40154600	1.26103100	2.93402900
C	2.03930600	-0.75109800	3.12263000
C	3.86919200	-1.26558100	-0.29506700
C	4.77055000	1.07395200	-0.08432500
C	3.13123100	0.50019100	-1.94875400
C	2.35396500	3.42715100	0.11083300
C	0.23547900	2.30854300	-0.71749200
C	0.57561500	2.70248200	1.75693700
H	3.77046300	2.09991500	2.34864400
C	3.79837700	1.13689000	4.27042300
C	2.44567200	-0.86522500	4.46677800
N	1.15778300	-1.75428700	2.56515600
H	4.12988500	-1.46178900	0.75612200
H	2.99957000	-1.88919300	-0.55243000
C	5.05538600	-1.64343800	-1.21624000
C	5.94628600	0.70553000	-1.02747400
H	5.05813600	0.85294300	0.95328100
H	4.56319500	2.14665400	-0.16088100
C	4.31591700	0.11903300	-2.86938300
H	2.87894600	1.55884800	-2.09984700
H	2.24679700	-0.09636300	-2.20460200
H	3.10843300	3.46646000	0.90882900
H	2.86680800	3.20044700	-0.83321400
C	1.66513500	4.81132600	-0.01266400
C	-0.43679500	3.69656000	-0.85066400
H	0.71486200	2.02466800	-1.66529000
H	-0.53422900	1.55799900	-0.49044400
C	-0.11426700	4.08390600	1.62098600
H	1.30526700	2.74597100	2.57721300
H	-0.17034100	1.93541600	1.99845200
H	4.47522800	1.87294900	4.70494600
C	3.31539500	0.06858500	5.04171300
H	2.07463600	-1.70246800	5.05762100
C	-0.08389000	-1.95422100	3.33509000
C	1.85148400	-3.03604600	2.33125900
H	5.28074400	-2.71243500	-1.07583500
C	6.29138200	-0.79085900	-0.85253100
C	4.65896400	-1.37838600	-2.68753100
H	6.81599100	1.32419500	-0.75618900
C	5.54592000	0.98093900	-2.49742300
H	4.02226800	0.30951100	-3.91346200
H	2.43911000	5.56376100	-0.23206500
C	0.95312700	5.15998900	1.31478800
C	0.63232300	4.76708300	-1.16326100
C	-1.15127200	4.04221500	0.47581300
H	-1.17204800	3.64898500	-1.66530500
H	-0.61789300	4.31374600	2.57308000
H	3.61595500	-0.03913200	6.08469200
H	-0.74580600	-2.59569100	2.73835200
H	-0.57490300	-0.98716100	3.48495000
H	0.10120800	-2.44163200	4.31057000
H	2.15718100	-3.51354200	3.28178500
H	2.73722900	-2.86323800	1.70822800
H	1.16215800	-3.70514300	1.79616500
H	7.13762100	-1.06191100	-1.50484000
H	6.59052900	-0.98661700	0.19042800
H	3.78917000	-1.99577400	-2.95734000
H	5.49256400	-1.65429200	-3.35392700
H	5.30921100	2.04998700	-2.62778900
H	6.38977700	0.73889200	-3.16411300
H	0.47596100	6.15036000	1.23358600
H	1.68843600	5.20420000	2.13543800
H	1.14019700	4.52739700	-2.11266100
H	0.15376600	5.75374300	-1.27512000
H	-1.92149000	3.28967200	0.69502800
H	-1.65184100	5.02052000	0.38658800
N	-3.23141600	0.09914700	-0.12163600
S	-4.47449600	0.36755200	-1.16387000
S	-3.33402500	0.48216900	1.46754100
O	-4.29113300	-0.56294700	-2.30100700
O	-5.82411000	0.54937100	-0.58525600
C	-4.02770200	2.06978600	-1.89971800
O	-1.95133300	0.49124500	1.99877500
O	-4.24952000	1.58377100	1.84121800
C	-4.09844400	-1.07839500	2.25861000
F	-2.83287200	2.00786700	-2.53464100
F	-4.96922700	2.43185000	-2.79345500
F	-3.95531200	3.00583700	-0.93579900
F	-4.27710500	-0.85603200	3.57754400
F	-5.28416300	-1.37071300	1.69535300
F	-3.27420100	-2.13871000	2.10991000

Sum of electronic and zero-point Energies= -3691.375350
Sum of electronic and thermal Free Energies= -3691.462043

2-NTf$_2$

C	0.40765900	-0.31340900	-2.19623600
C	-0.76302800	0.40569900	-2.46947700
H	-1.52283200	0.53700100	-1.70538900
I	-1.01050700	-3.14872000	-1.36381100
Au	0.65922800	-1.17381400	-0.31741900
C	1.33446700	-0.58574000	-3.21311300
H	2.22214900	-1.18286300	-3.02004900
C	1.11769700	-0.06391000	-4.50235100

H	1.84964200	-0.26533400	-5.28635800
C	-0.02618200	0.70157500	-4.77410800
H	-0.19131200	1.10363900	-5.77468600
C	-0.96994300	0.92394300	-3.75872600
H	-1.88384700	1.48311600	-3.95712200
P	2.12904700	0.39318400	0.66338400
C	2.49496200	-0.37865400	2.28827800
C	3.74690300	0.49737900	-0.28996000
C	1.27478400	2.03077800	1.02726400
C	3.31868100	0.27888300	3.23149600
C	1.89655300	-1.60112600	2.64139200
C	4.12080000	-0.95706000	-0.70466600
C	4.92196000	1.06347500	0.56098500
C	3.55452600	1.39043600	-1.54669700
C	2.26471500	3.16180100	1.42273500
C	0.45699500	2.46010200	-0.21810600
C	0.28057900	1.77201400	2.19421300
H	3.77656400	1.23120600	2.98423000
C	3.55429400	-0.28052700	4.49099100
C	2.14478600	-2.16589500	3.90549600
N	1.01125900	-2.31494100	1.69969500
H	4.27149700	-1.56466700	0.20172500
H	3.30972000	-1.41464400	-1.28482800
C	5.41510100	-0.94188700	-1.55359100
C	6.21716200	1.08930100	-0.29437800
H	5.09508000	0.41634300	1.43001600
H	4.68788300	2.07465600	0.91764900
C	4.85044300	1.37745400	-2.39400800
H	3.33492100	2.42065200	-1.23538700
H	2.71139200	1.03154300	-2.14503200
H	2.84098800	2.87673300	2.31245000
H	2.96827000	3.35855900	0.60222100
C	1.46118500	4.45246800	1.73637200
C	-0.31352800	3.76794900	0.08237300
H	1.10923100	2.58602200	-1.09239600
H	-0.26607100	1.67126400	-0.44038800
C	-0.51262300	3.06844800	2.49599400
H	0.82653300	1.46383700	3.09662200
H	-0.41401900	0.96761300	1.91524600
H	4.19102500	0.24318400	5.20371200
C	2.96820400	-1.51005100	4.82625900
H	1.69353800	-3.11962300	4.17103000
C	-0.33889200	-2.53494100	2.31938600
C	1.63556900	-3.63117100	1.33803100
H	5.64997600	-1.97613700	-1.84881700
C	6.57438500	-0.35422200	-0.71684300
C	5.18954800	-0.07283100	-2.81419900
H	7.02445100	1.50916300	0.32548800
C	6.00789900	1.96260400	-1.55328500
H	4.68359800	1.99577400	-3.28945800
H	2.17734200	5.23823100	2.02337600
C	0.47913800	4.18481400	2.90135800
C	0.67689600	4.88627100	0.47771500
C	-1.30139500	3.50055100	1.24032200
H	-0.86779100	4.05612600	-0.82154400
H	-1.20871100	2.85953000	3.32174500
H	3.14638900	-1.95980400	5.80301300
H	-0.94683700	-3.07974200	1.59439900
H	-0.79360600	-1.56119600	2.52698500
H	-0.25021200	-3.12371200	3.24320800
H	1.77762300	-4.24439900	2.23968300
H	2.60105300	-3.43778400	0.85585900
H	0.96699900	-4.14578300	0.64160400
H	7.50222100	-0.35261500	-1.31109400
H	6.74384200	-0.97613800	0.17751800
H	4.36808000	-0.49069500	-3.41709700
H	6.10039900	-0.07679500	-3.43406900
H	5.77029000	2.99830800	-1.25952000
H	6.93469700	1.98455500	-2.14912000
H	-0.07314900	5.10808700	3.13978600
H	1.03903600	3.88357800	3.80238100
H	1.37731400	5.08261800	-0.35138200
H	0.12679800	5.81884500	0.68331800
H	-2.00035700	2.70392600	0.95215600
H	-1.88698200	4.40874000	1.45566400
N	-3.42067300	0.32813700	0.00728000
S	-4.50809200	0.76576900	-1.15052000
S	-3.85755800	0.17074500	1.57890200
O	-4.05949300	0.17474600	-2.43079600
O	-5.94298300	0.71175800	-0.79279900
C	-4.12416500	2.62136100	-1.34045400
O	-2.60868000	0.17162200	2.37847400
O	-5.00565700	0.97469700	2.05316000
C	-4.45119000	-1.63906400	1.66625300
F	-2.84668800	2.79591000	-1.75242600
F	-4.94927200	3.15997700	-2.26052600
F	-4.29327000	3.26504100	-0.16951300
F	-4.93113600	-1.89513600	2.90126700
F	-5.42083700	-1.86677100	0.76134200
F	-3.42207900	-2.47739200	1.42012600

Sum of electronic and zero-point Energies= -3691.406375
Sum of electronic and thermal Free Energies= -3691.493329

1-SbF$_6$

C	-1.80714600	0.05185800	2.06399300
C	-3.11700500	0.25800000	2.55696500
H	-3.92878300	0.46526300	1.86629500
C	-3.39953200	0.20978100	3.92698100
H	-4.42099300	0.37079100	4.27289200
C	-2.36625400	-0.04287200	4.84188900
H	-2.57335500	-0.08005500	5.91231100
C	-1.06286200	-0.23909000	4.37254800
H	-0.24940100	-0.42325500	5.07468900
C	-0.76119800	-0.19533100	2.99628600
C	1.17916200	-1.67423600	2.97063500
H	2.16734900	-1.76171400	2.49861600
H	0.53672100	-2.48706800	2.60446500
H	1.30104400	-1.77668900	4.06763300
C	1.47422100	0.74461200	2.93259600
H	1.61310900	0.83581500	4.02938700
H	1.04138800	1.67802600	2.55082500
H	2.45130700	0.58786300	2.45923900
C	-1.74925300	1.87106300	-0.30627500
C	-0.58406000	2.68768100	0.33172800
H	-0.66292000	2.63926200	1.42875500
H	0.38415500	2.25559300	0.04026200
C	-0.64373200	4.15816400	-0.14756900
H	0.19076600	4.70861400	0.31393100
C	-1.99110800	4.78953900	0.27122200
H	-2.03523300	5.83846000	-0.06500500
H	-2.08520700	4.78142200	1.36971300
C	-3.14904700	3.98561100	-0.36279000
H	-4.11529300	4.41616600	-0.05684300
C	-3.02245300	4.02273200	-1.90427000
H	-3.85426600	3.46318400	-2.36378600
H	-3.08144800	5.06532600	-2.25629800
C	-1.67104300	3.39900800	-2.32868100
H	-1.57801900	3.41700400	-3.42562400
C	-0.50690800	4.19141500	-1.68845000
H	0.45675200	3.74659700	-1.98666200
H	-0.52472100	5.23481400	-2.04287500
C	-3.09093700	2.51699400	0.13690800
H	-3.16064600	2.50644000	1.23362000
H	-3.94341100	1.95687800	-0.26627000
C	-1.62359600	1.92640700	-1.85246800
H	-2.45296700	1.36935300	-2.31239700
H	-0.67693100	1.45246800	-2.15979900
C	-2.70214400	-1.15910600	-0.57217300
C	-4.15881500	-0.65136700	-0.74972400
H	-4.59185300	-0.35805800	0.21485000

H	-4.16278100	0.22612300	-1.41115600
C	-5.03323300	-1.77040400	-1.37295600
H	-6.06368500	-1.39330700	-1.46558700
C	-5.01151600	-3.01612200	-0.45546500
H	-5.65155900	-3.80498300	-0.88298800
H	-5.41339500	-2.75697800	0.53824100
C	-3.56016500	-3.53682400	-0.31674100
H	-3.54229000	-4.42109900	0.33895300
C	-3.00818800	-3.90429400	-1.71361900
H	-1.97599500	-4.28086900	-1.62262700
H	-3.62234000	-4.70305600	-2.16042800
C	-3.02971500	-2.65046500	-2.61796000
H	-2.61311600	-2.89528400	-3.60740900
C	-2.14542000	-1.55212800	-1.97842800
H	-2.10392900	-0.67216200	-2.63310500
H	-1.12085500	-1.93958000	-1.87541300
C	-2.68345700	-2.43186900	0.32266700
H	-3.06797400	-2.19521900	1.32487400
H	-1.64225300	-2.78063100	0.42929200
C	-4.47904900	-2.13675100	-2.76770000
H	-4.49844000	-1.25071100	-3.42381500
H	-5.10838600	-2.91421500	-3.23063100
N	0.60170900	-0.38257400	2.56995600
P	-1.52219800	0.08452500	0.24103300
Au	0.57851100	-0.55789200	-0.18416400
F	2.60115100	-1.39232700	-0.65674900
Sb	4.22477300	-0.24554400	-0.47451600
F	3.95180800	0.20521500	-2.30297200
F	5.21054300	-1.79332200	-0.95602300
F	5.78041600	0.82002600	-0.30261300
F	3.10857000	1.23969100	0.00188600
F	4.24326200	-0.77108000	1.35970400

Sum of electronic and zero-point Energies= -2226.310974
Sum of electronic and thermal Free Energies= -2226.382497

PhI in solvent (DCM)

C	-2.68505200	-1.21458600	0.00000100
C	-1.27893400	-1.22626600	-0.00000300
C	-0.60104200	-0.00001800	-0.00000100
C	-1.27892000	1.22625200	0.00000000
C	-2.68501800	1.21460600	-0.00000300
C	-3.38875000	0.00000900	0.00000300
H	-3.22256000	-2.16384000	0.00001000
H	-0.73433200	-2.16899500	-0.00001000
H	-0.73427100	2.16895500	-0.00000300
H	-3.22254400	2.16385000	0.00000700
H	-4.47919300	0.00004100	0.00000300
I	1.58300400	0.00000000	0.00000000

Sum of electronic and zero-point Energies= -242.840293
Sum of electronic and thermal Free Energies= -242.872205

TS_exchange [1-SbF_6→1-PhI/SbF_6]

C	-2.15794800	0.57811200	2.06481100
C	-3.46723900	0.88926900	2.50227600
H	-4.23788800	1.12773700	1.77503600
C	-3.80190100	0.90306800	3.86111700
H	-4.82071900	1.14690900	4.16368000
C	-2.82247500	0.60444500	4.82073300
H	-3.06956600	0.61088000	5.88335300
C	-1.52038300	0.30725000	4.40509200
H	-0.74684700	0.08614800	5.14104900
C	-1.16393300	0.29755800	3.04058400
C	0.62163500	-1.34308700	3.01327400
H	1.64106300	-1.48537200	2.63566200
H	-0.03913200	-2.06076200	2.51170800
H	0.61234200	-1.54041000	4.10517300
C	1.13995300	1.02967300	3.19220700
H	1.23243400	0.99596600	4.29778600
H	0.80489200	2.03069600	2.89454700
H	2.12120500	0.84415600	2.74334800
C	-1.77311500	2.37584200	-0.26574400
C	-0.57969300	3.02967400	0.49268100
H	-0.76777900	2.98623000	1.57637800
H	0.34990800	2.48517500	0.29003200
C	-0.40881200	4.50019000	0.04444000
H	0.44929300	4.92906700	0.58482400
C	-1.69521300	5.29315100	0.36901100
H	-1.58002900	6.34466300	0.05839900
H	-1.88089900	5.27923100	1.45581200
C	-2.88813400	4.65113900	-0.37509200
H	-3.81596900	5.19568500	-0.13831100
C	-2.63110300	4.69260900	-1.90053800
H	-3.48520300	4.24717000	-2.43781900
H	-2.53348300	5.73901100	-2.23310300
C	-1.33667400	3.91181200	-2.23188100
H	-1.15107700	3.93784000	-3.31707600
C	-0.13924300	4.53814500	-1.47873700
H	0.78166400	3.97746300	-1.70708700
H	0.00579000	5.58009700	-1.80805600
C	-3.05717400	3.17720900	0.08436100
H	-3.22200500	3.15103600	1.17117600
H	-3.93733200	2.74528400	-0.40479300
C	-1.50275500	2.43682400	-1.79314900
H	-2.33810200	1.97698600	-2.34155600
H	-0.58718400	1.86951800	-2.02462500
C	-3.12380400	-0.49505700	-0.61133100
C	-4.44195000	0.25731700	-0.94319400
H	-4.89475100	0.68021200	-0.03762800
H	-4.22966400	1.08526600	-1.63398000
C	-5.45112300	-0.71260300	-1.61035100
H	-6.38263700	-0.15968200	-1.80854300
C	-5.73843600	-1.90093700	-0.66243700
H	-6.47357100	-2.57839900	-1.12630700
H	-6.16939200	-1.53071900	0.28249500
C	-4.42393900	-2.66777400	-0.37674500
H	-4.62477500	-3.51303600	0.30040500
C	-3.83201900	-3.18591600	-1.70732800
H	-2.90255300	-3.74571600	-1.51609900
H	-4.54464700	-3.87431000	-2.19030400
C	-3.54213700	-1.98835100	-2.64110200
H	-3.09253700	-2.34694900	-3.58026100
C	-2.53160100	-1.04013100	-1.94955000
H	-2.27096900	-0.20539900	-2.61554800
H	-1.61108600	-1.60321400	-1.74144900
C	-3.42046900	-1.70989400	0.31340000
H	-3.84819700	-1.36839700	1.26566400
H	-2.47614900	-2.23005000	0.53522300
C	-4.85568300	-1.23218100	-2.93821700
H	-4.65799600	-0.38580200	-3.61673900
H	-5.57344200	-1.90466600	-3.43563900
N	0.20099100	0.02430900	2.67437300
P	-1.80162200	0.56716400	0.25662200
Au	0.24110600	-0.30352400	-0.16672700
I	1.89445100	-2.52311300	-1.69991600
C	1.15267200	-3.58540300	0.05106600
C	-0.22847100	-3.78449200	0.16513200
C	2.05465400	-3.99518400	1.03774500
C	-0.71838900	-4.43508800	1.31008500
H	-0.90800100	-3.43283200	-0.60692100
C	1.54360000	-4.64590600	2.17565700
H	3.12180800	-3.80524900	0.93643900
C	0.16438500	-4.86790900	2.31275200
H	-1.79272100	-4.59291100	1.41144000
H	2.23426800	-4.97163400	2.95426500
H	-0.22175500	-5.37018700	3.20001400
F	2.89823000	-0.29919900	0.77393500
Sb	3.79199300	1.16258000	-0.13317100
F	2.66572400	2.40835400	0.78124300
F	2.52133800	1.14187200	-1.58562700
F	4.70508800	2.58905400	-1.00179000
F	4.82938300	-0.13736100	-1.06633800
F	5.02105900	1.19585700	1.32197000

Sum of electronic and zero-point Energies= -2469.172434
Sum of electronic and thermal Free Energies= -2469.252657

I-adduct[SbF_6]

C	0.29612800	-3.50185300	-0.93246700
C	0.04394100	-2.72867500	-2.07077800
H	-0.80326600	-2.04929000	-2.10903000
I	-1.04055200	-3.23938400	0.77546800
Au	-0.19309500	-0.52470900	0.66608000
C	1.39302700	-4.36099700	-0.82384900
H	1.57078400	-4.94149400	0.07969800
C	2.27716600	-4.44328200	-1.91592000
H	3.14308400	-5.10253300	-1.84804900
C	2.05447000	-3.67948100	-3.07194700
H	2.75031100	-3.74412300	-3.90881800
C	0.94086000	-2.82725100	-3.14775500
H	0.76313400	-2.22535700	-4.03963800
P	1.35477900	1.08780800	0.25382100
C	1.37350400	2.35528500	1.58571900
C	3.04282100	0.22655400	0.27874600
C	0.97319300	1.97799100	-1.39566000
C	2.20511200	3.48713400	1.41744900
C	0.51734200	2.29766200	2.72388800
C	3.15596200	-0.53266100	1.63481400
C	4.26377000	1.18133600	0.17192100
C	3.06931200	-0.81625400	-0.87362200
C	2.21300500	2.54845600	-2.13587700
C	0.27032800	0.95033300	-2.33677400
C	-0.02362000	3.13364100	-1.09752300
H	2.85141800	3.55128000	0.54585700
C	2.19701600	4.54832000	2.32993800
C	0.51726500	3.38055900	3.62890900
N	-0.32873200	1.16447100	2.97337500
H	3.13539700	0.19305100	2.46242200
H	2.30262400	-1.21763600	1.75574400
C	4.47120400	-1.34800900	1.68467700
C	5.57944500	0.35917600	0.20025000
H	4.25734900	1.87811400	1.02211100
H	4.22517200	1.76959500	-0.75065800
C	4.38971100	-1.62129100	-0.82354200
H	2.98257100	-0.31138900	-1.84582700
H	2.21321800	-1.49871600	-0.77091900
H	2.74292800	3.27542300	-1.50479400
H	2.91004700	1.73470300	-2.37898400
C	1.77142400	3.24228200	-3.45234200
C	-0.15337600	1.63123300	-3.65933200
H	0.94147400	0.10365800	-2.53563800
H	-0.63189200	0.55688000	-1.85539600
C	-0.46390100	3.80667900	-2.42159600
H	0.45235300	3.88432000	-0.45181700
H	-0.90266400	2.73822700	-0.57474700
H	2.84539200	5.40953600	2.16614500
C	1.34012000	4.49654100	3.43930700
H	-0.13619100	3.33352200	4.49938900
C	-1.70129000	1.48845100	3.38386500
C	0.28774000	0.19038400	3.88744300
H	4.51783400	-1.87492000	2.65088100
C	5.68099000	-0.39662700	1.54400800
C	4.47914300	-2.37229100	0.52500400
H	6.42558000	1.05646900	0.09703300
C	5.58790800	-0.65519400	-0.96867200
H	4.38513300	-2.34469400	-1.65265400
H	2.66869400	3.65009000	-3.94460900
C	0.77982200	4.38568900	-3.13623700
C	1.08863600	2.20692000	-4.37497400
C	-1.15414600	2.76555300	-3.33333400
H	-0.64081000	0.87511500	-4.29536100
H	-1.16856900	4.61780700	-2.18024200
H	1.31773100	5.31620600	4.15912100
H	-2.29815700	0.56883700	3.32166700
H	-2.12503800	2.22448300	2.69211800
H	-1.76597000	1.87786900	4.42005600
H	0.40364800	0.59638300	4.91388100
H	1.27065700	-0.10292900	3.50248900
H	-0.35275800	-0.70321600	3.92751300
H	6.61931700	-0.97418600	1.58333300
H	5.69091400	0.32417100	2.37825800
H	3.62279600	-3.05891700	0.62310400
H	5.40329800	-2.97203700	0.56189100
H	5.52171500	-0.12015800	-1.93089000
H	6.53294500	-1.22281700	-0.96231400
H	0.47897000	4.88581000	-4.07162200
H	1.26516000	5.13809800	-2.49211400
H	1.79582400	1.39465000	-4.61395300
H	0.79289300	2.68702900	-5.32219400
H	-2.03858500	2.34904000	-2.82529900
H	-1.49200300	3.24926800	-4.26481800
Sb	-3.89753800	0.24475900	-0.20323500
F	-2.62511100	1.59215800	0.29156800
F	-4.35108600	1.29138300	-1.73428700
F	-2.59882600	-0.66064700	-1.30236000
F	-5.14148600	-1.12043200	-0.67207200
F	-3.39010600	-0.77824500	1.34792500
F	-5.21239200	1.13745800	0.84802300

Sum of electronic and zero-point Energies= -2469.179406
Sum of electronic and thermal Free Energies= -2469.259530

TS_1[SbF_6]

C	0.16455200	-2.50340600	-1.61297900
C	-0.52556400	-1.97741200	-2.71189500
H	-1.52871900	-1.57684400	-2.59999900
I	-1.19699200	-3.43372200	0.18269900
Au	-0.02545400	-0.94993100	0.32287000
C	1.45072800	-3.05128700	-1.70293300
H	1.94594300	-3.46779300	-0.82919100
C	2.09496900	-3.00528600	-2.95098000
H	3.10644600	-3.40473200	-3.03401100
C	1.45329700	-2.44414300	-4.06710900
H	1.96109100	-2.41574000	-5.03134900
C	0.14979100	-1.93312300	-3.94347600
H	-0.35872500	-1.49718700	-4.80471700
P	1.41310800	0.85074600	0.43428700
C	1.30082700	1.47994500	2.16527800
C	3.20366800	0.29261400	0.17429500
C	0.89500800	2.21057700	-0.78717200
C	1.91623000	2.71114000	2.48746900
C	0.58268600	0.79138400	3.18596700
C	3.42490800	-0.99967700	1.01192600
C	4.25497300	1.33861800	0.63679900
C	3.41766400	-0.02962300	-1.32901300
C	1.96497900	3.29978800	-1.08283500
C	0.49948000	1.50890600	-2.12608700
C	-0.38068600	2.88641200	-0.21361900
H	2.48410600	3.23905900	1.72725700
C	1.81703800	3.27180000	3.76586400
C	0.49451600	1.36759500	4.46913400
N	-0.04650100	-0.49139100	2.95260600
H	3.31211500	-0.75030600	2.07671900
H	2.67472800	-1.76133800	0.74901800
C	4.84780400	-1.56162900	0.77638500
C	5.68484200	0.78547000	0.38720100
H	4.12791800	1.53903400	1.71031500
H	4.12216000	2.28361100	0.09651300
C	4.84013600	-0.59607900	-1.55259000
H	3.31566100	0.88523200	-1.92587200
H	2.66216500	-0.74817900	-1.66992000
H	2.25081700	3.82360800	-0.16118600
H	2.86867300	2.84639700	-1.51041500
C	1.39834700	4.33594300	-2.08953300
C	-0.04176200	2.55060700	-3.13519800
H	1.35743600	0.98665400	-2.56390500
H	-0.28295700	0.76755100	-1.93105600
C	-0.93295800	3.92562600	-1.22066500
H	-0.14824400	3.38024800	0.74173000

H	-1.14574900	2.12735200	-0.02830200
H	2.29909300	4.22654200	3.97755200
C	1.09630500	2.59701100	4.76115400
H	-0.05100300	0.83475100	5.24689500
C	-1.40910400	-0.62361700	3.50669200
C	0.77876500	-1.62187500	3.42631800
H	4.96626100	-2.47743700	1.37675000
C	5.88924000	-0.50584300	1.21270300
C	5.02909200	-1.88651200	-0.72382300
H	6.41225000	1.54976100	0.70269000
C	5.87439800	0.46832200	-1.11562200
H	4.96049100	-0.81906000	-2.62432600
H	2.17487100	5.09527100	-2.27328500
C	0.13834600	5.00517400	-1.49177600
C	1.03710500	3.62197900	-3.41245400
C	-1.30363800	3.21783100	-2.54478900
H	-0.29611000	2.02170700	-4.06771200
H	-1.83310500	4.38056900	-0.78128800
H	1.00949100	3.01876700	5.76327400
H	-1.88376800	-1.48508000	3.01533000
H	-1.99022100	0.27526400	3.29218400
H	-1.39450900	-0.80553100	4.59771000
H	0.89298600	-1.59924700	4.52686100
H	1.76263700	-1.58697200	2.95399300
H	0.27755900	-2.55567000	3.13325500
H	6.90856400	-0.89458700	1.05479000
H	5.77309900	-0.28808900	2.28723800
H	4.28981900	-2.64076700	-1.03590400
H	6.03458400	-2.30227900	-0.89963300
H	5.74009800	1.38491700	-1.71361600
H	6.89698400	0.09594700	-1.29097600
H	-0.25461000	5.75746600	-2.19540100
H	0.39911200	5.52331400	-0.55349500
H	1.93799300	3.15061100	-3.84065200
H	0.65980600	4.35608700	-4.14323700
H	-2.07396600	2.46130300	-2.34996300
H	-1.71037300	3.95362900	-3.25849000
Sb	-3.88230300	0.58344500	-0.07016700
F	-2.54033800	1.02491200	1.22992300
F	-3.84818700	2.39794400	-0.66175100
F	-2.52209300	0.16448400	-1.36481000
F	-5.19615100	0.13127100	-1.37248900
F	-3.85712300	-1.22640000	0.55214200
F	-5.22399400	1.00818300	1.21455900

Sum of electronic and zero-point Energies= -2469.162001
Sum of electronic and thermal Free Energies= -2469.241690

2-SbF$_6$

C	0.50535200	-1.59054500	-1.83624200
C	-0.42923200	-1.27761700	-2.83422300
H	-1.33775300	-0.73440800	-2.59216700
I	-1.24048600	-3.55423000	0.09025000
Au	0.15565000	-1.13762900	0.16864100
C	1.63624300	-2.36430100	-2.13874100
H	2.34085900	-2.65220600	-1.36212100
C	1.86802900	-2.77061300	-3.46564500
H	2.75757700	-3.36063300	-3.69252500
C	0.96252600	-2.42521800	-4.47955600
H	1.14219800	-2.74142200	-5.50794400
C	-0.18806600	-1.68801000	-4.15743100
H	-0.91683600	-1.43547700	-4.92947300
P	1.50237100	0.79485700	0.39852700
C	1.30227600	1.17570100	2.18421700
C	3.31642800	0.36373400	0.14739500
C	0.88328200	2.27575300	-0.59125900
C	1.87148800	2.34733100	2.73313200
C	0.58421100	0.30692700	3.02617000
C	3.56345800	-1.00737600	0.84246600
C	4.27631100	1.40278500	0.79903400
C	3.62702000	0.26698600	-1.37100800
C	1.90248400	3.44694500	-0.69445100
C	0.52116300	1.78491200	-2.01921200
C	-0.41122900	2.77854600	0.10579500
H	2.42847400	3.02752800	2.09669400
C	1.73661000	2.64322800	4.09280200
C	0.46864400	0.59777800	4.39774700
N	-0.02772100	-0.92935400	2.50265200
H	3.36266100	-0.90408600	1.91957200
H	2.88291400	-1.77179900	0.44549100
C	5.02893900	-1.45369300	0.61969300
C	5.74805300	0.96217200	0.56898500
H	4.09107600	1.45504300	1.87986800
H	4.11424700	2.39967700	0.37058600
C	5.09042200	-0.19655300	-1.57327500
H	3.49180000	1.24951600	-1.84187100
H	2.93943100	-0.43378000	-1.85378000
H	2.16950500	3.82080900	0.30185900
H	2.82145300	3.11575300	-1.19545200
C	1.26611100	4.60486500	-1.51006700
C	-0.08391700	2.94737700	-2.84198600
H	1.40739900	1.37987900	-2.52588100
H	-0.22250200	0.99156000	-1.93561300
C	-1.03588300	3.92919200	-0.72263400
H	-0.17771800	3.14428500	1.11564700
H	-1.12702700	1.95771600	0.19236700
H	2.17880300	3.55487200	4.49409500
C	1.03683100	1.76043900	4.92839700
H	-0.06559100	-0.08650700	5.05345400
C	-1.47397400	-1.00886100	2.89535100
C	0.70107500	-2.11525100	3.06821000
H	5.17212500	-2.43152900	1.10495900
C	5.98338100	-0.40897100	1.24275000
C	5.30172700	-1.57215500	-0.89905800
H	6.40431400	1.72077800	1.02304100
C	6.03987800	0.84963800	-0.94528600
H	5.28126300	-0.27723900	-2.65446200
H	1.99963000	5.42475400	-1.56139800
C	-0.02395300	5.09503100	-0.80859700
C	0.93184400	4.10735600	-2.93503300
C	-1.37552300	3.43022900	-2.14607000
H	-0.31802200	2.56723600	-3.84880200
H	-1.95714000	4.25109500	-0.21515100
H	0.93065300	1.97344700	5.99215600
H	-1.89821800	-1.89908600	2.42788800
H	-1.98866800	-0.11889400	2.53258900
H	-1.56746800	-1.09156000	3.98734400
H	0.63304100	-2.10379700	4.16627200
H	1.74890100	-2.07290700	2.75380800
H	0.22982300	-3.02151100	2.67466500
H	7.02980800	-0.72211300	1.09744100
H	5.79906700	-0.33215200	2.32691300
H	4.62284900	-2.31578400	-1.34598400
H	6.33558200	-1.91455900	-1.06587200
H	5.89323500	1.82737900	-1.43326900
H	7.08918200	0.54974100	-1.09976200
H	-0.45935300	5.93224000	-1.37804600
H	0.21657800	5.46246600	0.20290200
H	1.85155000	3.76664700	-3.43956100
H	0.50705400	4.93349600	-3.52834200
H	-2.09971900	2.60937100	-2.08100400
H	-1.83369600	4.24805800	-2.72611100
Sb	-4.00807100	0.67161800	0.01896600
F	-3.03816500	1.50034400	1.45631800
F	-4.15624400	2.38778000	-0.80175500
F	-2.35558500	0.38925700	-0.92936200
F	-4.91800600	-0.14197900	-1.44210700
F	-3.80441600	-1.03191400	0.86135900
F	-5.63601500	0.94635000	0.96567100

Sum of electronic and zero-point Energies= -2469.192602
Sum of electronic and thermal Free Energies= -2469.268958

1_H-SbF6

Au	0.56805800	-0.76189000	0.44165000
P	-1.48275900	0.14852300	0.38804400
C	-2.13013800	0.41239500	2.08864000
C	-1.32212200	1.84978300	-0.41191100
C	-2.61775100	-1.07132100	-0.51597800
C	-3.44173800	0.89068700	2.30827400
C	-1.30658200	0.13820600	3.20236300
C	-0.19354400	2.60973900	0.35187000
C	-2.61661100	2.70639800	-0.34081800
C	-0.90218200	1.65544700	-1.89255600
C	-3.95173900	-0.44831700	-1.00707600
C	-1.87444800	-1.68530200	-1.74372100
C	-2.90293700	-2.22000000	0.49598300
H	-4.09121800	1.12167000	1.46876300
C	-3.91710400	1.08201700	3.61336800
C	-1.78557700	0.33649600	4.50606900
H	-0.48330300	2.73823700	1.40653600
H	0.74161200	2.03221400	0.32286600
C	0.05115100	3.98778000	-0.31023600
C	-2.36658000	4.07972200	-1.02182200
H	-2.89200500	2.87236200	0.70964600
H	-3.44953900	2.19387000	-0.83783900
C	-0.64060000	3.03448500	-2.54568300
H	-1.70926600	1.14631100	-2.43747000
H	0.00336800	1.03026200	-1.94389300
H	-4.49909300	0.00882900	-0.17301200
H	-3.73806900	0.33203300	-1.75055500
C	-4.83427400	-1.54640300	-1.65681200
C	-2.76181000	-2.76155400	-2.41751400
H	-1.61648500	-0.90533300	-2.47045200
H	-0.93938900	-2.16052900	-1.41081100
C	-3.78025500	-3.30545100	-0.17541900
H	-3.42308500	-1.82504100	1.38042700
H	-1.94334100	-2.65231300	0.82734100
H	-4.93256000	1.44942500	3.76574800
C	-3.09195600	0.80437100	4.71505200
H	-1.13597100	0.12136700	5.35519700
H	0.85266600	4.50094600	0.24318200
C	-1.24532100	4.82842800	-0.26511300
C	0.48419200	3.76868800	-1.77954300
H	-3.30066900	4.66121700	-0.98072100
C	-1.94304300	3.86824900	-2.49477800
H	-0.33852400	2.87461600	-3.59237800
H	-5.78207300	-1.08521800	-1.97530700
C	-5.11581900	-2.66773400	-0.62850900
C	-4.09543500	-2.13168000	-2.88088900
C	-3.04270000	-3.89155900	-1.40128700
H	-2.21584400	-3.16432000	-3.28460400
H	-3.97545000	-4.10049800	0.56063600
H	-3.46421200	0.95412400	5.72920500
H	-1.07026800	5.81171900	-0.73120800
H	-1.54722900	4.99843900	0.78140000
H	1.41033500	3.17129800	-1.81188900
H	0.69030200	4.73959200	-2.25846400
H	-2.74260800	3.34538500	-3.04552800
H	-1.78238500	4.84502200	-2.97928800
H	-5.76151700	-3.43627200	-1.08348100
H	-5.64768600	-2.25281400	0.24380800
H	-3.89964100	-1.33379300	-3.61600300
H	-4.72108200	-2.89688700	-3.36843300
H	-2.09269300	-4.35158100	-1.08264000
H	-3.65953300	-4.67599400	-1.86923100
Sb	4.12471300	-0.43303300	-0.11319500
F	3.36184000	0.91476400	1.01008500
F	5.06205400	-1.12915800	1.38161600
F	2.61560100	-1.62778400	0.45397900
F	4.68805900	-1.88598500	-1.19614800
F	3.00297200	0.17248300	-1.54032200
F	5.54953100	0.69166100	-0.64653800
H	-0.29010500	-0.22742700	3.05460300

Sum of electronic and zero-point Energies= -2092.493687
Sum of electronic and thermal Free Energies= -2092.562462

TSexchange [1_H-SbF_6→1_H-PhI/SbF_6]

C	-1.90177200	1.49218500	1.96920300
C	-2.89325500	2.45403600	2.26831100
H	-3.30440600	3.08770900	1.48759000
C	-3.36042600	2.60808500	3.58226500
H	-4.12548400	3.35583600	3.79472500
C	-2.84873300	1.80689300	4.61516300
H	-3.21513300	1.92827600	5.63531000
C	-1.86032900	0.85241800	4.32940400
H	-1.45154200	0.22806300	5.12493700
C	-1.38906400	0.69654900	3.01750000
C	-0.39729200	2.85463400	-0.24817800
C	0.66228500	3.11313900	0.86686200
H	0.15709800	3.26197500	1.83307700
H	1.32766800	2.24145900	0.95470800
C	1.50061700	4.36679000	0.51743200
H	2.23716300	4.52591200	1.32020000
C	0.56690200	5.59487700	0.40284600
H	1.15818400	6.49569400	0.17034400
H	0.05170100	5.76601100	1.36268500
C	-0.47209700	5.34724700	-0.71514600
H	-1.14943800	6.21208600	-0.79541700
C	0.25510600	5.12177800	-2.06148600
H	-0.48376000	4.95624800	-2.86328100
H	0.84284000	6.01683200	-2.32253000
C	1.18798700	3.89381600	-1.94154500
H	1.69984500	3.71518200	-2.90006000
C	2.23062800	4.13518200	-0.82624200
H	2.89755000	3.26437500	-0.73838700
H	2.84608300	5.01554700	-1.07457300
C	-1.32087400	4.09528700	-0.36670500
H	-1.84402300	4.27011100	0.58313100
H	-2.07053100	3.93626800	-1.15337900
C	0.34501500	2.63975200	-1.59912100
H	-0.38113300	2.46166400	-2.40383000
H	1.00583100	1.76360700	-1.53162400
C	-2.73324500	0.66520900	-0.78869700
C	-3.89290400	1.68266700	-0.95462200
H	-4.27815800	1.98291400	0.02852300
H	-3.54123300	2.57999200	-1.48085700
C	-5.04489400	1.03476200	-1.76871700
H	-5.85175600	1.77666200	-1.87533300
C	-5.57203900	-0.21233700	-1.02043700
H	-6.40432300	-0.66278500	-1.58570600
H	-5.95486500	0.08003200	-0.02863100
C	-4.42498800	-1.23651700	-0.85982100
H	-4.77919000	-2.12299700	-0.31270800
C	-3.89528200	-1.65566900	-2.25002600
H	-3.07729400	-2.38389500	-2.13114200
H	-4.69884400	-2.13804900	-2.83048300
C	-3.37914500	-0.40658500	-3.00130000
H	-2.99292700	-0.69750600	-3.99073400
C	-2.22575400	0.24244900	-2.19730200
H	-1.85946000	1.12330900	-2.74153800
H	-1.39365500	-0.47128700	-2.10032400
C	-3.27381700	-0.59759400	-0.04860500
H	-3.63605500	-0.31637900	0.95139000
H	-2.46488600	-1.32972600	0.07699900
C	-4.52607200	0.61795600	-3.16429200
H	-4.16108400	1.50386600	-3.71024200
H	-5.34747500	0.17480900	-3.75091700
P	-1.26835600	1.25039400	0.25654400
Au	0.30432500	-0.37787500	0.19496300
I	0.51867700	-3.13678200	-0.96369500
C	-1.11412000	-3.54371300	0.41422000
C	-2.27654200	-4.15566800	-0.06573500
C	-0.98306400	-3.11888600	1.74314400
C	-3.35378700	-4.32906300	0.82210300
H	-2.35625000	-4.47580100	-1.10262200
C	-2.07375700	-3.29475900	2.61126200
H	-0.06668800	-2.64510600	2.09052700
C	-3.25804800	-3.89366400	2.15296900
H	-4.26812200	-4.79997300	0.45980000
H	-1.98934500	-2.95585400	3.64439400
H	-4.10173000	-4.02279800	2.83125300

F	2.38070200	-1.00607500	1.07224600
Sb	3.98601100	-0.45755300	0.06197400
F	3.65531000	1.32137100	0.68161900
F	2.90198300	-0.07864300	-1.48208600
F	5.53735900	0.07122800	-0.89278400
F	4.11611400	-2.26369900	-0.53057400
F	4.98479700	-0.84462300	1.63157800
H	-0.61776000	-0.04494400	2.81146500

Sum of electronic and zero-point Energies= -2335.355844
Sum of electronic and thermal Free Energies= -2335.432053

1_H-PhI/SbF6

C	-0.88014600	-3.43322200	0.96056800
C	-0.57580700	-2.71645200	2.12320800
H	0.39342600	-2.24076000	2.25216800
I	0.62924300	-3.46735300	-0.62356000
Au	0.10751800	-0.70818300	-0.67397800
C	-2.12325100	-4.03106100	0.73777200
H	-2.33584300	-4.56947900	-0.18396800
C	-3.10418600	-3.90211200	1.73875500
H	-4.08249400	-4.35762000	1.58328000
C	-2.83287200	-3.18738600	2.91558000
H	-3.60354600	-3.08635300	3.68000300
C	-1.57258200	-2.59889900	3.10669300
H	-1.35513300	-2.03682100	4.01539600
P	-1.08449500	1.21820000	-0.39826900
C	-0.80619300	2.39597300	-1.77615400
C	-2.90367500	0.72197600	-0.49052100
C	-0.56771400	2.02041800	1.24277400
C	-1.32203900	3.71058500	-1.74493800
C	-0.08353600	1.96910700	-2.91063400
C	-3.09537800	-0.08425600	-1.81263400
C	-3.88366200	1.92640900	-0.51215300
C	-3.22366800	-0.20659900	0.71095700
C	-1.61642800	2.98770000	1.85776200
C	-0.27044800	0.89790700	2.28509500
C	0.75544800	2.78862300	0.95857100
H	-1.89224600	4.05559000	-0.88621200
C	-1.10631600	4.58136700	-2.82256000
C	0.12478400	2.84194100	-3.98905800
H	-2.85107500	0.55410000	-2.67609200
H	-2.41525400	-0.95021100	-1.82448200
C	-4.55375100	-0.59146600	-1.91697900
C	-5.34388400	1.40737300	-0.59939500
H	-3.66785000	2.55885700	-1.38510100
H	-3.76497200	2.53807800	0.38989900
C	-4.68034900	-0.71721300	0.60203800
H	-3.10402700	0.34666700	1.65284500
H	-2.52466500	-1.05657900	0.72007000
H	-1.86718500	3.79297200	1.15417900
H	-2.53843300	2.43555600	2.08903200
C	-1.05243900	3.61050800	3.16369700
C	0.27842400	1.51450400	3.59427300
H	-1.17907300	0.31878300	2.49147800
H	0.48564700	0.21292100	1.88749400
C	1.30176900	3.40112400	2.27030100
H	0.57293800	3.58918800	0.22724000
H	1.50091900	2.10587300	0.53599400
H	-1.50491300	5.59575400	-2.78250300
C	-0.38141200	4.15022100	-3.94562400
H	0.68958200	2.49902100	-4.85680800
H	-4.66010100	-1.15645100	-2.85642100
C	-5.52657300	0.60873100	-1.91088500
C	-4.85340700	-1.51401100	-0.71139600
H	-6.02262700	2.27439600	-0.59072300
C	-5.64592400	0.49064600	0.61182800
H	-4.88466600	-1.37038900	1.46400800
H	-1.80690400	4.30279800	3.56954900
C	0.25552700	4.37799100	2.85443400
C	-0.76321600	2.48807500	4.18781400
C	1.59180200	2.27028300	3.28359700
H	0.47636700	0.69497100	4.30358200
H	2.23433700	3.93723400	2.03655800
H	-0.21198300	4.83056800	-4.78114400
H	-6.56558100	0.24953500	-1.99103400
H	-5.32371600	1.25936100	-2.77737600
H	-4.16506500	-2.37446000	-0.71697300
H	-5.88286500	-1.90192700	-0.78206800
H	-5.52620400	1.05555100	1.55146700
H	-6.68910600	0.13808100	0.56073400
H	0.64414600	4.83772700	3.77789600
H	0.05477500	5.18845900	2.13396200
H	-1.69431400	1.94588800	4.42347500
H	-0.38114300	2.92606200	5.12450300
H	2.33223400	1.57941400	2.85481500
H	2.01018300	2.69472300	4.21106700
Sb	3.87354700	0.07785300	-0.17817000
F	2.60730100	1.07929800	-1.22654400
F	4.14668500	1.63544000	0.89175900
F	2.56975400	-0.48877600	1.12371500
F	5.15591900	-0.93382600	0.80191700
F	3.45037400	-1.45429000	-1.25832500
F	5.22795200	0.60923400	-1.40761000
H	0.32390600	0.95917000	-2.94714500

Sum of electronic and zero-point Energies= -2335.363248
Sum of electronic and thermal Free Energies= -2335.440605

$TS1_H$/SbF6

C	-0.42835400	-2.17542800	-2.43375500
H	-1.40639300	-1.70785100	-2.36604900
I	-1.21084800	-3.44499700	0.67249700
Au	-0.04019300	-0.99982400	0.36471000
C	1.53711100	-3.15516200	-1.29550600
H	2.04307500	-3.42636500	-0.37286100
C	2.12411500	-3.39302600	-2.55142600
H	3.10416900	-3.86988800	-2.58850200
C	1.45946900	-3.02469000	-3.73236000
H	1.92139200	-3.22362500	-4.69953100
C	0.19366400	-2.41941100	-3.67250200
H	-0.33430000	-2.13864900	-4.58471400
P	1.32716200	0.90111000	0.45178600
C	1.12188900	1.68836200	2.10033600
C	3.11906400	0.32586000	0.38893900
C	0.85024600	2.11031500	-0.91768900
C	1.59470600	2.99398900	2.35697100
C	0.52483300	0.95999600	3.15190300
C	3.25956200	-0.85950200	1.39281200
C	4.11906900	1.44201200	0.79620300
C	3.45213100	-0.18017400	-1.03925000
C	1.93417800	3.16038400	-1.29326400
C	0.49546600	1.27854500	-2.18948800
C	-0.43472500	2.83989600	-0.43802300
H	2.06981900	3.56995800	1.56800100
C	1.46029500	3.55979400	3.63255000
C	0.39692900	1.52741400	4.42854500
H	2.99529100	-0.52347100	2.40729300
H	2.57218900	-1.67178600	1.11090800
C	4.70795200	-1.40424300	1.36536700
C	5.56619900	0.88522100	0.74347500
H	3.89671700	1.78577300	1.81636700
H	4.02598600	2.30168400	0.12058600
C	4.89351600	-0.74710900	-1.06719100
H	3.38022500	0.64568000	-1.75975300
H	2.73235900	-0.95341500	-1.33428700
H	2.21149600	3.76635800	-0.42081200
H	2.84104500	2.65764100	-1.65579200
C	1.38752000	4.09503900	-2.40484600
C	-0.02845700	2.21262900	-3.30631000
H	1.37474700	0.72331400	-2.54231700
H	-0.29226300	0.55765800	-1.94557000
C	-0.96775100	3.76587100	-1.55684700
H	-0.21653900	3.43884900	0.45712300
H	-1.20017100	2.10495500	-0.17548200
H	1.82556100	4.57156400	3.81209900
C	0.86004100	2.82935600	4.67089600
H	-0.07185100	0.95139700	5.22704800

H	4.78289900	-2.23422700	2.08538600
C	5.69939000	-0.28179500	1.74877100
C	5.01991300	-1.91518600	-0.06124700
H	6.26074500	1.69512100	1.01586000
C	5.88386600	0.37832000	-0.68428600
H	5.10769500	-1.10605600	-2.08615900
H	2.16671500	4.83578000	-2.64419900
C	0.11251500	4.81556200	-1.90533600
C	1.05240400	3.25989700	-3.66206200
C	-1.30718500	2.92464000	-2.80776100
H	-0.26036900	1.59636600	-4.18963100
H	-1.87676500	4.25903200	-1.18430000
H	0.75387700	3.27320900	5.66140600
H	6.72989200	-0.67255500	1.73578700
H	5.48558400	0.07596100	2.76947700
H	4.31672000	-2.71945500	-0.33143600
H	6.04039800	-2.32996700	-0.09588600
H	5.80241500	1.20872400	-1.40503600
H	6.91752400	-0.00233900	-0.72227500
H	-0.25921100	5.49628700	-2.68875700
H	0.34861300	5.42221500	-1.01509100
H	1.96124100	2.75451300	-4.02972400
H	0.68598300	3.92249200	-4.46335000
H	-2.07568500	2.18383900	-2.54557800
H	-1.70923900	3.57625500	-3.60101300
Sb	-3.79057500	0.54686300	0.19837700
F	-2.35623800	0.77094500	1.46790200
F	-3.59004500	2.36831100	-0.33772500
F	-2.53581900	0.04315700	-1.17547400
F	-5.18865200	0.30495600	-1.07240200
F	-3.94887600	-1.28020200	0.73369600
F	-5.02458300	1.06810700	1.55232000
H	0.15246200	-0.05044600	2.98278200

Sum of electronic and zero-point Energies= -2335.338798
Sum of electronic and thermal Free Energies= -2335.413497

2_H-SbF_6

C	0.44270000	-1.78886700	-1.71562100
C	-0.38084000	-1.42242100	-2.78095100
H	-1.23958200	-0.77177500	-2.64559700
I	-1.33961500	-3.45629300	0.62884000
Au	-0.09899600	-1.06624200	0.14646500
C	1.50321700	-2.68717000	-1.83423700
H	2.09513000	-2.99756800	-0.97999000
C	1.79598200	-3.18667500	-3.11820900
H	2.62923900	-3.88151700	-3.22967700
C	1.02154100	-2.80874300	-4.22445700
H	1.25323800	-3.20405600	-5.21379800
C	-0.06470800	-1.93856900	-4.05345700
H	-0.69179200	-1.65403600	-4.89935400
P	1.38509700	0.77110600	0.48054900
C	1.17651600	1.12340100	2.27636000
C	3.16864100	0.20355300	0.30735400
C	0.91069300	2.25927600	-0.56877600
C	1.38303300	2.41684200	2.80679300
C	0.87423800	0.06382300	3.16469900
C	3.30056900	-1.15934600	1.05226600
C	4.14960300	1.21080300	0.97817600
C	3.54126800	0.04145800	-1.18800100
C	2.01401500	3.35121900	-0.66936500
C	0.58901100	1.73609700	-2.00000500
C	-0.39320000	2.86210000	0.02518200
H	1.63494600	3.25143000	2.15911600
C	1.27379200	2.64294700	4.18544300
C	0.75612600	0.29912100	4.54202400
H	3.06824600	-1.01523500	2.11724400
H	2.58468400	-1.88971300	0.65364300
C	4.74512500	-1.69698700	0.90814800
C	5.60152500	0.67479400	0.84075500
H	3.89595200	1.32356800	2.04234800
H	4.07624000	2.19574900	0.50128700
C	4.98433400	-0.50521900	-1.30702800
H	3.48600200	1.01492600	-1.69270200
H	2.83592300	-0.63350500	-1.68257600
H	2.29282800	3.73008900	0.32227200
H	2.91548400	2.92814300	-1.13020800
C	1.49753700	4.52082500	-1.54988400
C	0.10356400	2.90396700	-2.89327000
H	1.47220500	1.26096500	-2.44661400
H	-0.20480700	0.98972100	-1.93032700
C	-0.88407200	4.02833500	-0.86678600
H	-0.22575000	3.23944400	1.04031700
H	-1.16301200	2.09024800	0.07569000
H	1.44061800	3.64725900	4.57571700
C	0.95324100	1.58890600	5.05598500
H	0.51285800	-0.53004600	5.20656400
H	4.80730200	-2.66811200	1.42333700
C	5.72415000	-0.69048300	1.55604100
C	5.08690600	-1.87198900	-0.59052700
H	6.28099800	1.40477700	1.30730400
C	5.95862700	0.50407500	-0.65515500
H	5.22421100	-0.62323600	-2.37500800
H	2.28580300	5.28854700	-1.59424000
C	0.21143400	5.11708200	-0.93031000
C	1.19863200	3.99190200	-2.97216200
C	-1.18617800	3.50605500	-2.28863400
H	-0.10265500	2.50274600	-3.89797400
H	-1.80156200	4.43249400	-0.41602800
H	0.86225300	1.77240300	6.12694700
H	6.75722000	-1.06447000	1.47057100
H	5.49073400	-0.57783200	2.62730000
H	4.39197500	-2.59019500	-1.05384900
H	6.10778500	-2.27313600	-0.69545700
H	5.89114900	1.47538900	-1.17274900
H	6.99506500	0.14179500	-0.75006600
H	-0.13633100	5.96445500	-1.54331200
H	0.42493000	5.49915400	0.08195200
H	2.11732500	3.57301600	-3.41569600
H	0.85780700	4.81932500	-3.61531700
H	-1.97441600	2.74249800	-2.23651700
H	-1.54496200	4.33309000	-2.92291200
Sb	-3.77247300	0.58806900	0.15362500
F	-2.36788800	0.29974200	1.45473800
F	-3.50370400	2.46726900	0.34889200
F	-2.45833300	0.56278800	-1.25576700
F	-5.11708600	0.91032900	-1.15438800
F	-3.98979300	-1.29423000	-0.06868200
F	-5.03480900	0.59032900	1.57649200
H	0.73909500	-0.95495700	2.80008700

Sum of electronic and zero-point Energies= -2335.347585
Sum of electronic and thermal Free Energies= -2335.422729

PhBr

C	-2.21599900	-1.21519600	0.00000000
C	-0.80982200	-1.22823500	0.00000200
C	-0.14042200	-0.00002900	0.00000100
C	-0.80981400	1.22823000	0.00000200
C	-2.21594900	1.21522400	-0.00000100
C	-2.91868100	0.00000700	-0.00000200
H	-2.75423200	-2.16376500	0.00000000
H	-0.26327500	-2.16960800	0.00000200
H	-0.26317800	2.16955200	0.00000300
H	-2.75422000	2.16377200	0.00000200
H	-4.00898800	0.00005200	0.00000000
Br	1.84880100	0.00000000	0.00000000

Sum of electronic and zero-point Energies= -244.805634
Sum of electronic and thermal Free Energies= -244.836729

TSexchange [1-SbF_6→1-PhBr/SbF_6]

C	-2.12820500	0.78362200	1.94941600
C	-3.27007000	1.54426200	2.29440200
H	-3.67703700	2.25781500	1.58356800
C	-3.89972900	1.40047600	3.53590900
H	-4.78004500	2.00024800	3.76866400
C	-3.39364100	0.48168100	4.46753400
H	-3.87546000	0.35693900	5.43830200

C	-2.25379000	-0.26552000	4.15106700
H	-1.84155800	-0.96408200	4.87895900
C	-1.60256700	-0.12788100	2.90812900
C	-0.54552700	-2.33333200	2.86461600
H	0.37386900	-2.81536600	2.50702500
H	-1.38895900	-2.70542200	2.27043900
H	-0.69485700	-2.61083100	3.92730000
C	0.76018500	-0.33804600	3.37325300
H	0.65215800	-0.42477800	4.47350300
H	0.89007500	0.71760500	3.10706900
H	1.65511100	-0.88819000	3.05441600
C	-0.66321200	2.68341900	0.15967000
C	0.51613100	2.70701400	1.17673000
H	0.11822100	2.57381900	2.19435600
H	1.21800100	1.88934300	0.97266600
C	1.27587900	4.05152600	1.07790600
H	2.11126500	4.03020600	1.79427300
C	0.31259500	5.21401000	1.41054500
H	0.84552400	6.17691700	1.34572200
H	-0.06760100	5.10518500	2.44006100
C	-0.86568400	5.19925600	0.41072200
H	-1.57094300	6.01073200	0.64995600
C	-0.32382900	5.38158700	-1.02689000
H	-1.15953000	5.39128800	-1.74692700
H	0.19932000	6.34882200	-1.10476500
C	0.64892000	4.22618700	-1.36347300
H	1.03385400	4.34587400	-2.38838600
C	1.82508000	4.21432600	-0.35922900
H	2.50449100	3.38030800	-0.58692200
H	2.39346000	5.15550900	-0.43859100
C	-1.62495600	3.84817400	0.52409200
H	-1.98092200	3.72343000	1.55682500
H	-2.49750500	3.86154600	-0.13923800
C	-0.11287600	2.88178200	-1.27825900
H	-0.94497900	2.88487900	-1.99759600
H	0.56162100	2.05170000	-1.52903300
C	-2.81433400	0.64491100	-1.00114800
C	-3.72469000	1.86536500	-1.30229400
H	-4.19252000	2.24065800	-0.38299600
H	-3.12494900	2.67467500	-1.74123000
C	-4.83646200	1.45692000	-2.30546300
H	-5.48077600	2.33231400	-2.48197300
C	-5.67653500	0.29685000	-1.71845800
H	-6.47531500	0.02194700	-2.42633800
H	-6.15334400	0.61580000	-0.77688000
C	-4.76191600	-0.92407000	-1.45544500
H	-5.35464100	-1.74958200	-1.03056400
C	-4.10423500	-1.37454600	-2.78029200
H	-3.45530000	-2.24718100	-2.60142500
H	-4.88314900	-1.67607300	-3.49963000
C	-3.27035200	-0.20923000	-3.36141000
H	-2.78154100	-0.52713800	-4.29538300
C	-2.16965700	0.18071400	-2.34518700
H	-1.53972400	0.98091500	-2.75724500
H	-1.52232800	-0.68955600	-2.16709400
C	-3.67400700	-0.51861100	-0.43136900
H	-4.16088900	-0.20106400	0.50097900
H	-3.02187100	-1.37014100	-0.19233400
C	-4.18624400	1.00543200	-3.63232900
H	-3.59619200	1.83170100	-4.06218400
H	-4.96746200	0.73237200	-4.36036300
N	-0.40339000	-0.88520600	2.65621400
P	-1.42525200	0.96443800	0.25360500
Au	0.23287300	-0.55440800	0.00209700
Br	1.33210300	-3.21478500	-1.15296900
C	-0.24765700	-4.03131200	-0.28048700
C	-1.50824000	-3.51005300	-0.58007500
C	-0.05216500	-5.06926400	0.63337600
C	-2.62562200	-4.06226600	0.06827400
H	-1.62119700	-2.68975400	-1.28416600
C	-1.18468400	-5.60763600	1.27068300
H	0.94445900	-5.44631300	0.85720500
C	-2.46655500	-5.10756600	0.99200000
H	-3.61484400	-3.66060700	-0.15215100
H	-1.05302000	-6.41686900	1.98947300
H	-3.33742500	-5.53054600	1.49299700
F	2.65042800	-0.87047200	0.69286400
Sb	3.96407000	0.11577300	-0.36130900
F	3.68002500	1.59160500	0.82126900
F	2.60071700	0.81459900	-1.52136500
F	5.26812200	1.08110400	-1.35033100
F	4.08609100	-1.40088300	-1.51639500
F	5.29469500	-0.60117400	0.79373000

Sum of electronic and zero-point Energies= -2471.129039
Sum of electronic and thermal Free Energies= -2471.208372

1-PhBr/SbF_6

C	0.29494300	-3.61908800	-0.49099100
C	0.04582900	-3.05007100	-1.74076800
H	-0.84282200	-2.45035100	-1.91878500
Br	-1.01693200	-3.22598800	0.96856100
Au	-0.20669200	-0.56615900	0.70464400
C	1.42823900	-4.37240400	-0.18790400
H	1.59166200	-4.78578100	0.80545500
C	2.36879100	-4.56664600	-1.21677000
H	3.26777500	-5.14630100	-1.00730100
C	2.16144700	-4.00828000	-2.48735300
H	2.90301800	-4.15554100	-3.27249300
C	1.00442400	-3.25621200	-2.74759700
H	0.83956300	-2.81518700	-3.73091200
P	1.31992400	1.03866000	0.21032700
C	1.24328700	2.47548700	1.35920400
C	3.02182700	0.22541400	0.36933100
C	0.93905100	1.69079700	-1.55052500
C	2.06871100	3.58455900	1.05920600
C	0.28583100	2.59714400	2.41439400
C	3.11580300	-0.40369000	1.79119300
C	4.22029700	1.20005500	0.20363500
C	3.10665400	-0.91473100	-0.68365300
C	2.15954200	2.28127000	-2.30799300
C	0.35691700	0.51687000	-2.39831900
C	-0.16349000	2.77788100	-1.41009900
H	2.80142900	3.51119100	0.26021400
C	1.94944900	4.80082300	1.74172300
C	0.16753500	3.84079500	3.07452100
N	-0.53663500	1.50147900	2.80785900
H	3.05465900	0.39355400	2.54779900
H	2.27654500	-1.09862900	1.94906800
C	4.44991300	-1.17400700	1.94907800
C	5.55432900	0.41712300	0.33791900
H	4.17647100	1.96786900	0.98863300
H	4.18651100	1.70126600	-0.77038600
C	4.44067300	-1.68147600	-0.52299300
H	3.04997000	-0.49811300	-1.69821200
H	2.26015200	-1.60398900	-0.55075400
H	2.60282300	3.11240900	-1.74395800
H	2.92853100	1.50810300	-2.43985200
C	1.71536600	2.79700400	-3.70377000
C	-0.06651600	1.02063300	-3.79833200
H	1.09412700	-0.29098500	-2.49030600
H	-0.53361600	0.10885000	-1.90600000
C	-0.59766800	3.27782800	-2.81067900
H	0.21577900	3.62626900	-0.82402500
H	-1.03064800	2.35449200	-0.88865200
H	2.59781200	5.63672500	1.47828200
C	0.97717400	4.93266700	2.74307600
H	-0.56676100	3.94204600	3.87208900
C	-1.90030400	1.82863900	3.23755100
C	0.11674800	0.59364600	3.76464500
H	4.48300200	-1.60712900	2.96113000
C	5.63813200	-0.20624200	1.74966300
C	4.51204200	-2.30015600	0.89162800
H	6.38549400	1.12409500	0.19023700
C	5.61873200	-0.70192600	-0.72906600
H	4.47456800	-2.47890900	-1.28056700
H	2.59647900	3.22424800	-4.20832100
C	0.62558900	3.88198800	-3.53862900
C	1.15290100	1.61868900	-4.53244600
C	-1.16528200	2.09605700	-3.63179400
H	-0.46553300	0.16334200	-4.36370300

H	-1.37364000	4.04753300	-2.67733200
H	0.85815900	5.87606600	3.27801600
H	-2.48120600	0.89708700	3.24599200
H	-2.35740900	2.51922100	2.52007800
H	-1.94041700	2.27401600	4.25248900
H	0.23736000	1.06311200	4.76318800
H	1.09962700	0.29780500	3.38401800
H	-0.50277300	-0.30979200	3.86683000
H	6.58886900	-0.75184900	1.86567000
H	5.60723400	0.58847500	2.51331100
H	3.66951100	-2.99555300	1.03402500
H	5.44830900	-2.87044300	1.00687800
H	5.56565600	-0.26186600	-1.73880200
H	6.57694600	-1.24023900	-0.64490200
H	0.32539200	4.26256100	-4.52862800
H	1.02520500	4.72994600	-2.95782000
H	1.92985600	0.84665500	-4.66275000
H	0.85682100	1.97196200	-5.53386600
H	-2.03748600	1.66425000	-3.11456000
H	-1.49678100	2.45176500	-4.62122600
Sb	-3.92543300	-0.07529900	-0.12196400
F	-2.55660800	-0.92174700	-1.18852500
F	-4.55092700	0.74509500	-1.72627700
F	-4.99671100	-1.62668800	-0.39494900
F	-5.30294500	0.75181200	0.89999000
F	-3.23705900	-0.87118400	1.49568400
F	-2.79482900	1.44262500	0.18650800

Sum of electronic and zero-point Energies= -2471.133201
Sum of electronic and thermal Free Energies= -2471.212326

TS1[1-PhBr/SbF$_6$→ 2$_{Br}$/SbF$_6$]

C	0.23138400	-2.81151100	-1.14904100
C	-0.42527800	-2.47657600	-2.33550900
H	-1.42072600	-2.04259200	-2.32532200
Br	-1.10915800	-3.36806500	0.60103900
Au	-0.01627200	-0.97780700	0.51976300
C	1.49874000	-3.39783200	-1.09043000
H	1.95650900	-3.66656300	-0.14158400
C	2.17704500	-3.58323200	-2.30798500
H	3.17878900	-4.01357900	-2.28807100
C	1.57936500	-3.21548100	-3.52433700
H	2.11265300	-3.37041700	-4.46224000
C	0.28666000	-2.66380400	-3.53325600
H	-0.18888400	-2.37767800	-4.47246400
P	1.37177700	0.84732500	0.31655500
C	1.21272600	1.79041500	1.89228800
C	3.17003600	0.29435500	0.16626600
C	0.79795800	1.94020400	-1.12273700
C	1.82995000	3.05765300	2.00493300
C	0.43484700	1.31364100	2.98431700
C	3.41281400	-0.81266500	1.23252000
C	4.19986300	1.43185200	0.41256600
C	3.38556500	-0.30320400	-1.24899600
C	1.81495400	3.01809700	-1.58037000
C	0.45405700	1.02014300	-2.33589400
C	-0.51361000	2.62468400	-0.65492400
H	2.44653300	3.42961100	1.19220400
C	1.66947600	3.85263100	3.14538300
C	0.28095400	2.12542100	4.12528300
N	-0.16981100	-0.00235400	2.96971900
H	3.27353000	-0.38077900	2.23434200
H	2.68740000	-1.62972300	1.09876000
C	4.85013500	-1.37318800	1.10668500
C	5.63933000	0.86867500	0.26365600
H	4.07481800	1.83030900	1.42909300
H	4.04745400	2.25392800	-0.29874000
C	4.81771000	-0.87838500	-1.36756400
H	3.26584100	0.48424700	-2.00403300
H	2.63888400	-1.08486100	-1.43870800
H	2.07122900	3.68836800	-0.74973100
H	2.73750000	2.53824700	-1.93452700
C	1.19911500	3.86135600	-2.72962700
C	-0.14341600	1.86620600	-3.48867400
H	1.34664400	0.49531900	-2.69404900
H	-0.28020400	0.26797200	-2.03133600
C	-1.11746200	3.46673200	-1.80577300
H	-0.31367300	3.27377900	0.20996000
H	-1.23145300	1.85883000	-0.35072600
H	2.15406800	4.82781300	3.19740400
C	0.88172500	3.38688600	4.20760800
H	-0.31088500	1.75416200	4.96080100
C	-1.56709600	-0.04410200	3.44679200
C	0.64381900	-0.98345000	3.72090200
H	4.98900800	-2.15326800	1.87174900
C	5.86998700	-0.23152600	1.32491000
C	5.03564200	-1.97866200	-0.30385300
H	6.35199200	1.69372200	0.41918500
C	5.83261300	0.26900000	-1.15005800
H	4.93923100	-1.30326500	-2.37627400
H	1.93465400	4.62446800	-3.02978500
C	-0.09808000	4.54692600	-2.23630900
C	0.87794000	2.94065400	-3.92882900
C	-1.44349600	2.54680500	-3.00415000
H	-0.36338800	1.18970500	-4.32998700
H	-2.04190300	3.93712900	-1.43688600
H	0.74362800	3.99482900	5.10261000
H	-1.98104000	-1.02297800	3.17763400
H	-2.14896600	0.73290400	2.94666300
H	-1.63290000	0.08140000	4.54349600
H	0.67127300	-0.73637200	4.79888000
H	1.66247100	-1.00247000	3.32295600
H	0.19080400	-1.97661800	3.59007900
H	6.89632900	-0.62442600	1.24117900
H	5.74944100	0.19136400	2.33588000
H	4.31293900	-2.79538100	-0.45710100
H	6.04982800	-2.39900500	-0.40127500
H	5.68317500	1.04952300	-1.91459200
H	6.86147200	-0.11279400	-1.25345700
H	-0.52139800	5.16570300	-3.04454500
H	0.12940600	5.21029300	-1.38517800
H	1.80205900	2.45822300	-4.28957600
H	0.46239100	3.53735500	-4.75746100
H	-2.17746400	1.79059500	-2.69544900
H	-1.88386800	3.14159300	-3.82176800
Sb	-3.93988300	0.19670000	-0.09105800
F	-2.41213700	-0.20371100	-1.19249100
F	-4.36411400	1.58636800	-1.32840600
F	-4.91011900	-1.09723800	-1.10033000
F	-5.48618000	0.59093000	0.94966700
F	-3.42766800	-1.15413300	1.16207300
F	-2.94743900	1.47589700	0.94362100

Sum of electronic and zero-point Energies= -2471.115831
Sum of electronic and thermal Free Energies= -2471.194200

2Br/SbF6

C	0.53343600	-1.70399100	-1.83700200
C	-0.43121400	-1.45530900	-2.82420900
H	-1.36170400	-0.95378400	-2.57613700
Br	-1.02395100	-3.57019300	0.13896800
Au	0.17030000	-1.26289500	0.16325700
C	1.69773600	-2.42366300	-2.14489400
H	2.42868400	-2.66186600	-1.37612300
C	1.92787600	-2.84357800	-3.46795200
H	2.84231100	-3.39259800	-3.69907800
C	0.98814600	-2.56598900	-4.47148300
H	1.16611600	-2.89332000	-5.49672900
C	-0.19206600	-1.88014900	-4.14330200
H	-0.94576800	-1.68048800	-4.90684400
P	1.41084100	0.72523700	0.39685400
C	1.17477400	1.09154900	2.18225100
C	3.24689000	0.38838100	0.15345100
C	0.72491700	2.17179400	-0.59862300
C	1.67447400	2.29053000	2.73931100
C	0.49497900	0.18391500	3.01623000
C	3.56018300	-0.97336800	0.83877400
C	4.14369500	1.47130500	0.82409500
C	3.57687900	0.32048500	-1.36376400
C	1.67979400	3.39647600	-0.68689000

C	0.41124400	1.66311700	-2.03231400
C	-0.60129200	2.60702000	0.08216700
H	2.20275500	3.00322400	2.11397400
C	1.50801300	2.57507400	4.09823400
C	0.34838200	0.46397900	4.38613200
N	-0.04111500	-1.08401800	2.48505600
H	3.34658100	-0.89081400	1.91503500
H	2.92414700	-1.76791200	0.42815300
C	5.04907800	-1.33947300	0.62601100
C	5.63894200	1.11161700	0.60582400
H	3.94387000	1.50474800	1.90309700
H	3.93540300	2.46194500	0.40150100
C	5.06577300	-0.05965600	-1.55406800
H	3.39500000	1.29876400	-1.82709600
H	2.93507000	-0.41347700	-1.86180500
H	1.91270500	3.78122000	0.31369400
H	2.62172200	3.11937800	-1.17747300
C	0.99171200	4.52026100	-1.50902200
C	-0.24378600	2.79206100	-2.86287900
H	1.32552000	1.30745300	-2.52583500
H	-0.28980500	0.83095700	-1.96150100
C	-1.27924200	3.71858000	-0.75723700
H	-0.39593500	2.98778900	1.09278500
H	-1.27346700	1.75082200	0.16493700
H	1.89645600	3.50818700	4.50565600
C	0.84728400	1.65462300	4.92472400
H	-0.15333300	-0.25169100	5.03347600
C	-1.48094900	-1.25931000	2.87323200
C	0.75645600	-2.22694600	3.04574400
H	5.23870800	-2.31288700	1.10409100
C	5.94072600	-0.25208300	1.26848200
C	5.34366300	-1.42861400	-0.89022500
H	6.24917700	1.89953900	1.07378400
C	5.95008100	1.03070200	-0.90665500
H	5.27164500	-0.11931300	-2.63387400
H	1.68052000	5.37860400	-1.54788400
C	-0.33371500	4.93937200	-0.82878200
C	0.70651600	4.00808900	-2.93934700
C	-1.57048400	3.19950600	-2.18478300
H	-0.44124200	2.40175100	-3.87362600
H	-2.22404700	3.98685600	-0.26237500
H	0.71851100	1.85967300	5.98752000
H	-1.85364200	-2.15715400	2.37605300
H	-2.04742300	-0.39191800	2.53543900
H	-1.56730900	-1.37893200	3.96260700
H	0.67877200	-2.22784300	4.14299200
H	1.80145600	-2.11771500	2.73934700
H	0.34520200	-3.15756700	2.64017300
H	7.00353200	-0.50874700	1.13098400
H	5.74229100	-0.19615200	2.35151100
H	4.71038500	-2.20279000	-1.35210000
H	6.39601900	-1.71407600	-1.04794000
H	5.75373300	2.00371600	-1.38626900
H	7.01531400	0.78998900	-1.05453000
H	-0.80494000	5.75135100	-1.40607200
H	-0.13109300	5.31985600	0.18631300
H	1.65137300	3.72206100	-3.43108700
H	0.24405800	4.81054200	-3.53690700
H	-2.24563200	2.33700800	-2.12667300
H	-2.06867100	3.98862900	-2.77157200
Sb	-4.06014600	0.31799400	0.02626000
F	-2.39392000	0.11156600	-0.91793500
F	-4.27228700	2.02551600	-0.80100200
F	-4.92756000	-0.53407800	-1.43914900
F	-5.70654000	0.53471200	0.95764500
F	-3.79651400	-1.36743200	0.88114600
F	-3.13531700	1.19487600	1.46600100

Sum of electronic and zero-point Energies= -2471.151385
Sum of electronic and thermal Free Energies= -2471.227703

1*

C	-0.14002200	1.12216900	1.55348900
C	0.04012500	0.61457100	2.86290100
H	0.24952400	-0.44060400	3.01092600
C	-0.05445300	1.44065800	3.98733900
H	0.08892700	1.01802900	4.98130200
C	-0.33531100	2.80429700	3.82424500
H	-0.41554900	3.45970700	4.69139700
C	-0.51761500	3.32138000	2.53782400
H	-0.74227300	4.37964200	2.40702700
C	-0.42368700	2.50768400	1.39264500
C	0.32199500	4.20298300	-0.18953500
H	0.16837400	4.53523400	-1.22569900
H	1.34550400	3.82042000	-0.08395600
H	0.18968200	5.07271200	0.48023700
C	-2.03375800	3.53065300	-0.10344900
H	-2.33555200	4.33050700	0.59874300
H	-2.69146300	2.66349400	0.03677800
H	-2.14657400	3.90168600	-1.13179700
C	-1.43846100	-1.23039500	0.21184500
C	-2.72685900	-0.37715000	-0.00064200
H	-2.81711100	0.36256000	0.81057100
H	-2.66553200	0.16875800	-0.95612300
C	-3.96792700	-1.30379600	-0.03040200
H	-4.86155300	-0.68101900	-0.18594900
C	-4.08214700	-2.06700000	1.30913200
H	-4.96767700	-2.72039000	1.29032200
H	-4.20856700	-1.35654100	2.14200300
C	-2.80828800	-2.91511100	1.52296900
H	-2.86820200	-3.44732600	2.48419600
C	-2.65295300	-3.92955700	0.36673800
H	-1.75305800	-4.54705700	0.52156900
H	-3.52094700	-4.60599600	0.34885200
C	-2.54981800	-3.17060900	-0.97755000
H	-2.43575600	-3.88660900	-1.80489300
C	-3.81728000	-2.31067100	-1.19520900
H	-3.74720500	-1.77157700	-2.15410700
H	-4.70421600	-2.96044400	-1.24217400
C	-1.57245500	-1.97566400	1.57369200
H	-1.70981800	-1.24515300	2.38279300
H	-0.66976600	-2.56237800	1.78584600
C	-1.29924100	-2.25713100	-0.94385000
H	-0.40404900	-2.87654200	-0.78661600
H	-1.18295100	-1.72336400	-1.90168500
C	1.75931000	-0.81819900	0.17904400
C	1.90445000	-1.97768300	1.20690300
H	1.64461900	-1.64143400	2.21914200
H	1.23265200	-2.80366600	0.93176200
C	3.37127400	-2.48599900	1.20522500
H	3.45292300	-3.28821900	1.95393600
C	4.32560100	-1.32526900	1.57102500
H	5.36263500	-1.69283300	1.59713700
H	4.08434300	-0.93800700	2.57440300
C	4.19661600	-0.19508900	0.52158400
H	4.87067300	0.63505900	0.78025000
C	4.54390300	-0.74231500	-0.88200300
H	4.46041200	0.06108200	-1.63203000
H	5.58337500	-1.10395000	-0.89562700
C	3.57984000	-1.89787200	-1.23540300
H	3.80429400	-2.27975200	-2.24239100
C	2.12422800	-1.36611100	-1.23604600
H	1.43078700	-2.16510800	-1.52929800
H	2.03845100	-0.55989500	-1.98138300
C	2.74148500	0.33755300	0.52963400
H	2.50435100	0.75069800	1.52037900
H	2.62865900	1.14699500	-0.21251900
C	3.71952000	-3.03368300	-0.19645700
H	3.04838700	-3.86907900	-0.45400100
H	4.75025200	-3.41965900	-0.20133400
N	-0.63089100	3.10913300	0.09196200
P	0.02712000	-0.05502100	0.15192600
Au	-0.14341800	1.19399400	-1.74740200

Sum of electronic and zero-point Energies= -1621.709198

Sum of electronic and thermal Free Energies= -1621.765886

PhI in gas phase

C	-2.68280800	-1.21347100	0.00000000
C	-1.27780200	-1.22447700	-0.00000200
C	-0.59737700	-0.00001800	-0.00000100
C	-1.27779000	1.22446700	0.00000000
C	-2.68277600	1.21349000	-0.00000300
C	-3.38661400	0.00000700	0.00000200
H	-3.22082900	-2.16238700	0.00000800
H	-0.73216500	-2.16639100	-0.00000800
H	-0.73210900	2.16635600	-0.00000200
H	-3.22081600	2.16239400	0.00000500
H	-4.47686700	0.00003800	0.00000200
I	1.58139200	0.00000000	0.00000000

Sum of electronic and zero-point Energies= -242.831116
Sum of electronic and thermal Free Energies= -242.863020

I-adduct from 1*

C	-3.60743000	0.28852700	0.86632700
C	-2.97008500	0.73877300	2.02523600
H	-2.40940800	0.05990800	2.66351400
I	-3.36864400	-1.83665000	0.33193600
Au	-0.72394300	-1.09780800	-0.11594500
C	-4.31037800	1.12257100	-0.00354300
H	-4.78341400	0.73967300	-0.90569000
C	-4.38013800	2.49048000	0.32202600
H	-4.92593600	3.16351200	-0.33907000
C	-3.75612200	2.98143100	1.47811300
H	-3.81639700	4.04229600	1.71881700
C	-3.05628800	2.10881600	2.32581800
H	-2.56906200	2.48490200	3.22558800
P	1.22390600	0.09759700	-0.24726800
C	2.33523300	-0.67727100	-1.48774900
C	0.79020600	1.81560600	-0.88978500
C	2.07548100	0.05731100	1.44567000
C	3.64308400	-0.16507000	-1.65539200
C	1.93339300	-1.79232500	-2.27183700
C	-0.04337500	1.61216800	-2.19337400
C	2.01445100	2.70726900	-1.24015000
C	-0.09579700	2.52188100	0.17228200
C	3.11463500	1.18939400	1.67041900
C	0.99419600	0.15227700	2.56919500
C	2.76836700	-1.33227600	1.56137100
H	3.96468200	0.69429600	-1.07336700
C	4.54041100	-0.73772600	-2.56287000
C	2.84769400	-2.35401400	-3.18382500
N	0.60612900	-2.35183900	-2.16538900
H	0.57402300	1.09803100	-2.94706600
H	-0.92409800	0.98278100	-1.98764700
C	-0.52397500	2.98017000	-2.73497800
C	1.52208900	4.08392900	-1.76311400
H	2.61432700	2.22006700	-2.02197100
H	2.65380900	2.85618600	-0.36091600
C	-0.58192100	3.88457700	-0.37664200
H	0.48666800	2.69087400	1.09069400
H	-0.95432600	1.88180400	0.42610800
H	3.89153300	1.16290200	0.89455500
H	2.61379500	2.16697200	1.61947400
C	3.78032700	1.02485300	3.06181500
C	1.65627400	0.01203500	3.96219300
H	0.45291400	1.10561500	2.49977700
H	0.25938500	-0.65802700	2.44304900
C	3.42634800	-1.47745700	2.95655800
H	3.53365800	-1.44010600	0.77982600
H	2.01583100	-2.12562500	1.41129800
H	5.54278000	-0.32339400	-2.66831300
C	4.14083100	-1.84113100	-3.33094300
H	2.52881500	-3.20467000	-3.78613400
C	0.60118700	-3.78418400	-1.82294900
C	-0.22103200	-2.05894100	-3.34920600
H	-1.11044300	2.80631700	-3.65022900
C	0.69562000	3.87426600	-3.05227000
C	-1.40852000	3.66116500	-1.66399300
H	2.40404900	4.70516100	-1.98004600
C	0.64646000	4.77204100	-0.68826500
H	-1.20928100	4.36451500	0.39003700
H	4.52433200	1.82607000	3.18635100
C	4.47528500	-0.35507500	3.14259600
C	2.69941800	1.13390500	4.16122700
C	2.34651200	-1.36848500	4.05748700
H	0.86985900	0.09096900	4.72880200
H	3.91785000	-2.46053900	3.00946100
H	4.82941100	-2.29533800	-4.04365500
H	-0.43890000	-4.08714100	-1.63578300
H	1.18495300	-3.93526900	-0.90562000
H	1.01444700	-4.42676700	-2.62392200
H	0.16134500	-2.54524200	-4.26762600
H	-0.25261400	-0.97313400	-3.50375100
H	-1.24268800	-2.41664000	-3.15580900
H	0.35644700	4.84590900	-3.44354000
H	1.31951400	3.40067900	-3.82756200
H	-2.28318800	3.03019800	-1.43985600
H	-1.77727000	4.62693800	-2.04380100
H	1.23416700	4.93655600	0.22990700
H	0.31590000	5.75664600	-1.05380000
H	4.97041800	-0.46559600	4.11960100
H	5.25194900	-0.43270200	2.36437300
H	2.20993200	2.12063300	4.11214000
H	3.16430400	1.04155900	5.15487100
H	1.60095400	-2.17162000	3.93646900
H	2.80847700	-1.48892400	5.04952500

Sum of electronic and zero-point Energies= -1864.590642
Sum of electronic and thermal Free Energies= -1864.660262

TS_1*

C	-2.70273800	0.95397000	0.96449000
C	-2.47620200	0.87750900	2.34347500
H	-2.40726900	-0.08182500	2.85030400
I	-3.80734800	-0.96542600	-0.06818700
Au	-1.11513300	-0.51040000	-0.27038500
C	-2.85237200	2.16254200	0.27317400
H	-3.06832400	2.17885900	-0.79235700
C	-2.68800100	3.35320300	1.00254000
H	-2.78646500	4.30610100	0.48244900
C	-2.40899300	3.31738800	2.37699200
H	-2.29142000	4.24689100	2.93294100
C	-2.30686300	2.08405300	3.04199700
H	-2.10657900	2.04779800	4.11334700
P	1.15833000	-0.10518800	-0.26343200
C	1.90843800	-1.12300600	-1.59657300
C	1.45943600	1.70290700	-0.67986200
C	1.85559100	-0.72322200	1.38272500
C	3.30746700	-1.09499400	-1.80143400
C	1.12114000	-1.97854900	-2.40926600
C	0.63999900	2.00530500	-1.97328900
C	2.94206200	2.08241900	-0.94757900
C	0.90430000	2.55888000	0.49201500
C	3.30391500	-0.25755800	1.68879900
C	0.92226400	-0.26745100	2.54489200
C	1.80595800	-2.27860900	1.30776500
H	3.92986100	-0.45382800	-1.18358100
C	3.91455900	-1.87844500	-2.78913200
C	1.74275700	-2.75787100	-3.40204500
N	-0.31631800	-2.07456600	-2.24052400
H	1.01936300	1.38762100	-2.80303200
H	-0.42166300	1.75508600	-1.81919900

C	0.75012200	3.50951000	-2.32663200
C	3.04087200	3.59628700	-1.27717700
H	3.32137000	1.51073700	-1.80567600
H	3.56475400	1.84839800	-0.07359800
C	0.99382700	4.06132700	0.12884500
H	1.50510900	2.37696300	1.39500000
H	-0.13654700	2.27761100	0.70675300
H	3.98787500	-0.56421600	0.88487300
H	3.33245500	0.83914500	1.76902200
C	3.78033200	-0.88283700	3.02717900
C	1.40859600	-0.87137600	3.88512900
H	0.89362600	0.82701900	2.61480900
H	-0.10405300	-0.60932000	2.34833900
C	2.28446800	-2.88677200	2.64911500
H	2.44778600	-2.63787500	0.49066700
H	0.77084400	-2.59796700	1.09290900
H	4.99492600	-1.83654800	-2.92525500
C	3.12768000	-2.71271300	-3.59642400
H	1.12589600	-3.40780900	-4.02241500
C	-0.75898200	-3.42802200	-1.85027500
C	-1.05987000	-1.56597300	-3.41203300
H	0.16555200	3.69726700	-3.24022900
C	2.23050300	3.88633500	-2.56125100
C	0.17792000	4.34150500	-1.15493000
H	4.10021400	3.84599300	-1.43894000
C	2.47704000	4.43275200	-0.10418600
H	0.58466600	4.64604300	0.96684000
H	4.81204400	-0.55042200	3.21649000
C	3.73646000	-2.42581200	2.92405900
C	2.85599900	-0.40781300	4.17124200
C	1.36105100	-2.41364400	3.79495700
H	0.73856900	-0.52160200	4.68580800
H	2.24965000	-3.98360900	2.56703000
H	3.58874100	-3.32648800	-4.37025600
H	-1.83217300	-3.38237300	-1.61676600
H	-0.21047900	-3.73564200	-0.95093900
H	-0.59621300	-4.17101300	-2.65184200
H	-0.91163600	-2.19718900	-4.30715200
H	-0.72566800	-0.54333600	-3.62896700
H	-2.12880000	-1.54694500	-3.15717500
H	2.30694400	4.95375400	-2.81995000
H	2.63956000	3.30701900	-3.40482300
H	-0.87862200	4.07857600	-0.99116600
H	0.22445700	5.41394900	-1.40012200
H	3.06048400	4.24307200	0.81176300
H	2.56510400	5.50509400	-0.33771500
H	4.09838700	-2.87171500	3.86322300
H	4.40055500	-2.76949000	2.11426600
H	2.89227300	0.69079600	4.25425200
H	3.20084500	-0.82658500	5.12914900
H	0.32710900	-2.74907600	3.60880000
H	1.68874900	-2.85732500	4.74774900

Sum of electronic and zero-point Energies= -1864.580894
Sum of electronic and thermal Free Energies= -1864.648666

2*

C	-1.49179600	1.57365200	1.03809700
C	-1.53835100	1.50443300	2.43529900
H	-1.52463000	0.55071000	2.95488300
I	-4.00204700	-0.24067000	0.06670900
Au	-1.29651700	-0.11739300	-0.17399800
C	-1.65591300	2.79861000	0.37379600
H	-1.72145000	2.84940300	-0.71092100
C	-1.76107400	3.98083500	1.12673400
H	-1.87968900	4.93119100	0.60537400
C	-1.73564000	3.93385300	2.52742000
H	-1.81989300	4.85218300	3.10812400
C	-1.64414200	2.69480900	3.17693600
H	-1.67345000	2.63787500	4.26553200
P	1.05960100	-0.27664600	-0.31951300
C	1.29809600	-1.45678000	-1.70521000
C	1.88603400	1.35325200	-0.75277900
C	1.71034900	-1.16192800	1.21314200
C	2.59521400	-1.83518500	-2.12313900
C	0.18543300	-2.08961100	-2.29331600
C	1.02156300	2.00234700	-1.87603200
C	3.33643600	1.21871800	-1.30493200
C	1.89862600	2.25476600	0.51573700
C	3.26078400	-1.14501700	1.30040200
C	1.09946400	-0.51402100	2.48376900
C	1.20147900	-2.63368200	1.15032900
H	3.46634200	-1.38816000	-1.65553500
C	2.77980700	-2.78958800	-3.12832700
C	0.37622400	-3.04292800	-3.30868200
N	-1.19124100	-1.79077700	-1.84600600
H	1.04480800	1.35907700	-2.77104100
H	-0.02093600	2.09209600	-1.55069100
C	1.57578500	3.40966500	-2.21704500
C	3.89954000	2.62908300	-1.63451700
H	3.32347500	0.63010300	-2.23179300
H	3.98947700	0.71642500	-0.57808600
C	2.44161300	3.65780700	0.14720400
H	2.55365300	1.80480300	1.27610400
H	0.89127700	2.34140800	0.93908300
H	3.70029500	-1.61494000	0.40942300
H	3.62244500	-0.10855200	1.36029100
C	3.71222300	-1.92297100	2.56485500
C	1.58220600	-1.26614600	3.74827400
H	1.34806700	0.55204400	2.55029400
H	0.00933300	-0.60048600	2.41398700
C	1.66655400	-3.40191800	2.41431600
H	1.58671300	-3.13731500	0.25230300
H	0.09899000	-2.63160100	1.10001300
H	3.78837900	-3.06365100	-3.43585500
C	1.66428600	-3.38947100	-3.72968400
H	-0.48538200	-3.52458900	-3.76720200
C	-1.84433500	-3.04112700	-1.33030700
C	-1.98465600	-1.23224000	-2.99558900
H	0.93728800	3.85334000	-2.99590900
C	3.02698200	3.27967100	-2.73233600
C	1.54867600	4.29161500	-0.94560000
H	4.92989200	2.50431900	-2.00005500
C	3.89124800	3.51606500	-0.36931100
H	2.42221900	4.28247100	1.05255100
H	4.81156200	-1.90500600	2.60548700
C	3.21213800	-3.38464300	2.47790200
C	3.12613100	-1.23638700	3.82025200
C	1.08748000	-2.72919200	3.67971000
H	1.15560800	-0.76615900	4.63086100
H	1.30665500	-4.43908000	2.34012600
H	1.79297500	-4.13219100	-4.51641200
H	-2.84321000	-2.77912000	-0.96702300
H	-1.23860900	-3.43351000	-0.50618400
H	-1.92206600	-3.79382600	-2.12811100
H	-2.02469900	-1.95616700	-3.82240400
H	-1.50159200	-0.30728900	-3.33241300
H	-2.99722400	-1.01428100	-2.64142300
H	3.42500700	4.27418000	-2.98543600
H	3.05111000	2.66701100	-3.64820600
H	0.51847100	4.38935400	-0.57228700
H	1.91788000	5.29978400	-1.18862000
H	4.52759100	3.06939400	0.41228300
H	4.30575300	4.50711400	-0.60991000
H	3.55414900	-3.94839500	3.35905800
H	3.63310800	-3.87441800	1.58460800
H	3.48320800	-0.19549500	3.88205600
H	3.46763200	-1.76002900	4.72608700
H	-0.01431700	-2.75345300	3.65168700
H	1.41097300	-3.27994100	4.57606800

Sum of electronic and zero-point Energies= -1864.605236

Sum of electronic and thermal Free Energies= -1864.671345

π-adduct from 1*

C	3.47404000	-1.36432300	-0.66013600
C	3.83331600	-2.38176200	0.22687400
H	4.60475300	-2.21430300	0.97631200
I	4.35845100	0.58579900	-0.50373900
Au	0.50530100	-0.98637500	-0.41967100
C	2.47716700	-1.57813200	-1.65262000
H	2.34787000	-0.85907500	-2.46269900
C	1.83078600	-2.84711100	-1.70778200
H	1.11001500	-3.03629800	-2.50279900
C	2.18820900	-3.86986100	-0.80468300
H	1.69516800	-4.83909300	-0.86036900
C	3.19438800	-3.63564100	0.13882800
H	3.49522700	-4.42863300	0.82366800
P	-1.25678200	0.31932800	0.26618900
C	-1.48088000	0.18412600	2.08552100
C	-0.78039400	2.11225300	-0.08051400
C	-2.82015100	-0.36287700	-0.56242900
C	-2.56536400	0.85369300	2.70024900
C	-0.60389200	-0.57672300	2.90149600
C	0.57193300	2.34350400	0.66314200
C	-1.80368700	3.16579900	0.42604300
C	-0.56070900	2.29843000	-1.60760500
C	-4.08950500	0.52394200	-0.43582800
C	-2.54333500	-0.61218600	-2.07678000
C	-3.07367400	-1.73867000	0.12606100
H	-3.23896300	1.45675300	2.09901100
C	-2.78951100	0.77055900	4.07872700
C	-0.84104100	-0.64840900	4.28757300
N	0.53862200	-1.26419400	2.34549200
H	0.42531600	2.20460900	1.74573800
H	1.32400900	1.61317100	0.32471100
C	1.09670600	3.77089300	0.37527800
C	-1.27433700	4.59371000	0.12127600
H	-1.93884000	3.06332800	1.51142100
H	-2.77836500	3.01862100	-0.05966800
C	-0.02008700	3.72266400	-1.89009300
H	-1.51316100	2.16558500	-2.14116700
H	0.14743200	1.53917300	-1.97809500
H	-4.32736900	0.71265800	0.61859000
H	-3.92280700	1.48978900	-0.93496100
C	-5.30028700	-0.19601200	-1.09040100
C	-3.75484300	-1.32311700	-2.73010400
H	-2.35185500	0.33901200	-2.59096500
H	-1.64723800	-1.24097600	-2.19640300
C	-4.28461000	-2.44322100	-0.53262000
H	-3.26768500	-1.59167300	1.19865800
H	-2.17252500	-2.36877500	0.02701600
H	-3.63401700	1.29777500	4.52191000
C	-1.92264400	0.01281600	4.87873400
H	-0.15640100	-1.23070700	4.90422700
C	0.50463900	-2.71691100	2.57850100
C	1.81105900	-0.65889300	2.77626800
H	2.04958900	3.90549500	0.90998500
C	0.06345500	4.81080000	0.86567100
C	1.32046300	3.92834200	-1.14710000
H	-2.02117300	5.32018800	0.47518200
C	-1.05690000	4.76093300	-1.40019900
H	0.13253400	3.82878800	-2.97478200
H	-6.18548100	0.44740100	-0.97535700
C	-5.53876100	-1.54969100	-0.38060200
C	-5.01302700	-0.43618500	-2.58882600
C	-3.99046400	-2.68200300	-2.03105200
H	-3.52754800	-1.48381200	-3.79490000
H	-4.44794600	-3.40519300	-0.02338400
H	-2.08360900	-0.05766500	5.95462300
H	1.34461900	-3.16893300	2.03451100
H	-0.43476200	-3.11995100	2.17756200
H	0.58581000	-2.98948000	3.64871800
H	1.98484700	-0.75974700	3.86530900
H	1.81087900	0.40546300	2.51072900
H	2.62764600	-1.15483800	2.23576300
H	0.43660400	5.82872600	0.67425200
H	-0.08836100	4.70865700	1.95248600
H	2.06170300	3.19071400	-1.49595800
H	1.71714600	4.93194300	-1.36529000
H	-2.00992800	4.62334200	-1.93690700
H	-0.69794600	5.77922400	-1.61546200
H	-6.41138900	-2.05305200	-0.82461800
H	-5.75640500	-1.38479600	0.68726900
H	-4.85711200	0.52587400	-3.10346500
H	-5.87566800	-0.93146200	-3.06057100
H	-3.09990400	-3.32304900	-2.14277000
H	-4.83835300	-3.20344600	-2.50135100

Sum of electronic and zero-point Energies= -1864.593536
Sum of electronic and thermal Free Energies= -1864.662368

TS1*$_1$

C	3.33632000	-0.00990600	-0.19474800
C	4.05162500	0.70339800	0.79858000
H	3.81056800	1.74521100	1.00229800
I	2.56955700	1.34662100	-2.06160300
Au	1.13096800	0.03035600	0.05075100
C	3.69131400	-1.33787300	-0.54065500
H	3.17951800	-1.84338700	-1.35780300
C	4.70195600	-1.98016900	0.18855100
H	4.96638300	-3.00875400	-0.05742600
C	5.39193600	-1.29191700	1.20077300
H	6.19949200	-1.78683100	1.73957100
C	5.06160800	0.04169700	1.50746100
H	5.60635800	0.57693500	2.28572200
P	-1.16654300	-0.17188600	0.34934900
C	-1.45348500	-0.77728800	2.05921600
C	-1.97467500	1.51953500	0.21434700
C	-1.81147400	-1.48598300	-0.84776300
C	-2.75956500	-1.10302000	2.49213300
C	-0.37611700	-0.97619600	2.95978700
C	-1.13666300	2.49176200	1.10120200
C	-3.44800600	1.56504000	0.69966300
C	-1.88826700	1.98690200	-1.26444400
C	-3.35258900	-1.49192200	-1.03869900
C	-1.13533800	-1.28482500	-2.24087000
C	-1.34704600	-2.85983400	-0.27796600
H	-3.59959100	-0.95561000	1.81947200
C	-3.00046300	-1.60871900	3.77403500
C	-0.62973800	-1.47940100	4.24932900
N	0.99344300	-0.65825500	2.59439500
H	-1.17302400	2.15825600	2.15112900
H	-0.08348900	2.48624700	0.77585700
C	-1.69314800	3.93189700	0.97689800
C	-4.00142000	3.00648700	0.55076400
H	-3.49586100	1.27233700	1.75795100
H	-4.06553300	0.86456100	0.12114200
C	-2.43385200	3.43166800	-1.38687800
H	-2.48921500	1.32140900	-1.90085900
H	-0.84215000	1.93612000	-1.60768400
H	-3.86126900	-1.63221700	-0.07509600
H	-3.67981400	-0.53106800	-1.46188600
C	-3.75846300	-2.64261400	-1.99665100
C	-1.55548800	-2.42281600	-3.20410700
H	-1.41135800	-0.31147300	-2.66723100
H	-0.03992600	-1.29605000	-2.13116800
C	-1.75949500	-3.99778500	-1.24378200
H	-1.79895500	-3.03379700	0.70912200
H	-0.24985700	-2.84812100	-0.14960400
H	-4.01829200	-1.85123100	4.07844800
C	-1.92888700	-1.79815500	4.65866400
H	0.20596000	-1.61886700	4.93482700

C	1.90901500	-1.81243800	2.70086700
C	1.49962100	0.52150800	3.32325000
H	-1.08819200	4.59688300	1.61203500
C	-3.16907700	3.96131600	1.43720100
C	-1.59653200	4.38267100	-0.50010500
H	-5.05146200	3.01119200	0.87964400
C	-3.91173100	3.45349000	-0.92738600
H	-2.36370100	3.74388600	-2.43970700
H	-4.85355000	-2.63436200	-2.10441200
C	-3.29941500	-3.99501400	-1.40146000
C	-3.09216300	-2.42149900	-3.37328300
C	-1.09409900	-3.77877800	-2.62121500
H	-1.07102200	-2.24854500	-4.17672500
H	-1.43036000	-4.95651900	-0.81497800
H	-2.10275500	-2.18842600	5.66143100
H	2.87717700	-1.51541100	2.28198100
H	1.50097600	-2.64306200	2.11165200
H	2.05096200	-2.14079300	3.74634000
H	1.56210800	0.33699600	4.41120900
H	0.82948700	1.36975100	3.13563200
H	2.50115400	0.75753600	2.94023500
H	-3.56354000	4.98619400	1.35931100
H	-3.24234700	3.65455300	2.49340200
H	-0.54429000	4.37617700	-0.82856400
H	-1.97159400	5.41282600	-0.60075600
H	-4.51278500	2.78178800	-1.56211900
H	-4.32233500	4.46918500	-1.03537800
H	-3.60792100	-4.81804100	-2.06425700
H	-3.77797700	-4.15815900	-0.42189000
H	-3.42446900	-1.46264100	-3.80349800
H	-3.39344100	-3.22073200	-4.06792700
H	0.00379200	-3.78836100	-2.51541500
H	-1.36999300	-4.59583700	-3.30566700

Sum of electronic and zero-point Energies= -1864.578796
Sum of electronic and thermal Free Energies= -1864.645754

2*$_1$

C	3.39852300	0.03836600	0.03598800
C	4.13142000	1.11005700	0.57406300
H	3.61866900	1.99732700	0.94782200
I	1.52613300	1.40695800	-2.15779800
Au	1.30234700	0.02505300	0.12207800
C	4.06051900	-1.06596400	-0.52815300
H	3.49466100	-1.86866100	-1.00417500
C	5.46395800	-1.13626200	-0.47034900
H	5.97665200	-2.00011900	-0.89524700
C	6.19849800	-0.09075200	0.11045300
H	7.28702500	-0.14092500	0.13943200
C	5.53406100	1.03221500	0.62848300
H	6.10195900	1.85819200	1.05840100
P	-1.07555400	-0.17628400	0.38575900
C	-1.18215500	-0.90453900	2.07319000
C	-1.99011300	1.47267600	0.38972200
C	-1.74200900	-1.50512400	-0.77009000
C	-2.42201700	-1.22755700	2.67236000
C	-0.00199900	-1.24866300	2.76215200
C	-1.01013900	2.50893900	1.02418200
C	-3.30378600	1.46605600	1.22105800
C	-2.31818300	1.90204400	-1.07014800
C	-3.28181500	-1.68214500	-0.69651700
C	-1.31373500	-1.17799100	-2.23009900
C	-1.04753100	-2.84000400	-0.35975500
H	-3.34387100	-0.98162400	2.15582100
C	-2.48894200	-1.85013200	3.92247600
C	-0.06863400	-1.86960000	4.02218500
N	1.34741600	-0.98947600	2.19090400
H	-0.76551900	2.20390000	2.05635100
H	-0.07564800	2.54634500	0.44460900
C	-1.65809800	3.91656700	1.02281700
C	-3.94977600	2.87628000	1.20176100
H	-3.08306700	1.19779900	2.26335300
H	-4.00924100	0.72610200	0.81241100
C	-2.94750000	3.31891500	-1.06797900
H	-3.03089000	1.19188600	-1.51408500
H	-1.40794200	1.90217400	-1.68288600
H	-3.58345200	-1.94603700	0.32685000
H	-3.78300200	-0.74269800	-0.97107100
C	-3.71880400	-2.81999600	-1.65613700
C	-1.77212700	-2.31116600	-3.18046900
H	-1.74021000	-0.22222900	-2.55753800
H	-0.22044800	-1.08432300	-2.27496000
C	-1.49647300	-3.97894400	-1.30989200
H	-1.30351600	-3.10178300	0.67756200
H	0.04860300	-2.71415700	-0.42233600
H	-3.45850200	-2.08455900	4.36060800
C	-1.30546400	-2.16798300	4.60278300
H	0.84570400	-2.12732900	4.55375100
C	2.09634300	-2.28210800	2.03025800
C	2.10975200	-0.05578000	3.08766800
H	-0.94841800	4.62668400	1.47366800
C	-2.97015300	3.88897200	1.83904100
C	-1.95771900	4.32758200	-0.43882700
H	-4.87997300	2.83582300	1.78841800
C	-4.26046500	3.29250200	-0.25384400
H	-3.15674800	3.60288200	-2.11010800
H	-4.81177800	-2.92925000	-1.59098000
C	-3.03357300	-4.13867100	-1.22305600
C	-3.30871800	-2.46466400	-3.10390800
C	-1.09100700	-3.63376800	-2.76071600
H	-1.47179200	-2.04390100	-4.20452000
H	-1.00548700	-4.91150500	-0.99221800
H	-1.33928800	-2.64997400	5.57935000
H	3.07210200	-2.05706800	1.58947300
H	1.51950900	-2.93088000	1.36202000
H	2.22728500	-2.76790200	3.00690300
H	2.21857800	-0.49885400	4.08702800
H	1.55396200	0.88633900	3.15338600
H	3.09459800	0.11703200	2.64401800
H	-3.42401100	4.89173300	1.84990600
H	-2.76061700	3.60525200	2.88356100
H	-1.02353400	4.35334100	-1.02231300
H	-2.39385800	5.33822600	-0.45854800
H	-4.97207100	2.58297500	-0.70705400
H	-4.73023500	4.28806000	-0.26452100
H	-3.35758300	-4.96044900	-1.87968300
H	-3.32972400	-4.39709800	-0.19313800
H	-3.79813200	-1.52759200	-3.41556100
H	-3.64127700	-3.25805700	-3.79085100
H	0.00444800	-3.53126500	-2.83388400
H	-1.39579500	-4.44734200	-3.43680400

Sum of electronic and zero-point Energies= -1864.592904
Sum of electronic and thermal Free Energies= -1864.659028

Supplementary Discussion.

Comment on DFT calculations for the oxidative addition of PhI to 1

The mechanism of the reaction and the key role of the (P,N) ligand on the oxidative addition process were examined computationally with the real complex **1** in taking into account the counter–anion and solvent effects (Supplementary Figure 49). The first step of the reaction is the displacement of the weakly coordinating counter anion SbF_6^- by iodobenzene to form the corresponding linear two–coordinate I-adduct (no coordination of nitrogen, dAu–N = 2.863 Å), which is slightly downhill in energy ($\Delta G = -3$ kcal.mol^{-1}). The oxidative addition of PhI then proceeds with a low activation barrier ($\Delta G^{\neq} = 11.2$ kcal.mol^{-1}) *via* the 3-center transition state **TS1**. The geometric features of **TS1** (Supplementary table 7) indicate a short C_{ipso} to Au distance with an elongation of the C–I bond (2.438 Å *vs* 2.184 Å in the corresponding PhI adduct), the nitrogen atom of the (P,N) ligand is also found to get closer to the Au center (dAu-N = 2.670 Å). The process is favored thermodynamically ($\Delta G_{\mathbf{1\text{-}SbF6}\rightarrow\mathbf{2\text{-}SbF6}} = -8.9$ kcal.mol^{-1} or $\Delta G_{\mathbf{I\text{-}adduct}\rightarrow\mathbf{2\text{-}SbF6}} = -5.9$ kcal.mol^{-1}) and leads to the 4–coordinate gold(III) aryl complex **2** (dAu–N = 2.350 Å) we obtained experimentally (the optimized structure matches very well with that determined crystallographically). In comparison, NTf_2^- binds more strongly to gold ($\Delta G_{\mathbf{1\text{-}NTf2}\rightarrow\mathbf{I\text{-}adduct}} = 11.3$ kcal.mol^{-1}, Supplementary Figure 49). Its displacement by PhI requires an activation barrier ($\Delta G^{\neq} = 18.9$ kcal.mol^{-1}, Supplementary Figure 49) which is significantly higher than that of the oxidative addition step ($\Delta G^{\neq} = 13.8$ kcal.mol^{-1}).[49] This explains the strong counter anion effect, the rate of the reaction being significantly slower with NTf_2^- than SbF_6^- .The formation of the other gold(III) diastereomer with the phenyl ring in *cis* position to nitrogen was also investigated theoretically (Supplementary Figure 50). The reaction is less favored thermodynamically and requires a higher activation barrier.

To further analyze the impact of the adjacent nitrogen atom, the reaction profile for oxidative addition of iodobenzene to the related gold complex devoid of NMe_2 $\mathbf{1_H\text{-}SbF_6}$ substituent was then computed (Supplementary Figure 51). The transformation is still kinetically feasible, but the corresponding activation barrier is about twice as large. Moreover, the reaction is not favored thermodynamically ($\Delta G_{\mathbf{1H\text{-}SbF6}\rightarrow\mathbf{2H\text{-}SbF6}} = 7.5$ kcal.mol^{-1} or $\Delta G_{\mathbf{I\text{-}adduct}\rightarrow\mathbf{2H\text{-}SbF6}} = 11.2$ kcal.mol^{-1}) due to the formation of a high–energy 3–coordinate gold(III) species. This comparison highlights the critical role of the hemilabile (P,N) ligand that lowers the activation barrier for oxidative addition and thermodynamically stabilizes the resulting gold(III) complex by coordination of the nitrogen atom.

Supplementary References

[1.]Winston, M. S., Wolf, W. J. & Toste, F. D. Halide-Dependent Mechanisms of Reductive Elimination from Gold(III). *J. Am. Chem. Soc.* **137**, 7921–7928 (2015).

[2.] Dai, J. J., Liu, J. H., Luo, D. F. & Liu, L. Pd-catalysed decarboxylative Suzuki reactions and orthogonal Cu-based O-arylation of aromatic carboxylic acids. *Chem. Commun.* **47**, 677–679 (2011).

[3.] Zhou, Z. et al. Palladium-Catalyzed Suzuki-Miyaura Coupling Reactions of Boronic Acid Derivatives with Aryl Chlorides. *Asian J. Org. Chem.* **5**, 1260–1268 (2016).

[4.] Dennis, E. G., Jeffery, D. W., Perkins, M. V. & Smith, P. A. Pd(DPEPhos)Cl2-catalyzed Negishi cross-couplings for the formation of biaryl and diarylmethane phloroglucinol adducts. *Tetrahedron* **67**, 2125–2131 (2011).

[5.] Mino, T. et al. Palladium-catalyzed decarboxylative coupling of benzoic acid derivatives using hydrazone ligands. *Tetrahedron Lett.* **55**, 3184–3188 (2014).

[6.] Wang, X. et al. Copper(II) triflate-catalyzed direct arylation for synthesis of biphenyls using diaryliodonium salts as arylating reagents. *Tetrahedron Lett.* **57**, 4235–4238 (2016).

[7.]Bunrit, A., Sawadjoon, S., Tšupova, S., Sjöberg, P. J. R. & Samec, J. S. M. A General Route to +¦- Substituted Pyrroles by Transition-Metal Catalysis. *J. Org. Chem.* **81**, 1450–1460 (2016).

[8.]Truong, T., Daugulis, O. Base-Mediated Intermolecular sp2 C-H Bond Arylation via Benzyne Intermediates. *J. Am. Chem. Soc.* **133**, 4243–4245 (2011).

[9] SADABS. Bruker AXS Inc., Madison, Wisconsin, USA.

[10] G. M. Sheldrick, *Acta Cryst.* **64**, 112–122 *A (*2007),

[11] Grimme, S. Semiempirical GGA-type density functional constructed with a long-range dispersion correction. *J. Comput. Chem.* **27**, 1787–1799 (2006).

[12] Gaussian 09, Revision D.01, Frisch, M. J.; Trucks, G. W.; Schlegel, H. B.; Scuseria, G. E.; Robb, M. A.; Cheeseman, J. R.; Scalmani, G.; Barone, V.; Mennucci, B.; Petersson, G. A.; Nakatsuji, H.; Caricato, M.; Li, X.; Hratchian, H. P.; Izmaylov, A. F.; Bloino, J.; Zheng, G.; Sonnenberg, J. L.; Hada, M.; Ehara, M.; Toyota, K.; Fukuda, R.; Hasegawa, J.; Ishida, M.; Nakajima, T.; Honda, Y.; Kitao, O.; Nakai, H.; Vreven, T.; Montgomery, J. A., Jr. ; Peralta, J. E.; Ogliaro, F.; Bearpark, M.; Heyd, J. J.; Brothers, E.; Kudin, K. N.; Staroverov, V. N.; Kobayashi, R.; Normand, J.; Raghavachari, K.; Rendell, A.; Burant, J. C.; Iyengar, S. S.; Tomasi, J.; Cossi, M.; Rega, N.; Millam, J. M.; Klene, M.; Knox, J. E.; Cross, J. B.; Bakken, V.; Adamo, C.; Jaramillo, J.; Gomperts, R.; Stratmann, R. E.; Yazyev, O.; Austin, A. J.; Cammi, R.; Pomelli, C.; Ochterski, J. W.; Martin, R. L.; Morokuma, K.; Zakrzewski, V. G.; Voth, G. A.; Salvador, P.; Dannenberg, J. J.; Dapprich, S.; Daniels, A. D.; Farkas, O .; Foresman, J. B.; Ortiz, J. V.; Cioslowski, J.; Fox, D. J. Gaussian 09; Gaussian, Inc., Wallingford, CT, (2009).

[13] Andrae, D.; Häussermann, U.; Dolg, M.; Stoll, H.; Preuss, H. Energy-adjustedab initio pseudopotentials for the second and third row transition elements *Theor. Chim. Acta* **77, 123–141 (1990).**

[14] Ehlers, A.W.; Biihme, M.; Dapprich, S.; Gobbi, A.; Hijllwarth, A.; Jonas, V.; Kiihler, K.F.; Stegmann, R.; Veldkamp, A.; Frenking, G. Ehlers, A. W. et al. A set of f-polarization functions for pseudo-potential basis sets of the transition metals Sc-Cu, Y-Ag and La-Au. **208**, 111–114 (1993).

[15] Marenich, A. V.; Cramer, C. J.; Truhlar, D. G., Universal Solvation Model Based on Solute Electron Density and on a Continuum Model of the Solvent Defined by the Bulk Dielectric Constant and Atomic Surface Tensions. *J. Phys. Chem. B* ***113**, 6378–6396 (2009).*

[16] Fukui, K. The path of chemical reactions - the IRC approach. *Acc. Chem. Res.* **14**, 363–368 (1981).
[17] Hratchian, H. P.; Schlegel, H. B. Finding minima, transition states, and following reaction pathways on ab initio potential energy surfaces. In *Theory and Applications of Computational Chemistry: The First 40 Years*; Dykstra, C. E., Frenking, G., Kim, K. S., Scuseria, G., Eds.; Elsevier: Amsterdam, 195, (2005).
